# Supplementary material for: Can participatory approaches strengthen the monitoring of cyanobacterial blooms in developing countries? Results from a pilot study conducted in the Lagoon Aghien (Ivory Coast)
Source: PLoS One. 2020 Sep 24;15(9):e0238832. doi: 10.1371/journal.pone.0238832 (PMC7514105; doi:10.1371/journal.pone.0238832)
Supplement: S1 Fig — (PPTX) [file pone.0238832.s001.pptx]

## Slide 1
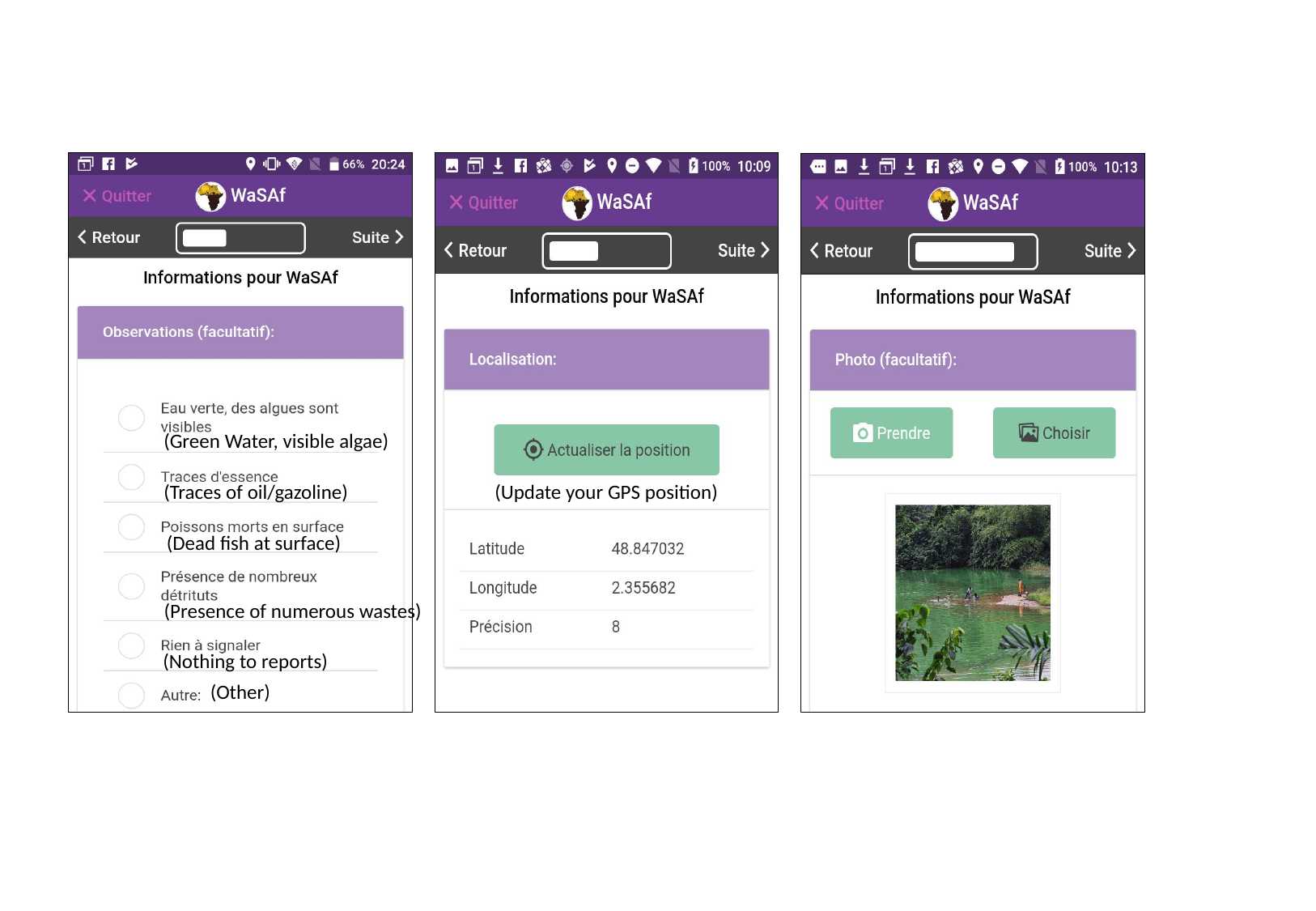

(Green Water, visible algae)
(Update your GPS position)
(Traces of oil/gazoline)
(Dead fish at surface)
(Presence of numerous wastes)
(Nothing to reports)
(Other)
