# Supplementary figures and images for: Can participatory approaches strengthen the monitoring of cyanobacterial blooms in developing countries? Results from a pilot study conducted in the Lagoon Aghien (Ivory Coast)
Source: PLoS One. 2020 Sep 24;15(9):e0238832. doi: 10.1371/journal.pone.0238832 (PMC7514105; doi:10.1371/journal.pone.0238832)

## Slide 1
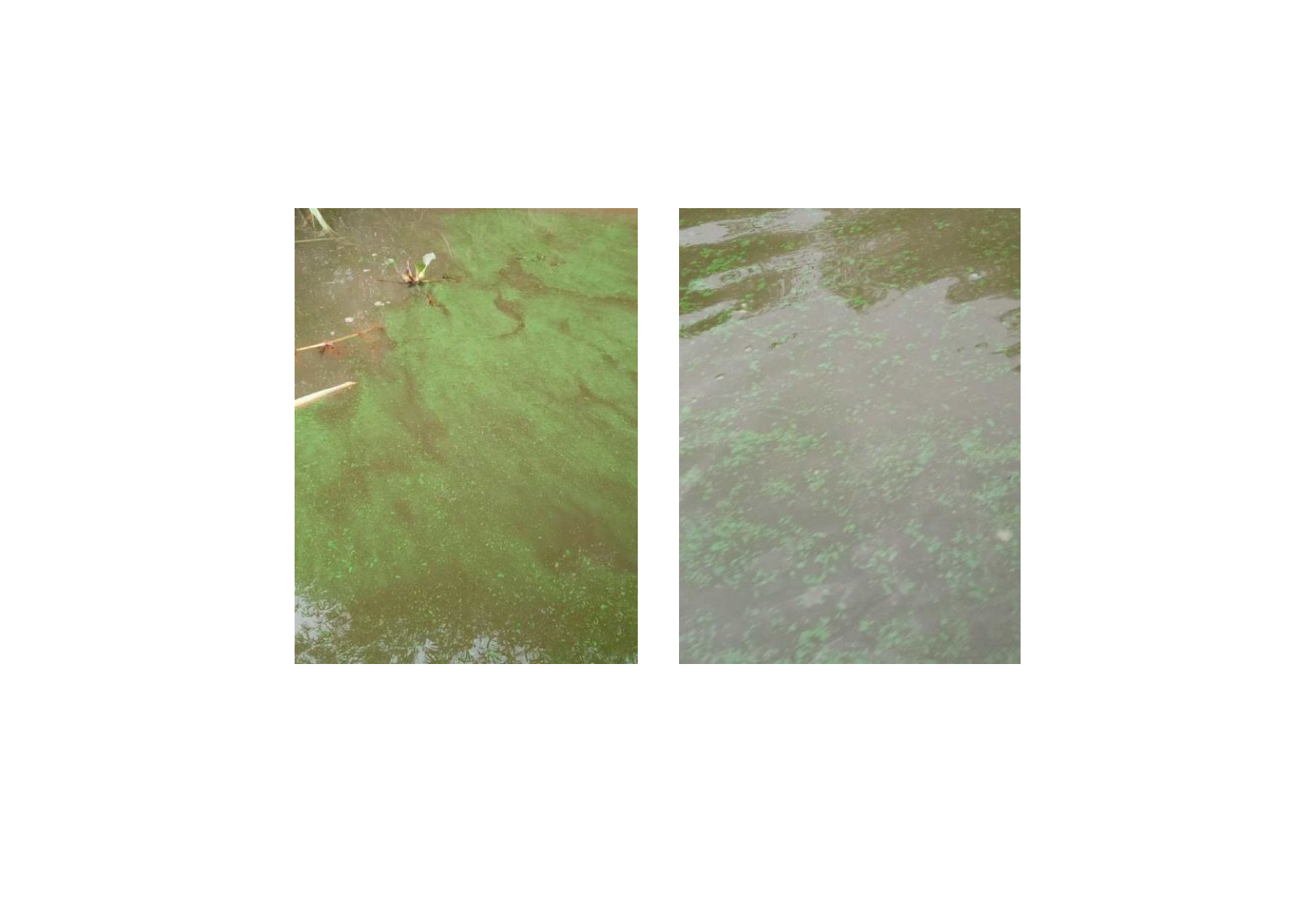

Supplement: S2 Fig — (PPTX) [file pone.0238832.s002.pptx]
