# Supplementary material for: Can participatory approaches strengthen the monitoring of cyanobacterial blooms in developing countries? Results from a pilot study conducted in the Lagoon Aghien (Ivory Coast)
Source: PLoS One. 2020 Sep 24;15(9):e0238832. doi: 10.1371/journal.pone.0238832 (PMC7514105; doi:10.1371/journal.pone.0238832)
Supplement: S1 Table — (DOCX) [file pone.0238832.s003.docx]

1. Comment qualifierez-vous l’état actuel de la lagune :

1. Très bon état
2. Bon état
3. Mauvais état
4. Très mauvais état

*1. How would you qualify the current state of the lagoon :*

1. *Very good state*
2. *Good state*
3. *Bad state*
4. *Very bad state*

2. En fonction de quels indices jugez-vous de l’état écologique de la lagune ? (Plusieurs choix possibles)

1. Baisse des captures lors de la pêche
2. Disparition de certaines espèces aquatiques
3. L'eau de la lagune change de couleur
4. Les odeurs se dégagent de l'eau
5. Prolifération de roseaux
6. Déchets solides drainés par les eaux de pluie
7. L'eau de plus en plus en retrait
8. Autres signes (Lesquels ?….)
9. NSP

*2. Based on which criteria do you judge about the ecological quality of the lagoon? (Several choices possible)*

1. *Significant drop of fishermen's production*
2. *Extinction of certain aquatic species*
3. *Changes in water color*
4. *Odors emanating from the water*
5. *Reed proliferation*
6. *Solid waste drained by rainwater*
7. *Decrease of the water level*
8. *Other criteria (Which ones?…..)*
9. *I don’t know (no answer)*
