# Supplementary material for: Can participatory approaches strengthen the monitoring of cyanobacterial blooms in developing countries? Results from a pilot study conducted in the Lagoon Aghien (Ivory Coast)
Source: PLoS One. 2020 Sep 24;15(9):e0238832. doi: 10.1371/journal.pone.0238832 (PMC7514105; doi:10.1371/journal.pone.0238832)
Supplement: Attachment — Submitted filename: PONE-D-20-09032_reviewer 2.COMMENTS (1).pdf [file pone.0238832.s004.pdf]

PLOS ONE

Can participatory approaches strengthen the monitoring of cyanobacterial blooms in developing countries? Results from a pilot study conducted in the Lagoon Aghien (Ivory Coast)

--Manuscript Draft--

**Manuscript Number:**	PONE-D-20-09032
**Article Type:**	Research Article
**Full Title:**	Can participatory approaches strengthen the monitoring of cyanobacterial blooms in developing countries? Results from a pilot study conducted in the Lagoon Aghien (Ivory Coast)
**Short Title:**	Citizen monitoring of cyanobacteria in Africa
**Corresponding Author:**	Jean-François Humbert<br>INRA<br>Paris, UPMC FRANCE
**Keywords:**	Citizen monitoring; cyanobacteria; Lagoon Aghien; Ivory Coast
**Abstract:**	Monitoring of cyanobacteria in freshwater ecosystems is often complex to organize, time consuming and expensive due to the chaotic population dynamics and highly heterogeneous distribution of cyanobacteria populations in water bodies. This financial cost constitutes a strong limitation for the implementation of long-term monitoring programs in developing countries, particularly in Africa, where it is challenging to mobilize sustainable financing for such programs. The work presented here was performed in the framework of an international project addressing the sustainable monitoring and management of surface water resources used for the production of drinking water in three African countries. We evaluated the efficiency of a citizen approach for monitoring cyanobacterial blooms. This approach was designed, implemented and evaluated in close interaction with the Pasteur Institute of the Ivory Coast and with the populations of three villages located on the shoreline of a freshwater lagoon located near Abidjan city. Based on the use of a smartphone application, the citizens of the three villages were invited to report water color changes, as these changes could reflect cyanobacteria proliferation . After a two-year experimentation period, the local population appeared to provide accurate data on the development of cyanobacteria in the lagoon. These data were congruent with those obtained by classical monitoring (water sampling and cell counting) performed over seven months. In addition, the participatory approach also provided great improvements to the understanding and awareness of local populations regarding water quality and cyanobacterial bloom issues. Finally, we discuss some of the difficulties and limitations of our participatory monitoring approach that should be considered by further implementations. Despite these difficulties, our work suggests that citizen monitoring is a promising approach that may complement the classical approach to sustainable monitoring of cyanobacteria in developing countries.
**Order of Authors:**	Veronica Mitroi
	Kouadio Chrislain Ahi
	Pierre Yves Bulot
	Fulbert Tra
	José Frédéric Deroubaix
	Mathias Koffi Ahoutou
	Catherine Quiblier
	Mariatou Koné
	Julien Coulibaly Kalpy
	Jean-François Humbert
**Additional Information:**	

Powered by Editorial Manager® and ProduXion Manager® from Aries Systems Corporation

Question	Response
**Financial Disclosure**<br><br>Enter a financial disclosure statement that describes the sources of funding for the work included in this submission. Review the submission guidelines for detailed requirements. View published research articles from *PLOS ONE* for specific examples.<br><br>This statement is required for submission and **will appear in the published article** if the submission is accepted. Please make sure it is accurate.<br><br>**Unfunded studies**<br>Enter: *The author(s) received no specific funding for this work.*<br><br>**Funded studies**<br>Enter a statement with the following details:<br>• Initials of the authors who received each award<br>• Grant numbers awarded to each author<br>• The full name of each funder<br>• URL of each funder website<br>• Did the sponsors or funders play any role in the study design, data collection and analysis, decision to publish, or preparation of the manuscript?<br>• **NO** - Include this sentence at the end of your statement: *The funders had no role in study design, data collection and analysis, decision to publish, or preparation of the manuscript.*<br>• **YES** - Specify the role(s) played.<br><br>* typeset	VM, French Facility for Global Environment, https://www.ffem.fr/en<br>KCA, French Facility for Global Environment, https://www.ffem.fr/en<br>MKA, French Facility for Global Environment, https://www.ffem.fr/en<br><br>NO
**Competing Interests**<br><br>Use the instructions below to enter a competing interest statement for this submission. On behalf of all authors, disclose any competing interests that could be perceived to bias this work—acknowledging all financial support and any other relevant financial or non-financial competing interests.<br><br>This statement **will appear in the published article** if the submission is	NO authors have competing interests

accepted. Please make sure it is accurate. View published research articles from *PLOS ONE* for specific examples.<br><br>**NO authors have competing interests**<br><br>Enter: *The authors have declared that no competing interests exist.*<br><br>**Authors with competing interests**<br><br>Enter competing interest details beginning with this statement:<br><br>*I have read the journal's policy and the authors of this manuscript have the following competing interests: [insert competing interests here]*<br><br>* typeset	
**Ethics Statement**<br><br>Enter an ethics statement for this submission. This statement is required if the study involved:<br><br>• Human participants<br>• Human specimens or tissue<br>• Vertebrate animals or cephalopods<br>• Vertebrate embryos or tissues<br>• Field research<br><br>Write "N/A" if the submission does not require an ethics statement.<br><br>General guidance is provided below. Consult the submission guidelines for detailed instructions. **Make sure that all information entered here is included in the Methods section of the manuscript.**	This study was not submitted for approval by an institutional review board (ethics committee) because there is currently no committee in Ivory Coast for environmental studies. But as described latter in the paper, we organized in each of the three villages concerned by this study, a meeting with the chiefdoms in order to present the project and to ask them for their agreement before to begin to work. In addition we asked also to the chiefdoms to designate the sentinels (referent people) in each village who have provided most of the data. Finally, our smartphone application entailed the possibility to collect anonymous reports.

Powered by Editorial Manager® and ProduXion Manager® from Aries Systems Corporation

**Format for specific study types**<br><br>**Human Subject Research (involving human participants and/or tissue)**<br>• Give the name of the institutional review board or ethics committee that approved the study<br>• Include the approval number and/or a statement indicating approval of this research<br>• Indicate the form of consent obtained (written/oral) or the reason that consent was not obtained (e.g. the data were analyzed anonymously)<br><br>**Animal Research (involving vertebrate animals, embryos or tissues)**<br>• Provide the name of the Institutional Animal Care and Use Committee (IACUC) or other relevant ethics board that reviewed the study protocol, and indicate whether they approved this research or granted a formal waiver of ethical approval<br>• Include an approval number if one was obtained<br>• If the study involved *non-human primates*, add *additional details* about animal welfare and steps taken to ameliorate suffering<br>• If anesthesia, euthanasia, or any kind of animal sacrifice is part of the study, include briefly which substances and/or methods were applied<br><br>**Field Research**<br><br>Include the following details if this study involves the collection of plant, animal, or other materials from a natural setting:<br>• Field permit number<br>• Name of the institution or relevant body that granted permission	
**Data Availability**<br><br>Authors are required to make all data underlying the findings described fully available, without restriction, and from the time of publication. PLOS allows rare exceptions to address legal and ethical concerns. See the PLOS Data Policy and FAQ for detailed information.	Yes - all data are fully available without restriction

A Data Availability Statement describing where the data can be found is required at submission. Your answers to this question constitute the Data Availability Statement and **will be published in the article**, if accepted.<br><br>**Important:** Stating 'data available on request from the author' is not sufficient. If your data are only available upon request, select 'No' for the first question and explain your exceptional situation in the text box.<br><br>Do the authors confirm that all data underlying the findings described in their manuscript are fully available without restriction?	
**Describe where the data may be found in full sentences. If you are copying our sample text, replace any instances of XXX with the appropriate details.**<br><br>• If the data are **held or will be held in a public repository**, include URLs, accession numbers or DOIs. If this information will only be available after acceptance, indicate this by ticking the box below. For example: *All XXX files are available from the XXX database (accession number(s) XXX, XXX.)*.<br>• If the data are all contained **within the manuscript and/or Supporting Information files**, enter the following: *All relevant data are within the manuscript and its Supporting Information files.*<br>• If neither of these applies but you are able to provide **details of access elsewhere**, with or without limitations, please do so. For example:<br><br>*Data cannot be shared publicly because of [XXX]. Data are available from the XXX Institutional Data Access / Ethics Committee (contact via XXX) for researchers who meet the criteria for access to confidential data.*<br><br>*The data underlying the results presented in the study are available from (include the name of the third party*	All data obtained by the citizen monitroing are available on the website: https://five.epicollect.net/project/wasaf

and contact information or URL).<br>• This text is appropriate if the data are owned by a third party and authors do not have permission to share the data.<br><br>* typeset	
Additional data availability information:	

Powered by Editorial Manager® and ProduXion Manager® from Aries Systems Corporation

Manuscript
Click here to access/download;Manuscript;Manuscript.docx

Can participatory approaches strengthen the monitoring of cyanobacterial blooms in developing countries? Results from a pilot study conducted in the Lagoon Aghien (Ivory Coast)

Veronica Mitroi[1], Kouadio Chrislain Ahi[2], Pierre-Yves Bulot[1,3], Fulbert Tra[2], José-Frédéric Deroubaix[4], Mathias Koffi Ahoutou[5,6], Catherine Quiblier[7,8], Mariatou Koné[2], Julien Coulibaly Kalpy[5], Jean-François Humbert[1*]

1. INRAE-Sorbonne Université, iEES Paris, Paris, France
2. Université Félix Houphouët Boigny, Institut d'Ethnosociologie, Abidjan, Côte d'Ivoire
3. INRAE-LISIS, Cité Descartes, Marne-la-Vallée, France
4. LEESU, École des Ponts ParisTech, Champs-sur-Marne, France
5. Institut Pasteur d'Abidjan, Abidjan, Côte d'Ivoire
6. Université Jean Lorougnon Guédé, Daloa, Côte d'Ivoire
7. MNHN, UMR 7245 Molécules de Communication et Adaptation des Micro-organismes, Paris, France
8. Université de Paris, Paris, France

* Corresponding author, jean-francois.humbert@upmc.fr

Short Title: Citizen monitoring of cyanobacteria in Africa

Key words: Citizen monitoring, cyanobacteria, Lagoon Aghien, Ivory Coast

Abstract

Monitoring of cyanobacteria in freshwater ecosystems is often complex to organize, time consuming and expensive due to the chaotic population dynamics and highly heterogeneous distribution of cyanobacteria populations in water bodies. This financial cost constitutes a strong limitation for the implementation of long-term monitoring programs in developing countries, particularly in Africa, where it is challenging to mobilize sustainable financing for such programs. The work presented here was performed in the framework of an international project addressing the sustainable monitoring and management of surface water resources used for the production of drinking water in three African countries. We evaluated the efficiency of a citizen approach for monitoring cyanobacterial blooms. This approach was designed, implemented and evaluated in close interaction with the Pasteur Institute of the Ivory Coast and with the populations of three villages located on the shoreline of a freshwater lagoon located near Abidjan city. Based on the use of a smartphone application, the citizens of the three villages were invited to report water color changes, as these changes could reflect cyanobacteria proliferation. After a two-year experimentation period, the local population appeared to provide accurate data on the development of cyanobacteria in the lagoon. These data were congruent with those obtained by classical monitoring (water sampling and cell counting) performed over seven months. In addition, the participatory approach also provided great improvements to the understanding and awareness of local populations regarding water quality and cyanobacterial bloom issues. Finally, we discuss some of the difficulties and limitations of our participatory monitoring approach that should be considered by further implementations. Despite these difficulties, our work suggests that citizen monitoring is a promising approach that may complement the classical approach to sustainable monitoring of cyanobacteria in developing countries.

Introduction

Monitoring the proliferation of potentially toxic cyanobacteria in freshwater ecosystems is an essential pillar for the assessment and management of sanitary risks associated with these phenomena. Numerous studies have therefore been conducted on this issue over the last twenty years. It has been shown that (i) one of the main difficulties in cyanobacteria monitoring is due to the temporal and spatial variability in their vertical and horizontal distributions in water bodies [e.g. 1-3], meaning that the choice of sampling strategy and the tools used for sampling have great impacts on the quality of the data collected [3,4], and (ii) accurate monitoring of cyanobacteria and their associated risks is time consuming and expensive [5].

As a matter of consequence, many efforts have been made to develop and validate new innovative devices for the survey of cyanobacterial blooms. For example, probes equipped with chlorophyll-a/phycocyanin and other sensors that can be coupled with autonomous monitoring platforms are now available for punctual or continuous estimations of cyanobacteria biomasses in the water column [6,7]. In the same way, the use of remote sensing [8] and LIDAR [9] allows us to estimate the horizontal and/or vertical distribution of the cyanobacterial biomass in a lake. Some of these tools remain under development but are increasingly used for cyanobacteria monitoring, particularly in developed countries. On the other hand, in developing countries, the cost of these devices is still a strong limitation to their use [10].

Another strategy recently tested for the monitoring of water quality in freshwater ecosystems is the development of citizen science projects based on the involvement of the population living around the targeted freshwater ecosystems [11]. Some of these projects are specifically concerned with the monitoring of cyanobacterial blooms as blooms are easy to report by nonscientist actors due to changes occurring in the water color or the presence of colonies or aggregated filaments distinguishable by the naked eye (cf. Fig. 1B). In the USA, the CyanoTRACKER project is based on the use of a smartphone application by volunteers to

report algae blooms in their neighborhood pond and/or lake [12]. In the same way, citizen science programs based on the collection of water samples have been implemented for the monitoring of lake water quality and the survey of blooms in the province of Saskatchewan (Canada) [13] and in numerous lakes located in the state of Michigan (USA) [14].

Most of these projects take place in developed countries, fewer citizen programs being available in developing countries [15]. On the African continent, for example, a participatory local observatory has been implemented in Tanzania aiming at collecting and sharing data related to water, rainfall, fisheries, agriculture and food behavior between various stakeholders in water and land management [16]. In South Africa, Nare *et al.* [17] described the difficulties of community involvement and participation in water quality and management in a catchment area due to the inadequate institutional framework. Finally, a good relationship was found between the citizen monitoring of algal blooms and standard laboratory measurements in urban lakes and ponds of three cities located in Brazil and China [18]. These programs highlight the important benefits of this collaboration in terms of both knowledge production, opportunities for democratizing knowledge and expertise under the context of growing environmental and health risks [19].

In the present paper, we describe a citizen monitoring program for cyanobacterial blooms based on the use of a smartphone application that was implemented in three villages located around the Lagoon Aghien in the Ivory Coast. This study was performed in the framework of a collaborative project between three African countries (Ivory Coast, Senegal and Uganda) and France (WaSAf project, https://humbert19.wixsite.com/wasaf). The three main goals of this participatory approach were to evaluate (i) the potentials and constraints for the mobilization of the local population in an African country for the visual survey of cyanobacteria in aquatic ecosystems, (ii) the efficiency of citizen monitoring of cyanobacteria by comparing citizen data with data obtained using a classical sampling approach performed by the Pasteur

Institute of Abidjan and (iii) the contribution of citizen monitoring to awareness in the neighboring populations regarding the cause and health consequences of cyanobacterial blooms.

Material & methods

Study site

Lagoon Aghien is a freshwater ecosystem located in the northeastern part of the city of Abidjan. This lagoon covers a surface area of 19.5 km^2 with a perimeter of 40.72 km and a volume of 25 km^3 [20]. Lagoon Aghien belongs to a network of lagoons surrounding the city of Abidjan, with the particularity of being the only one containing freshwater. This lagoon is considered a potential water resource for the city of Abidjan in complement to the water supply currently provided by groundwater extraction. Approximately 12,000 people live in the immediate proximity of the lagoon, mostly distributed in eight villages located on its shoreline (Fig 1A). The lagoon is used by local populations for various domestic (bathing, washing, cooking, waste management, etc.) and economic (fishing, agriculture, etc.) practices that have an impact and are impacted by the water quality of the lagoon. Due to growing human populations and activities, not only in the immediate environment of the lagoon but also in its whole watershed, coupled with a dramatic lack of sanitation infrastructure and changes occurring in the land use occupation, Lagoon Aghien is now eutrophicated, and cyanobacterial blooms are frequently observed (Fig 1B). Despite the crucial importance of the lagoon for drinking water production, no institutional monitoring of water quality is currently performed.

The villages Débarcadère, Akandjé and Aghien-Télégraphe were chosen for the implementation of citizen monitoring because of (i) their geographical location enabling satisfactory coverage of the lagoon (Fig 1A) and (ii) the local authorities of the three villages agreed to participate in this experiment.

Fig 1. A. Map of the Lagoon Aghien with the locations of the three villages in which citizen monitoring of cyanobacteria was implemented (Débarcadère, Akandjé, Aghien-Télégraphe). B. Photo of a *Microcystis* sp. (cyanobacteria) bloom in a bay of the Lagoon Aghien.

Stars S1-S6 indicate the sampling points in the monitoring performed by the Pasteur Institute of Abidjan

Ethic statement

This study was not submitted for approval by an institutional review board (ethics committee) because there is currently no committee in Ivory Coast for environmental studies. But as described latter in the paper, we organized in each of the three villages concerned by this study, a meeting with the chiefdoms in order to present the project and to ask them for their agreement before to begin to work. In addition we asked also to the chiefdoms to designate the sentinels (referent people) in each village who have provided most of the data. Finally, our smartphone application entailed the possibility to collect anonymous reports.

Social engineering for participative water quality monitoring

In agreement with the recommendations of Sturm et al. [21], variou[illegible]tions were performed with local populations with the goal to prepare, conduct and ensure the follow-up and finally report the results of the participatory monitoring in the three villages (Fig 2)

Fig 2. Global overview of the different stages in our approach on the implementation of citizen monitoring of cyanobacteria in the three villages of the Lagoon Aghien

As shown by this figure, the participatory monitoring benefited from sociological investigations conducted within the framework of the WaSAf program in the riparian villages to better understand the local practices and uses related to the lagoon and the local social organization. Meetings and interviews with local community leaders and chiefdom and with inhabitants of the three villages made it possible to better understand their concerns regarding the ecological status of the lagoon and the local interest in cyanobacteria monitoring. After choosing the three relevant villages, focus groups were also conducted with the more specific objective of identifying the opportunities and constraints for implementing participatory monitoring. In each of the three villages, three different focus groups were organized with homogenous social categories according to gender and age (women, men and youths), including between 7 and 12 participants. These focus groups permitted us to find a common language between scientists and inhabitants, to better define the questionnaire in the mobile application and to generate enthusiasm among the participants to collaborate.

Focus groups were also used to present and test mobile applications on mobile phones with the volunteers and explain the use of the ap[illegible]ation (S1A Fig). Flyers explaining this use were distributed to participants to encourage large participation. To ensure regular data on the water quality of the lagoon, one person, designated as a sentinel, was chosen in each village by the traditional chiefdoms. In the first village, the sentinel was a school teacher, while in the second village, he was the representative of the young people and in the third village, the chief of the village was chosen. Each sentinel w[illegible]quipped with a mobile phone provided by the WaSAf program and was encouraged to take at least one picture per week of the lagoon, always in the same location (chosen together with the research team). The sentinel was also in charge of facilitating the participation of other members of the community willing to participate in the monitoring (helping them to install and use the application, explain the interest in the monitoring, etc[illegible]

S1 Fig. Presentation of the smartphone application (A) and restitution workshop (B) in Débarcadère village (Ivory Coast, shore of Lagoon Aghien).

To maintain the interest of participants and encourage them to continue contributing, we sent SMS messages of thanks to the contributors having registered their phone number in the smartphone application twice during the experiment. In accordance with the project team commitments and to facilitate data appropriation in the villages, two restitution events (based on a poster presentation – S1B Fig) were organized in each village at the end of each year of monitoring; prospectus (Lagoon Health Bulletin) were also distributed in schools and villages. These events permitted us to present the comparison between data provided by citizen participation and those obtained from the water laboratory analyses performed by the Pasteur Institute of Abidjan. Finally, at the end of the first two years, we also conducted qualitative interviews with the three sentinels and other volunteers who participated in monitoring to evaluate the effectiveness and difficulties related to participatory monitoring, as well as their appropriation of the monitoring experience.

The participatory monitoring was organized in two periods: (i) during the first period (from October 2017 to August 2018), the sentinels were asked to provide at least one report per week, and (ii) during the second period (from September 2018 to August 2019), the sentinels were asked to collect and send reports only when something interesting concerning the water quality in general and/or the presence of a cyanobacterial bloom was detected.

Smartphone application

The generalized access to mobile phone services endowed with geographic information systems (GIS) creates new opportunities for data collection, access and sharing all over the world [22-24]. According to data from the World Bank [25], access to cellular networks has reached 90 % in numerous African countries, while access to drinking water is only approximately 86% and access to sanitation is close to 60%. Consequently, the use of a smartphone application seemed to be a good tool for involving local communities in participatory monitoring. We adapted an open source smartphone application that is part of the Epicollect5 data-gathering platform (https://five.epicollect.net/) to the specific needs of the monitoring. This solution allowed the generation of project-dedicated questionnaires and the collection of georeferenced data that could be displayed through a web application in the form of maps, tables and charts. For each entry in the WaSAf project (S2 Fig), users completed a short questionnaire asking for the presence of (i) a green water color and a visible mass of algae suspended in the water, (ii) traces of fuel contamination, (iii) dead fish, (iv) garbage accumulation and/or if there was nothing to report or if they wanted to add other observations. For each questionnaire, users were asked to update their geo-location and to add a picture if they wished. Users could also provide their phone number. This optional information could be used by the project team to reach the users to request more data or provide feedback (two SMS campaigns were sent). At the end of each questionnaire, users recorded their data and sent them to the website if a data connection was available. If there was no internet connection, the recorded data could be sent later. As soon as a questionnaire was sent, the data were available online. All the data collected in the framework of this project are available at the project website: https://five.epicollect.net/project/wasaf.

S2 Fig. Screenshots of the Epicollect-5 smartphone application with the WaSAf project.

Characteristics of the monitoring of cyanobacteria performed by the Pasteur Institute of Abidjan

The phytoplankton community was monitored for seven months by the Pasteur Institute of Abidjan from October 2017 to April 2018 at six sampling stations (S1-S6) (Fig 1A). Integrated water sampling was performed in the first meter of the water column using an integrated sampler according to the technique used by Laplace-Treyture *et al*. [26]. The counting of cyanobacteria was performed on an inverted microscope (Nikon Eclipse TS100) using the Utermöhl method, according to the AFNOR 15204 standard. A minimum of 30 randomly selected fields was counted to attain at least 500 counting units (cells, colonies or filaments/trichomes).

The results were then expressed as the number of cells per milliliter. In the case of the colonial cyanobacteria *Microcystis* sp., two categories of colonies were considered regarding their diameter (< or > 200 µm). The mean number of cells per category was estimated under an upright microscope by counting the number of cells in 30 colonies per category after they were gently crushed between slides and coverslips. For the filamentous cyanobacteria *Aphanizomenon*, *Cylindrospermopsis*, *Limnothrix*, *Lyngbia*, *Oscillatoria*, *Planktothrix* and *Pseudanabaena*, the counting considered filament lengths of 100 µm. The mean number of cells per 100 µm of filament was estimated under an upright microscope for 30 filaments for each genus.

The mean cell biovolumes were calculated for the cyanobacteria genera that were most abundant in Lagoon Aghien (*Microcystis*, *Cylindrospermopsis*, *Limnothrix*, *Oscillatoria*, *Dolichospermum*) from measurements performed on 30 cells per genus. For each of the other genera, we used standard cell values defined in the HELCOM PEG Biovolume reports (http://ices.dk/marine-data/vocabularies/Pages/default.aspx).

Results

Overall presentation of the evolution of the number and locations of collected reports during the study period

Since the beginning of participatory monitoring, more than 660 questionnaires (January 2020) have been recorded in the WaSAf project hosted by the Epicollect5 platform (see https://five.epicollect.net/project/wasaf). During the first period of the study, an average of 44 completed questionnaires was collected each month, while during the second period, an average of 13 completed questionnaires were collected monthly (Fig 3). This difference in the number of collected questionnaires during the two periods was expected since we changed the recommendation for the sentinels from sending a picture at least once a week (regardless of whether cyanobacteria were observed) to sending pictures only if cyanobacteria or other degradation signs were observed.

Fig 3. Evolution of the number of completed questionnaires loaded on the WaSAf project Epicollect5 platform. During the first period, the sentinels were asked to complete at least one questionnaire per week with one picture of the lagoon. During the second period, sentinels were advised to send pictures only if cyanobacteria or other worrying phenomena were observed. Dashed line: Monthly average of collected questionnaires during each period

As shown in Fig 4A, the geographical origins of the data collected during this study were well distributed around Lagoon Aghien. Data were not only collected near the three villages. Many data points were collected in the western part of the lagoon by the sentinel from the Débarcadère village (Fig 4B). This sentinel performed visual monitoring at four points in this part of the lagoon. Finally, it also appears on the map that some of the data were not located

on the shoreline of the lagoon, although this phenomenon was probably due to the inaccuracy or misuse of the GPS of the smartphone.

Fig 4. Geographical distribution of the data collected by citizens around the Lagoon Aghien (A). Detail on the citizen monitoring performed by the sentinel from Débarcadère village (B)

Data indicating the potential presence of cyanobacterial blooms

The potential presence of cyanobacterial blooms in the lagoon was reported in the smartphone application by the presence of a green water color and visible algae in the wa[illegible] As shown in Fig 5, cyanobacteria blooms were reported mainly during four periods. The first peak of reports occurred in November 2017, followed by a small peak in February 2018, a third peak in May-June 2018 and finally a fourth large peak from May to September 2019.

Fig 5. Evolution of the nu[illegible]r of reports made by citizens on the potential presence of cyanobacteria in the Lagoon Aghien (green water color and/or presence of visible colonies)

The geographical locations of the potential algal blooms during the three periods were not the same. For the first algal bloom peak (November 2017), almost all reports were from the Débarcadère village, while for the two other peaks (May-June 2018 and May-September 2019), almost all reports of algal blooms were from the Aghien-Télégraphe village.

Comparison of the data provided by the participatory monitoring with those from the Pasteur Institute

Two increases in cyanobacterial biovolumes were observed during the monitoring performed by the Pasteur Institute from October 2017 to April 2018. The first increase was recorded in

November 2017[illegible]d the second one was recorded in February 2018 (Fi[illegible]. These data also highlighted that large variations occurred in these cyanobacterial biovolumes depending on the sampling stations, with the largest biovolumes being recorded at Station 4 and the lowest at Station 6. Finally, as shown in Fig 6, good congruence was found when comparing the data provided by the Institute Pasteur and those provided by citizen monitoring for the same period[illegible] suggesting th[illegible]pproach has the potential to contribute to the monitoring of cyanobacteria in Africa.

Fig 6. Comparison of the data of cyanobacterial biovolumes at six sampling stations of the Lagoon Aghien collected by the Pasteur Institute and the number of reports on the potential presence of cyanobacteria (green water color and presence of visible algae in the water) made by citizens. CM = Citizen monitoring. S1 to S6 = Sampling stations 1 to 6 for the biovolume estimations of cyanobacteria

Local understanding of the issues of water quality and cyanobacteria in the lagoon

Lagoon Aghien is a very important resource for riparian communities, as it is used for multiple domestic activities (cloth washing, bathing, dishware washing and sometimes cooking) and economic activities (fishing, irrigation, transports, etc.). Consequently, the lagoon has considerable value for the inhabitants who have mainly a good opinion about the quality of the lagoon (Fig 7[illegible]Almost 90% of the interviewed people from the Akandjé village and 56% from Aghien-Télégraphe told us that the lagoon exhibits “good and very good quality”. In the Débarcadère village, the quality perception was more negative (72% of interviewed people think that the lagoon is in a bad or very bad state[illegible]cause the village is located between two tributaries that collect urban runoff from Abidjan neighborhoods. When speaking about the evolution of the lagoon quality over time, local people spontaneously refer to the evolution of

fishing captures (a decrease over the last 20 years according to their perception). However, when asked to explicitly explain the way they perceive the water quality of the lagoon at a given moment and the eventual changes in this quality, the first criterion that is mentioned is the changes in the water color (Fig 7B). The importance of water color for the assessment of water quality by inhabitants was seen by us as a positive signal for the implementation of participative monitoring of cyanobacteria blooms.

Fig 7. Perception of the water quality in Lagoon Aghien by residents of the three villages (Aghien-Télégraphe, Akandjé, Débarcadère). Respondents were asked (A) how they evaluate the quality of the lagoon using a four-point scale (very good, good, bad, very bad) and (B) which criteria they use for the assessment of the water quality.

The focus groups and qualitative interviews also provided interesting material to understand the precise implications that are associated with various color changes. Indeed, the participants in the focus groups indicated two main water changes: (i) brown water (muddy, reddish) observed mainly during the rainy seasons (from May to July and from September to November), and (ii) green water observed before and/or after the rainy seasons. The brown color was (mainly) associated with “dirty water” coming from the watershed during the rainy periods. During these periods, inhabitants take precautions, and the lagoon is less used for domestic activities to avoid water-related diseases such as diarrhea. The green color of the lagoon was not associated with any sanitary or environmental risks. In contrast, some participants noticed a coincidence between green water color and fish abundance: "*When the water turns green, it smells such as fish, it is fish poop, it is to tell the fishermen that there is a lot of fish and that they can go out fishing*” (Focus group women, Akandjé, Nov. 2017). Only some of the villagers noticed that bathing activities during these green water periods could

generate skin and eye irritation. Consequently, no or very few precautions related to domestic uses of water are taken during cyanobacteria blooms. Finally, many participants of the focus groups made a link between the brown color (the runoff pollution) and the green color (eutrophication), with the first color often preceding the second.

[illegible] initial focus groups and the restitution events represented important opportunities for explaining the reasons why researchers and members of the Pasteur Institute were so interested in the monitoring of the “green spots”, as local people called them. On the one hand, local observations are relevant (the phytoplankton biomass production fosters the fish populations), but it has to be stated and explained that a massive development of cyanobacteria also represents a sanitary risk for human activities and can be considered a good indicator of the overall evolution of the water quality in the lagoon. On the second hand, such moments of exchange are necessary to motivate the local communities and raise local concern about cyanobacterial blooms, which were previously considered as a natural phenomenon.

Finally, the restitution events were “arenas” for the communities to discuss the causes and consequences of the degradation of the water quality in the lagoon with scientists. Local chiefdoms were very willing to discuss (i) the contribution to the pollution of the lagoon by populations living close to the lagoon compared to that originating from the rest of the watershed and (ii) the actions and programs that could be implemented to protect/restore the water quality in the lagoon.

Discussion

Although the participation of nonscientists in environmental monitoring is not a recent practice, the phenomenon has undergone spectacular development over the last twenty years, facilitated by new communication technologies and mobile computing applications. This first citizen project based on the use of a smartphone application for the monitoring of cyanobacterial

blooms in Africa shows that there opportunity for such approaches for both (i) the improvement of the monitoring of cyanobacteria in addition to the monitoring performed by relevant institutions and (ii) the improvement of the awareness and social learnings for the local communities concerning the health risks associated with cyanobacteria and more globally about the degradation of the water quality.

Concerning the improvement of institutional monitoring, as mentioned in the introduction, it is well known that accurate monitoring of cyanobacterial blooms is very costly. This financial cost is a major constraint for the implementation of long-term monitoring in developing countries because of the difficulty of securing long-term funding in these countries. The best illustration of this issue was that the monthly monitoring performed by the Pasteur Institute of Abidjan, at the same time as the participatory monitoring implementation, was stopped after seven months because of the lack of financial resources [illegible] this first pilot study showed the findings provided by the participatory monitoring were congruent with those provided by the Pasteur Institute of Abidjan, it is interesting to raise the issue of the place of participatory monitoring in an overall monitoring system for the Lagoon Aghien. First, citizen monitoring could be an early warning system for cyanobacterial blooms, which could trigger water samples and analyses. This system could significantly (i) improve the efficiency of institutional monitoring based, for example, on a monthly sampling frequency in a limited number of sampling points and (ii) reduce the cost of institutional monitoring if these two approaches are well coordinated. Second, citizen monitoring could also permit improved basic knowledge of the dynamics and toxicity of cyanobacterial blooms and, more globally, the knowledge of water quality, if it is coupled, for example, with water sampling by untrained and equipped volunteers, as has been done in other participatory projects [13].

Concerning the local awareness and social learnings about cyanobacteria, the focus groups showed that local populations living around the Lagoon Aghien could spontaneously

identify visible pollution, as well as changes in physical and biological parameters (such as the water color, macrophytes and algal blooms). At the same time, these focus groups also revealed the low awareness of these populations concerning the health risks associated with cyanobacterial blooms. During the preparation and feedback meetings organized in the three villages, there were also many discussions on the causes of cyanobacterial blooms in freshwater ecosystems. The issue of eutrophication was well understood by most of the participants, with interesting debates on the relative contribution of nutrient discharge into the lagoon from local pollution *versus* “distant pollution” from the other parts of the Abidjan district located in the watershed. These discussions, for example, led the chiefdom representatives of one village to prohibit defecation in the lagoon by the inhabitants of this village and asked for sanitation investments from the regional authorities. Finally, it appears that the social benefits associated with the citizen monitoring approach are both direct (new learning and understanding of water ecosystem functioning and the eutrophication issue and its consequences to cyanobacterial blooms; new capacities to identify pollution sources and sanitary and ecological risks, etc.) and indirect (their appropriation and translation into conservation actions, etc.).

Several limits were also identified during this first approach on the monitoring of cyanobacteria by citizens in the Ivory Coast. These limits can be qualified as (i) data accuracy - or technical limits; (ii) participatory limits; and (iii) institutional limits.

The first technical limit is related to the accuracy of the produced data, which is affected by the approximate geolocations of several reports, which was probably due to the misuse or poor quality of the GPS of some smartphones. However, it is likely that the rapid enhancement of smartphone quality will reduce this problem in future years. In the same way, the poor quality of some of the pictures associated with the questionnaires did not permit visual confirmation of the presence of "green al[illegible] indicated in the questionnaire. Again, it is likely that the rapid

enhancement of camera quality in smartphones [27] will permit us to solve this problem in the future.

The second limit in the participatory monitoring during this experiment concerns the fact that despite a very important number of registered data, most data were sent by the local sentinels. It was not always possible to know who sent the reports because the name and the phone number were optionally requested; however, when the phone number was provided, it appeared that the sentinels of the three villages sent most of the reports. It is likely that some social/gender categories do not seem to contribute to citizen monitoring such as for example, women, although they are in close contact with the lagoon as they have multiple uses for it. However, from the interviews with the sentinels, it appeared that some villagers who observed changes in the water color asked the sentinels to create a report on the application. Thus, the role of sentinels was very important in the villages, which made the selection of sentinels very important. In our experiment, the best contributor as a sentinel was clearly the schoolteacher from the Débarcadère village, who self-planned to conduct multipoint monitoring. As noted by Requier *et al.* [28], the issue of long-term participation and the improvement of volunteer participation is crucial in developing countries due to the poverty of the local population and logistical constraints.

Finally, the institutional limit in our citizen monitoring approach concerns the integration of the data collected by the citizens within the framework of the institutional monitoring of the lagoon, which is actually missing. The two ministries in charge of the management of water resources and drinking water production have expressed great interest in this participatory approach but failed to see how the collected data could be integrated by their institutions. This result was not because participatory data were considered useless or not accurate enough but because the institutions do not devote the financial and human resources needed for long-term monitoring of freshwater resources. A great deal of awareness-raising

among these institutions still needs to be done to show how citizen empowerment might be integrated in the framework of the implementation of long-term monitoring of freshwater resources. In this goal, we are currently working with the different water institutions for the implementation of an observatory in the Lagoon Aghien, in which citizen monitoring would have a place as an early warning system for cyanobacterial blooms.

In conclusion, in addition to the design of a smartphone application and the collection of data on cyanobacterial blooms in an African freshwater ecosystem, our citizen monitoring project considered the social aspects of participatory science. These social factors are crucial because they are both a condition for effective participatory monitoring and a condition for maximizing the social utility of the procedure. From this perspective, participatory science is much more than recruitment of volunteers for data collection. The empowerment of knowledgeable citizens, the adoption of critical thinking skills related to the environment and the strengthening of science-society relationship are acknowledged as one crucial point of participatory science projects, even if these points are sometimes marginal in the design of participatory science projects [29, 30].

Acknowledgments

We are extremely grateful to the three sentinels (Mr. Blaise Lattho in Aghien-Télégraphe, Mr. Kouandjo Guy Rolland Adon in Akandjé, and Mr. Kameleman Siaka Issoufou in Débarcadère) and the inhabitants and chiefdoms of the three villages (Aghien-Télégraphe, Akandjé and Débarcadère) for their decisive contribution to this work. This work was supported by the French Facility for Global Environment (Fonds Français pour l'Environnement Mondial, FFEM) (research program WaSAf).

References

1. Welker M, Döhren von H, Täuscher H, Steinberg CEW, Erhard M. Toxic *Microcystis* in shallow lakes Müggelsee (Germany) - dynamics, distribution, diversity. Arch Hydrobiol 2003; 157: 227-248.
2. Cuypers Y, Vinçon-Leité B, Groleau A, Tassin B, Humbert JF. Impact of internal waves on the spatial distribution of *Planktothrix rubescens* (cyanobacteria) in an alpine lake. ISME J 2011; 5: 580-589.
3. Pobel D, Robin J, Humbert JF. Influence of sampling strategies on the monitoring of cyanobacteria in shallow lakes: Lessons from a case study in France. Water Res 2011; 45: 1005-1014.
4. Ahn CY, Joung SH, Park CS, Kim HS, Yoon BD, Oh HM. Comparison of sampling and analytical methods for monitoring of cyanobacteria-dominated surface waters. Hydrobiologia 2008; 596: 413-421.
5. Steffensen DA. Economic cost of cyanobacterial blooms. In: Hudnell H.K. (eds) Cyanobacterial harmful algal blooms: State of the science and research needs. Adv Exp Med Biol 2008; vol 619. Springer, New York, NY
6. Le Vu B, Vinçon-Leite B, Lemaire B, Bensoussan N, Calzas M, Drezen C, et al. 2011. High-frequency monitoring of phytoplankton dynamics within the European water framework directive: application to metalimnetic cyanobacteria. Biogeochemistry 2011; 106: 229-242.
7. Garneau MV, Posch T, Hitz G, Pomerleau F, Pradalier C., Siegwart R, et al. Short-term displacement of *Planktothrix rubescens* (cyanobacteria) in a pre-alpine lake observed using an autonomous sampling platform. Limnol Oceanogr 2013; 58: 1892-1906.

8. Hunter PD, Tyler AN, Willby NJ, Gilvear D.J. The spatial dynamics of vertical migration by *Microcystis aeruginosa* in a eutrophic shallow lake: A case study using high spatial resolution time-series airborne remote sensing. Limnol Oceanogr 2008: 53; 2391-2406.

9. Moore TS, James H, Churnside JH, Sullivan JM, Twardowski MS, Nayak AR, et al. Vertical distributions of blooming cyanobacteria populations in a freshwater lake from LIDAR observations. Remote Sens Env 2019; 225: 347-367.

10. Dube T, Mutanga O, Seutioali K, Adelabu S, Shoko C. Water quality monitoring in sub-Saharan African lakes: a review of remote sensing applications. Afr J Aquat Sci 2015; 40: 1-7.

11. Poisson AC, McCullough IM, Cheruvelil KS, Elliott KC, Latimore JA, Soranno PA. Quantifying the contribution of citizen science to broad-scale ecological databases. Front Ecol Environ 2020; 18: 19-26.

12. Scott MD, Ramaswamy L, Lawson V. CyanoTRACKER: A citizen science project for reporting harmful algal blooms. Conference: 2nd IEEE International Conference on Collaboration and Internet Computing (IEEE CIC) 2016; 391-397.

13. Bos JS, Nanayakkara L, Hulbert M, Finlay K. Citizen science for Saskatchewan lakes: a pilot project. Lake Reserv Manage 2019; 35; 77-89.

14. Sarnelle O, Morrison J, Kaul R, Horst G, Wandell H, Bednarz R. Citizen monitoring: Testing hypotheses about the interactive influences of eutrophication and mussel invasion on a cyanobacterial toxin in lakes. Water Res 2010; 44: 141-150.

15. Pocock MJO, Roy HE, August T, Kuria A, Barasa F, Bett J et al. Developing the global potential of citizen science: Assessing opportunities that benefit people, society and the environment in East Africa. J Appl Ecol 2019; 56: 274-281.

16. Duvail S, Paul JL, Hamerlynck O, Majule A, Nyingi WD, Mwakalinga A et al. 2017. Recherches participatives en Tanzanie : un observatoire local pour un dialogue autour de la gestion des territoires et de l’eau. Nat. Sci. Soci. 2017 ; 25 : 347-359.
17. Nare L, Odiyo JO, Francis J, Potgieter N. Framework for effective community participation in water quality management in Luvuvhu catchment of South Africa. Phys Chem Earth 2011; 36: 1063-1070.
18. Castilla EP, Cunha DGF, Lee FWF, Loiselle S, Ho KC, Hall C. Quantification of phytoplankton bloom dynamics by citizen scientists in urban and peri-urban environments. Environ Monit Assess 2015; 187: 690
19. Buytaert W, Zulkafli Z, Grainger S, Acosta L, Alemie TC, Bastiaensen J et al. Citizen science in hydrology and water resources: opportunities for knowledge generation, ecosystem service management, and sustainable development. Front Earth Sci 2014; 2: 51-71.
20. Koffi SE, Koffi TK, Perrin JL, Seguis L, Guilliod M, Goné DL et al. Hydrological and water quality assessment of the Aghien Lagoon hydrosystem (Abidjan, Côte d’Ivoire). Hydrolog Sci J 2019; 64: 1893-1908.
21. Sturm U, Schade S, Ceccaroni L, Gold M, Kyba CMK, Claramunt B et al. Defining principles for mobile apps and plaftorms development in citizen science. RIO 2018; e23394.
22. Silvertown J. A new dawn for citizen science. Trends Ecol Evol 2009; 24: 467-471.
23. Brovelli MA, Minghini M, Zamboni G. Public participation in GIS via mobile applications. ISPRS J Photogramm 2016; 114: 306-315.
24. Mazumdar S, Ceccaroni L, Piera J, Hölker F, Berre AJ, Arlinghaus R et al. Citizen science technologies and new opportunities for participation. In: Hecker, S., Haklay, M., Bowser,

A., Makuch, Z., Vogel, J. & Bonn, A. Citizen Science: Innovation in Open Science, Society and Policy. UCL Press, London. 2018; Chap 21: 303-320.

25. World Bank. Water Hackathon - Lesson learned. 2012; Available on https://www.un.org/waterforlifedecade/pdf/2012_world_bank_water_hackathon_lessons.pdf).
26. Laplace-Treyture C, Barbe J, Dutartre A, Druart JC, Rimet F, Anneville O. Protocole standardisé d'échantillonnage, de conservation, d'observation et de dénombrement du phytoplancton en plan d'eau pour la mise en œuvre de la DCE : version 3.3.1. Cemagref, 2009 ; 44 p.
27. Coccia M. Functionality development of product innovation: An empirical analysis of the technological trajectories of smartphone. J Econ Libr 2018; 5:241-258.
28. Requier F, Andersson GKS, Oddi FJ, Garibaldi LA. Citizen science in developing countries: how to improve volunteer participation. Front Ecol Environ 2020; 18: 101-108.
29. Dickinson JL, Sirk J, Bonter D, Bonney R, Crain RL, Martin J et al. The current state of citizen science as a tool for ecological research and public engagement. Front Ecol Environ 2012; 10: 291-297.
30. Bonney R, Shirk JL, Phillips TB, Wiggins A, Ballard HL, Miller-Rushing A.J. et al. Next steps for citizen science. Science 2014; 343: 1436-1437.

Figure 1

Click here to access/download;Figure;Fig 1.pptx

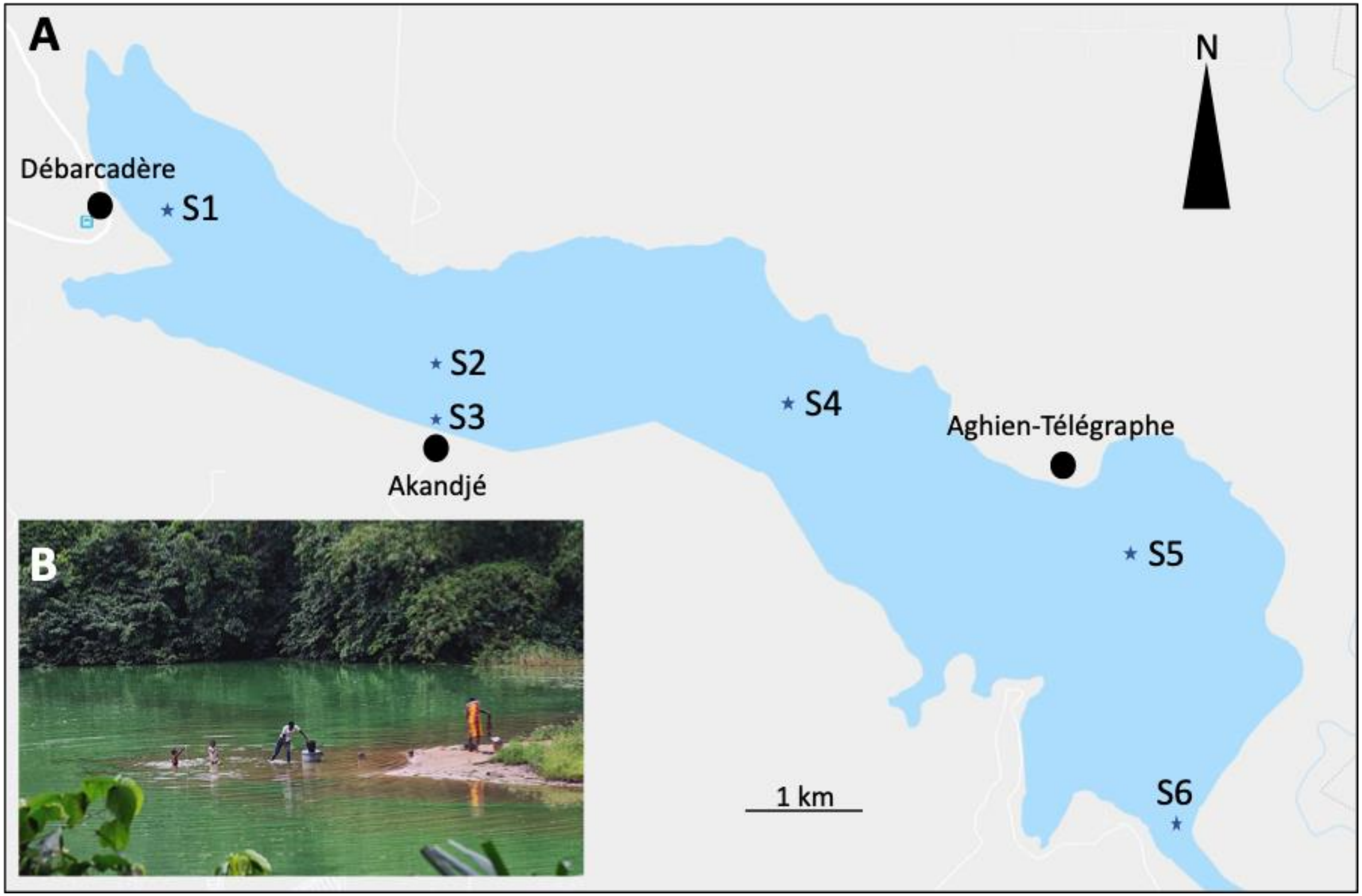

Figure 2

Click here to access/download;Figure;Fig 2.pptx

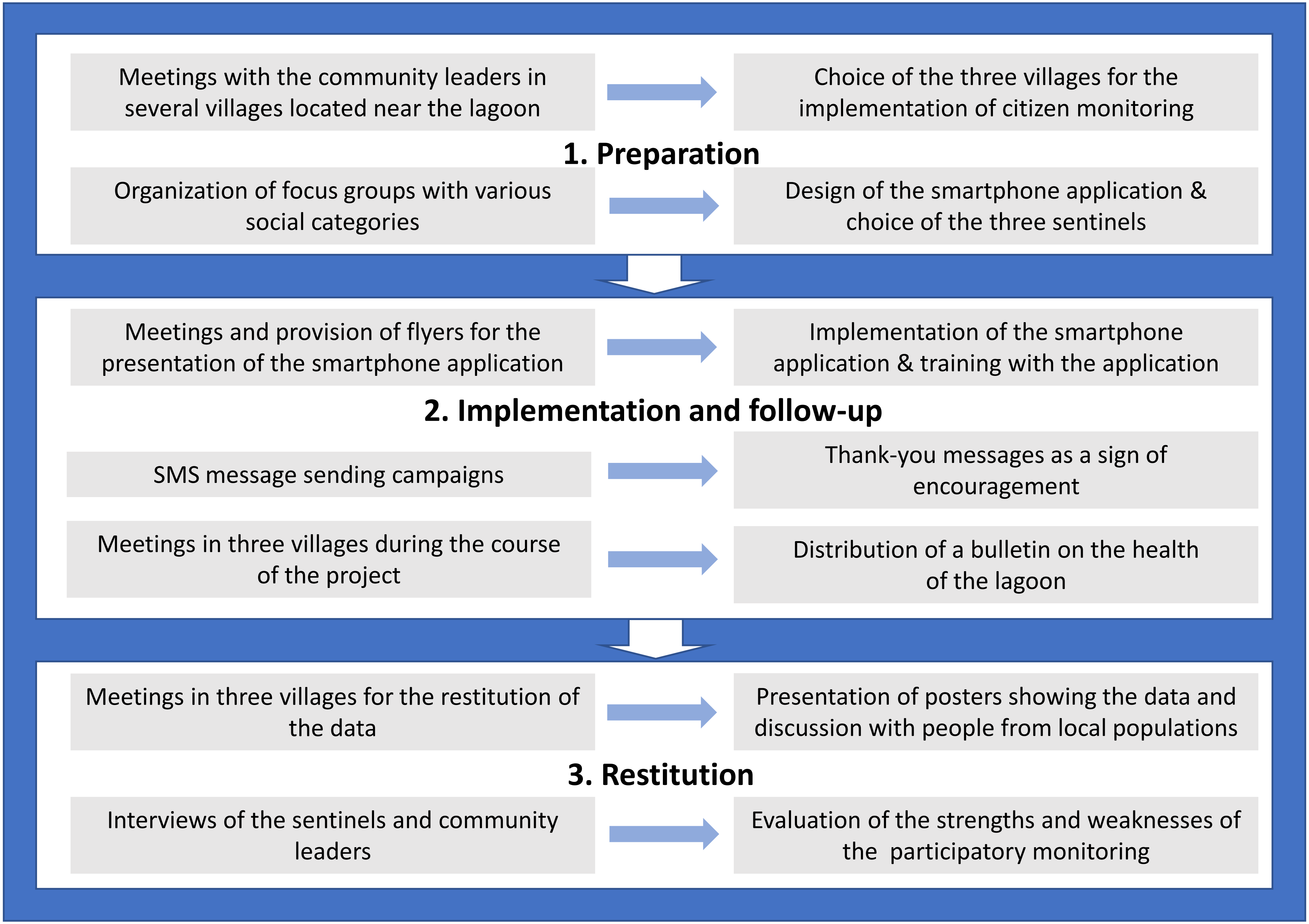

Figure 3

Click here to access/download;Figure;Fig 3.pptx

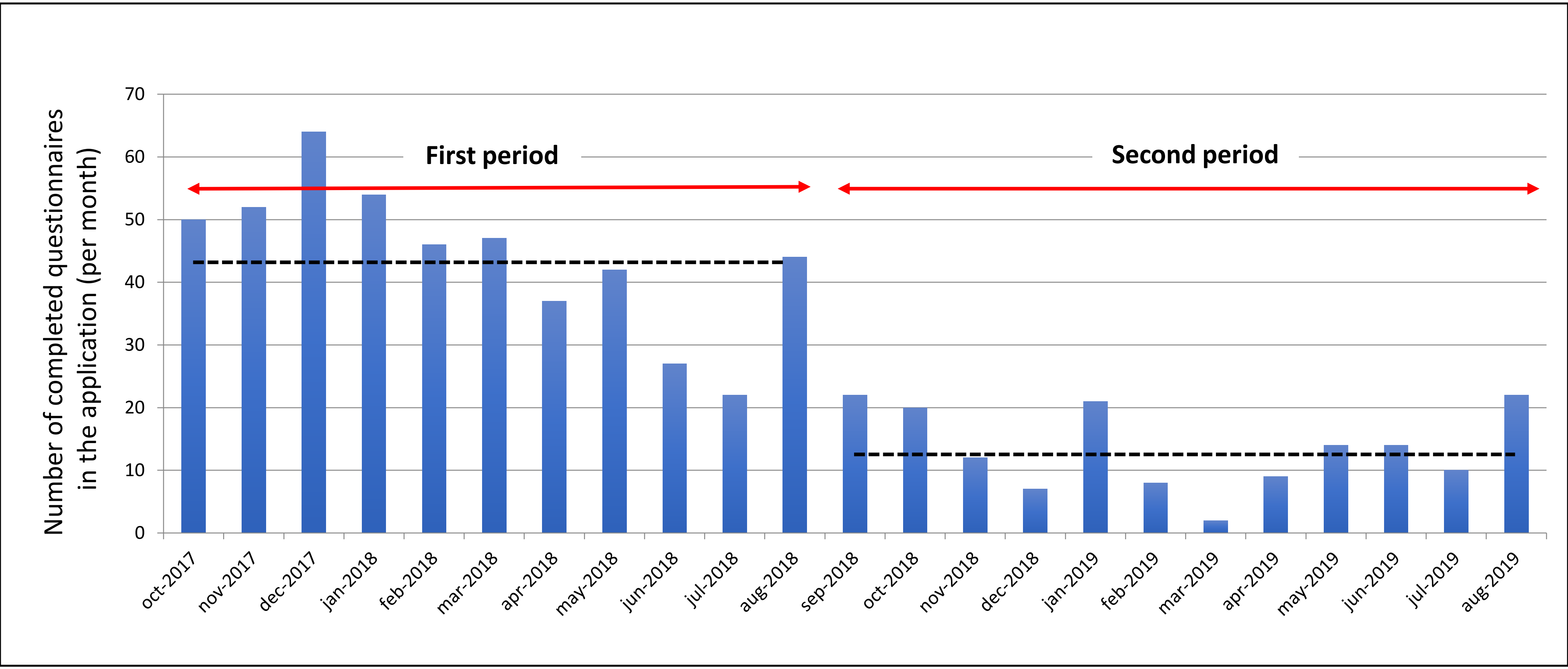

Figure 4

Click here to access/download;Figure;Fig 4.pptx

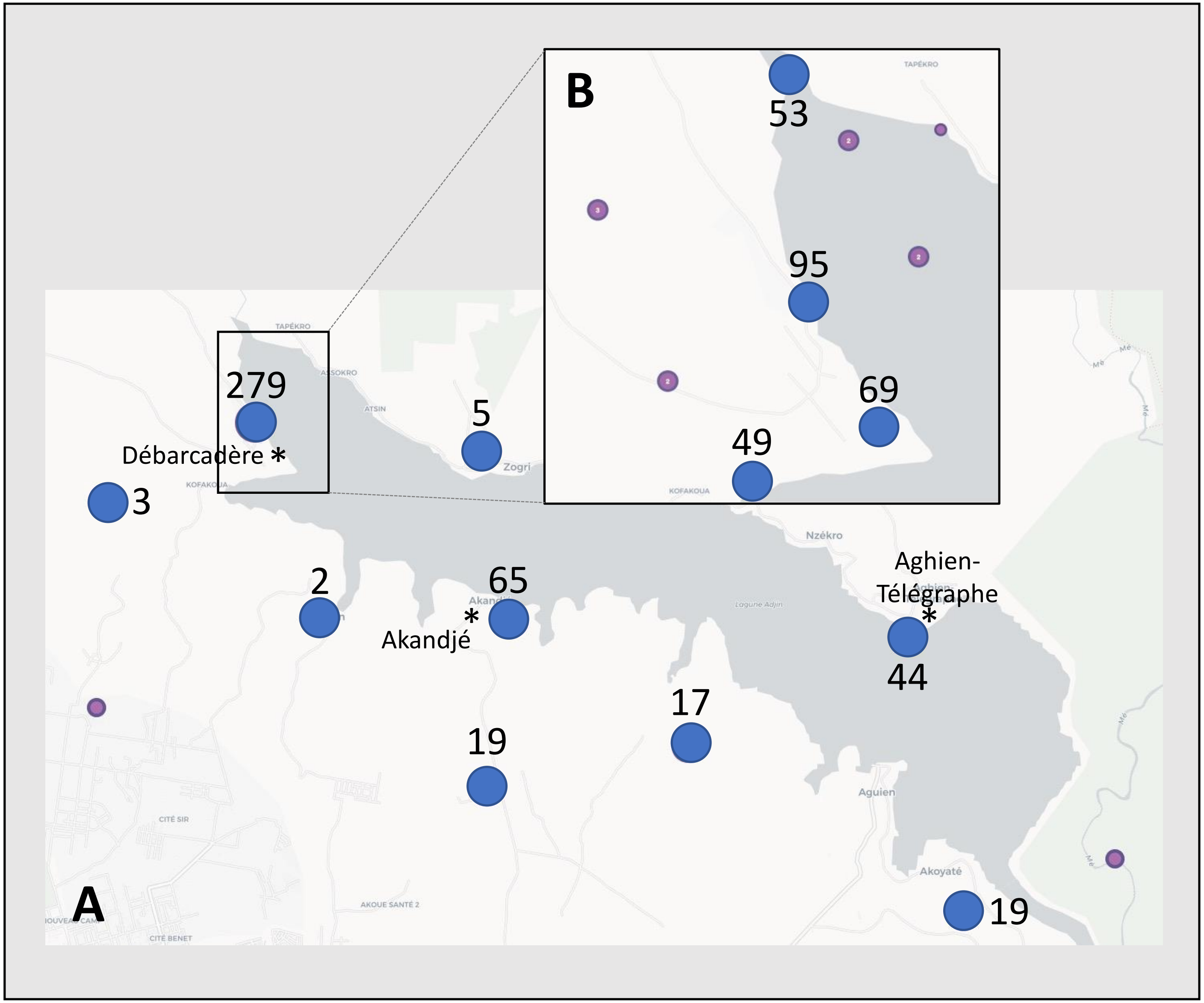

Figure 5

Click here to access/download;Figure;Fig 5.pptx

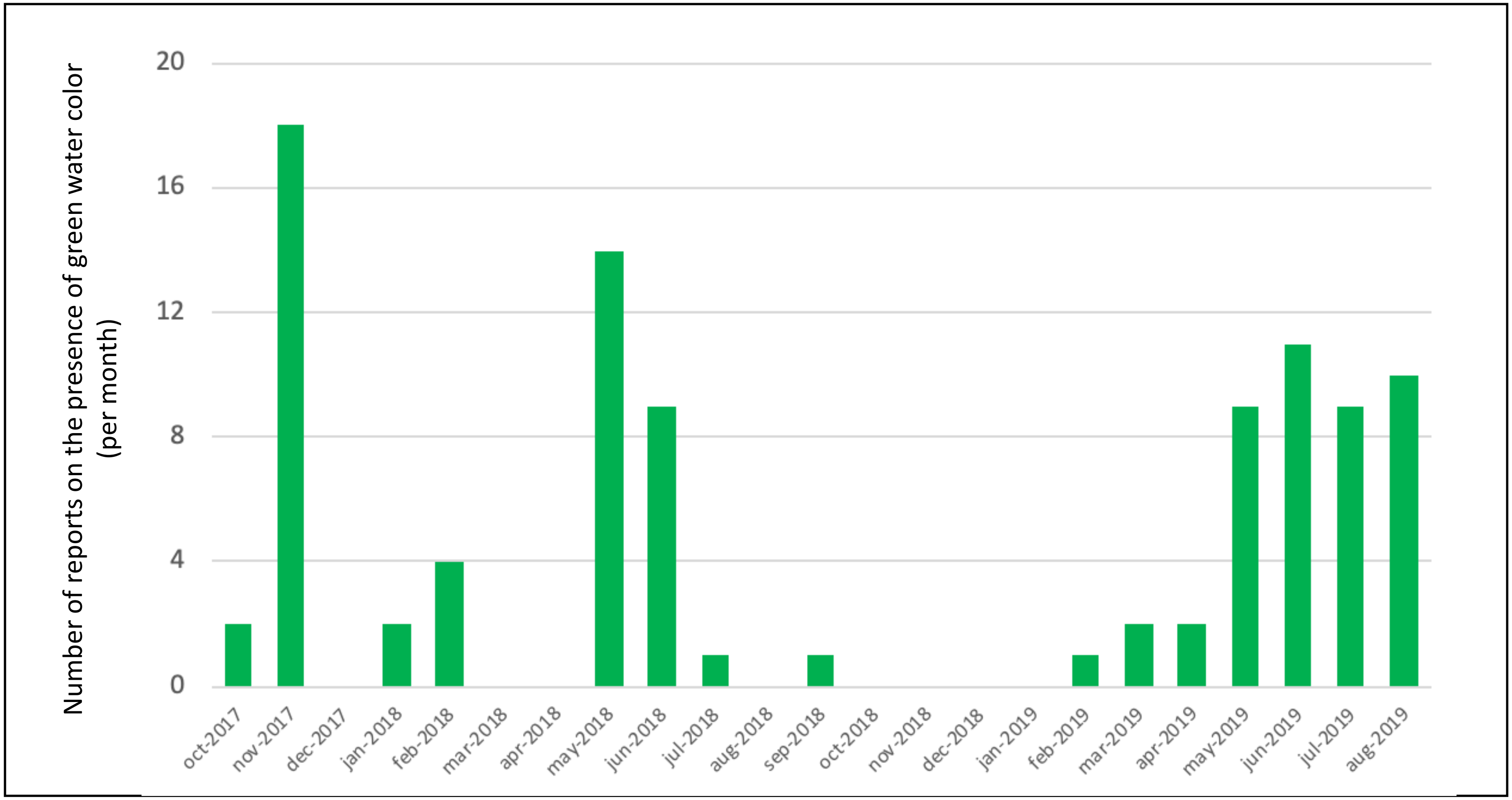

Figure 6

Click here to access/download;Figure;Fig 6.pptx

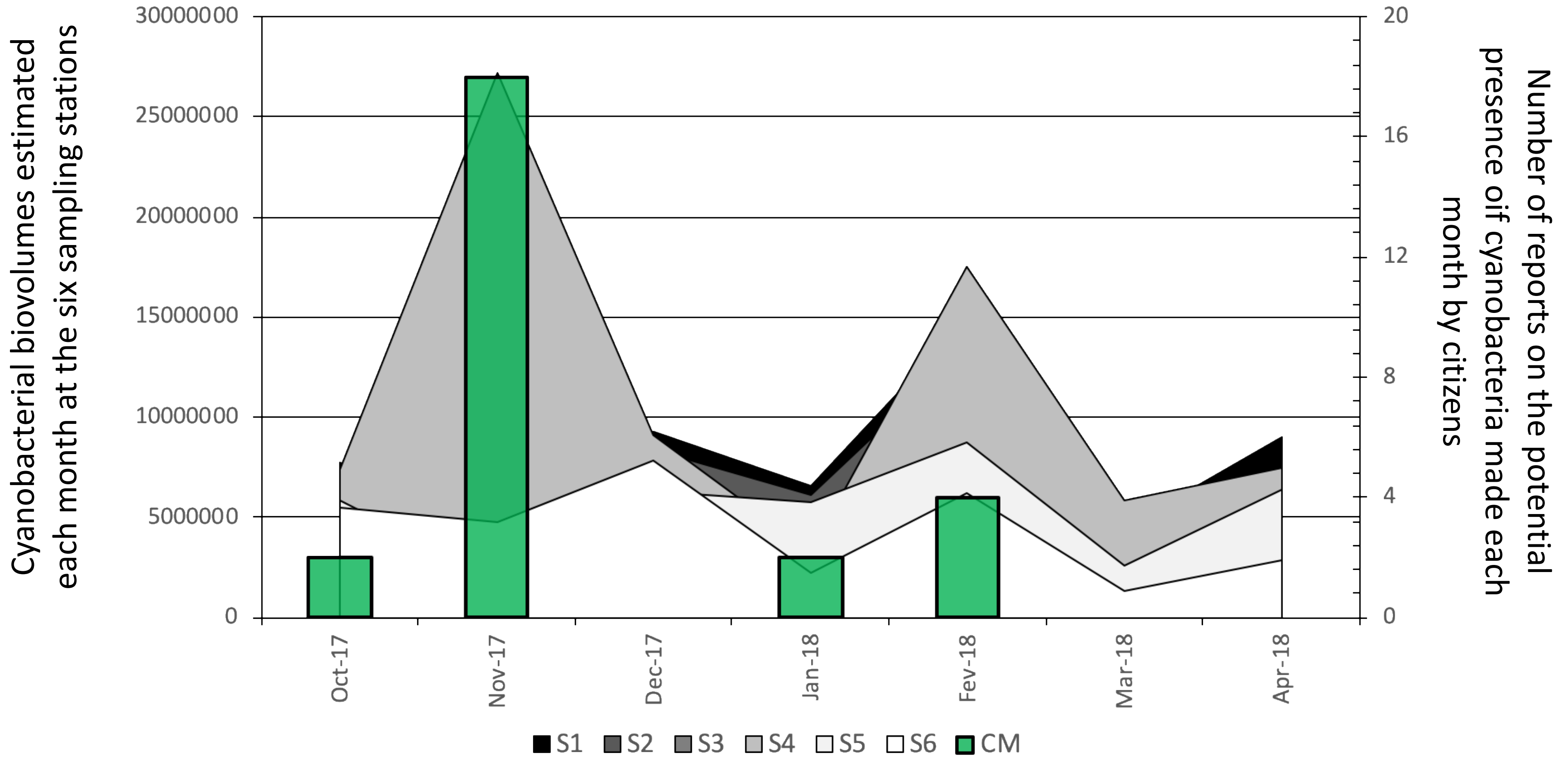

Figure 7

Click here to access/download;Figure;Fig 7.pptx

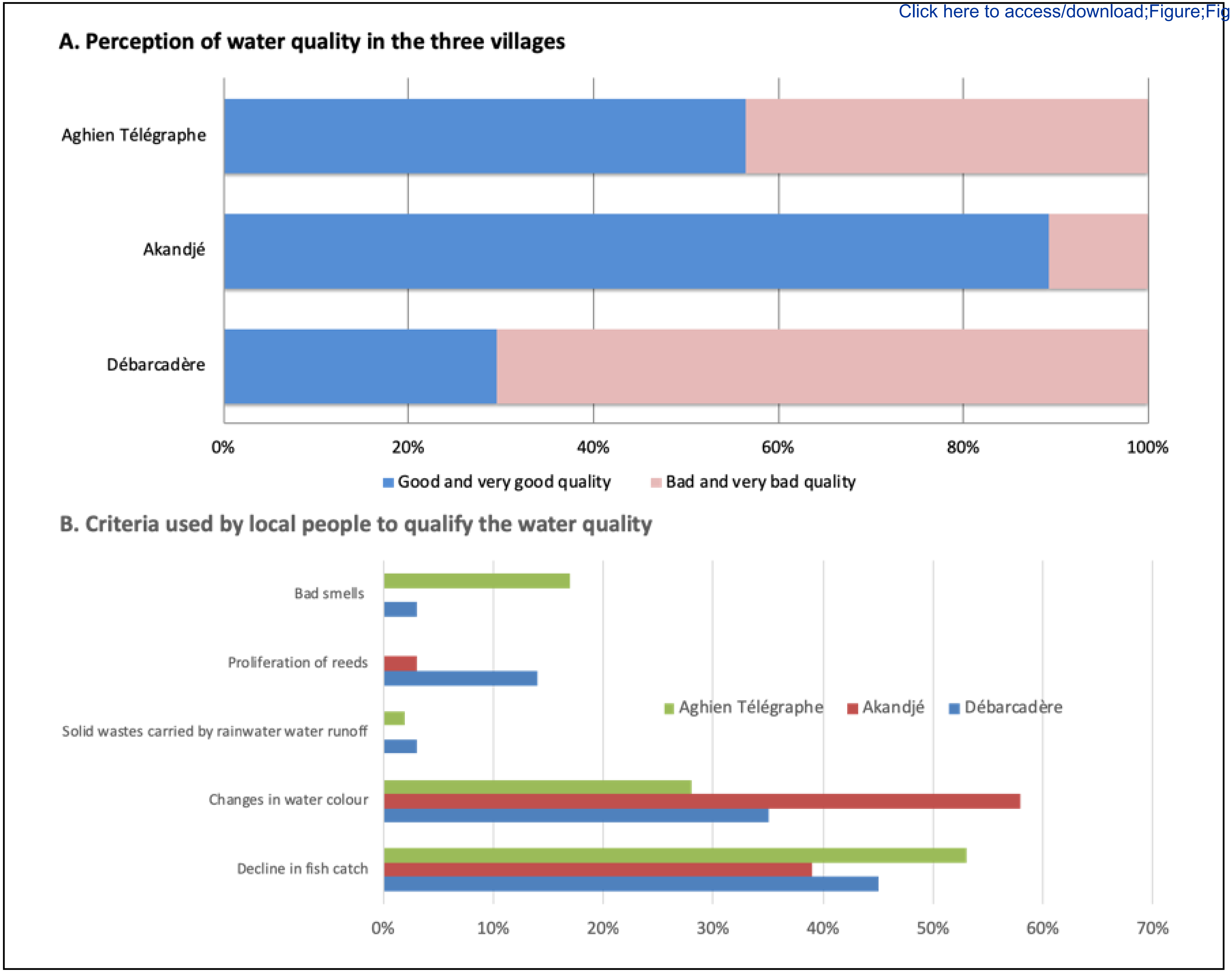

Sup Fig1

Click here to access/download
Supporting Information
S1 Fig.pptx

Sup Fig2

Click here to access/download
Supporting Information
S2 Fig.pptx
