# Supplementary material for: Can participatory approaches strengthen the monitoring of cyanobacterial blooms in developing countries? Results from a pilot study conducted in the Lagoon Aghien (Ivory Coast)
Source: PLoS One. 2020 Sep 24;15(9):e0238832. doi: 10.1371/journal.pone.0238832 (PMC7514105; doi:10.1371/journal.pone.0238832)
Supplement: Attachment — Submitted filename: PONE-D-20-09032_R1.pdf [file pone.0238832.s006.pdf]

PLOS ONE

Can participatory approaches strengthen the monitoring of cyanobacterial blooms in developing countries? Results from a pilot study conducted in the Lagoon Aghien (Ivory Coast)

--Manuscript Draft--

**Manuscript Number:**	PONE-D-20-09032R1
**Article Type:**	Research Article
**Full Title:**	Can participatory approaches strengthen the monitoring of cyanobacterial blooms in developing countries? Results from a pilot study conducted in the Lagoon Aghien (Ivory Coast)
**Short Title:**	Citizen monitoring of cyanobacteria in Africa
**Corresponding Author:**	Jean-François Humbert<br>INRA<br>Paris, UPMC FRANCE
**Keywords:**	Citizen monitoring; cyanobacteria; Lagoon Aghien; Ivory Coast
**Abstract:**	Monitoring of cyanobacteria in freshwater ecosystems is a complex task, which is time consuming and expensive due to the chaotic population dynamics and highly heterogeneous distribution of cyanobacteria populations in water bodies. The financial cost constitutes a strong limitation for the implementation of long-term monitoring programs in developing countries, particularly in Africa. The work presented here was performed in the framework of an international project addressing the sustainable monitoring and management of surface water resources used for the production of drinking water in three African countries. We tested the potential of a citizen approach for monitoring cyanobacterial blooms, which are a growing threat to the drinking water supply. This pilot study was designed, implemented and evaluated in close interaction with the Pasteur Institute of the Ivory Coast and with the populations of three villages located on the shoreline of a freshwater lagoon located near Abidjan city. Based on the use of a smartphone application, the citizens of the three villages were invited to report water color changes, as these changes could reflect cyanobacteria proliferations. A two-year experimentation period has shown that it is possible to mobilize the local populations to monitor cyanobacterial blooms. The data collected by citizens were consistent with the data obtained by a classical monitoring of cyanobacteria performed over seven months, but it appeared that new approaches were needed to validate the citizen data. This participatory approach also provided great improvements to the understanding and awareness of local populations regarding water quality and cyanobacterial bloom issues. Finally, we discuss some of the difficulties and limitations of our participatory monitoring approach that should be considered by further implementations. Despite these difficulties, our work suggests that citizen monitoring is a promising approach that may complement the classical approach to sustainable monitoring of cyanobacteria in developing countries.
**Order of Authors:**	Veronica Mitroi
	Kouadio Chrislain Ahi
	Pierre Yves Bulot
	Fulbert Tra
	José Frédéric Deroubaix
	Mathias Koffi Ahoutou
	Catherine Quiblier
	Mariatou Koné
	Julien Coulibaly Kalpy
	Jean-François Humbert
**Response to Reviewers:**	We have removed the former Fig S1

Powered by Editorial Manager® and ProduXion Manager® from Aries Systems Corporation

Additional Information:	
**Question**	**Response**
**Financial Disclosure**<br><br>Enter a financial disclosure statement that describes the sources of funding for the work included in this submission. Review the submission guidelines for detailed requirements. View published research articles from *PLOS ONE* for specific examples.<br><br>This statement is required for submission and **will appear in the published article** if the submission is accepted. Please make sure it is accurate.<br><br>**Unfunded studies**<br>Enter: *The author(s) received no specific funding for this work.*<br><br>**Funded studies**<br>Enter a statement with the following details:<br>• Initials of the authors who received each award<br>• Grant numbers awarded to each author<br>• The full name of each funder<br>• URL of each funder website<br>• Did the sponsors or funders play any role in the study design, data collection and analysis, decision to publish, or preparation of the manuscript?<br>• **NO** - Include this sentence at the end of your statement: *The funders had no role in study design, data collection and analysis, decision to publish, or preparation of the manuscript.*<br>• **YES** - Specify the role(s) played.<br><br>* typeset	VM, French Facility for Global Environment, https://www.ffem.fr/en<br>KCA, French Facility for Global Environment, https://www.ffem.fr/en<br>MKA, French Facility for Global Environment, https://www.ffem.fr/en<br><br>NO
**Competing Interests**<br><br>Use the instructions below to enter a competing interest statement for this submission. On behalf of all authors, disclose any competing interests that could be perceived to bias this work—acknowledging all financial support and any other relevant financial or non-financial competing interests.	NO authors have competing interests

This statement **will appear in the published article** if the submission is accepted. Please make sure it is accurate. View published research articles from *PLOS ONE* for specific examples.<br><br>**NO authors have competing interests**<br><br>Enter: *The authors have declared that no competing interests exist.*<br><br>**Authors with competing interests**<br><br>Enter competing interest details beginning with this statement:<br><br>*I have read the journal's policy and the authors of this manuscript have the following competing interests: [insert competing interests here]*<br><br>* typeset	
**Ethics Statement**<br><br>Enter an ethics statement for this submission. This statement is required if the study involved:<br><br>• Human participants<br>• Human specimens or tissue<br>• Vertebrate animals or cephalopods<br>• Vertebrate embryos or tissues<br>• Field research<br><br>Write "N/A" if the submission does not require an ethics statement.<br><br>General guidance is provided below. Consult the submission guidelines for detailed instructions. **Make sure that all information entered here is included in the Methods section of the manuscript.**	This study was not submitted for approval by an institutional review board (ethics committee) because there is currently no committee in Ivory Coast for environmental studies. But as described latter in the paper, we organized in each of the three villages concerned by this study, a meeting with the chiefdoms in order to present the project and to ask them for their agreement before to begin to work. In addition we asked also to the chiefdoms to designate the sentinels (referent people) in each village who have provided most of the data. Finally, our smartphone application entailed the possibility to collect anonymous reports.

**Format for specific study types**<br><br>**Human Subject Research (involving human participants and/or tissue)**<br>• Give the name of the institutional review board or ethics committee that approved the study<br>• Include the approval number and/or a statement indicating approval of this research<br>• Indicate the form of consent obtained (written/oral) or the reason that consent was not obtained (e.g. the data were analyzed anonymously)<br><br>**Animal Research (involving vertebrate animals, embryos or tissues)**<br>• Provide the name of the Institutional Animal Care and Use Committee (IACUC) or other relevant ethics board that reviewed the study protocol, and indicate whether they approved this research or granted a formal waiver of ethical approval<br>• Include an approval number if one was obtained<br>• If the study involved *non-human primates*, add *additional details* about animal welfare and steps taken to ameliorate suffering<br>• If anesthesia, euthanasia, or any kind of animal sacrifice is part of the study, include briefly which substances and/or methods were applied<br><br>**Field Research**<br><br>Include the following details if this study involves the collection of plant, animal, or other materials from a natural setting:<br>• Field permit number<br>• Name of the institution or relevant body that granted permission	
**Data Availability**<br><br>Authors are required to make all data underlying the findings described fully available, without restriction, and from the time of publication. PLOS allows rare exceptions to address legal and ethical concerns. See the PLOS Data Policy and FAQ for detailed information.	Yes - all data are fully available without restriction

A Data Availability Statement describing where the data can be found is required at submission. Your answers to this question constitute the Data Availability Statement and **will be published in the article**, if accepted.<br><br>**Important:** Stating 'data available on request from the author' is not sufficient. If your data are only available upon request, select 'No' for the first question and explain your exceptional situation in the text box.<br><br>Do the authors confirm that all data underlying the findings described in their manuscript are fully available without restriction?	
**Describe where the data may be found in full sentences. If you are copying our sample text, replace any instances of XXX with the appropriate details.**<br><br>• If the data are **held or will be held in a public repository**, include URLs, accession numbers or DOIs. If this information will only be available after acceptance, indicate this by ticking the box below. For example: *All XXX files are available from the XXX database (accession number(s) XXX, XXX.*).<br>• If the data are all contained **within the manuscript and/or Supporting Information files**, enter the following: *All relevant data are within the manuscript and its Supporting Information files.*<br>• If neither of these applies but you are able to provide **details of access elsewhere**, with or without limitations, please do so. For example:<br><br>*Data cannot be shared publicly because of [XXX]. Data are available from the XXX Institutional Data Access / Ethics Committee (contact via XXX) for researchers who meet the criteria for access to confidential data.*<br><br>*The data underlying the results presented in the study are available from (include the name of the third party*	All data obtained by the citizen monitroing are available on the website: https://five.epicollect.net/project/wasaf

and contact information or URL).<br>• This text is appropriate if the data are owned by a third party and authors do not have permission to share the data.<br><br>* typeset	
Additional data availability information:	

Manuscript

Click here to access/download;Manuscript;Manuscript unmarked version.docx

Can participatory approaches strengthen the monitoring of cyanobacterial blooms in developing countries? Results from a pilot study conducted in the Lagoon Aghien (Ivory Coast)

Veronica Mitroi[1], Kouadio Chrislain Ahi[2], Pierre-Yves Bulot[1,3], Fulbert Tra[2], José-Frédéric Deroubaix[4], Mathias Koffi Ahoutou[5,6], Catherine Quiblier[7,8], Mariatou Koné[2], Julien Coulibaly Kalpy[5], Jean-François Humbert[1*]

1. INRAE-Sorbonne Université, iEES Paris, Paris, France
2. Université Félix Houphouët Boigny, Institut d'Ethnosociologie, Abidjan, Côte d'Ivoire
3. INRAE-LISIS, Cité Descartes, Marne-la-Vallée, France
4. LEESU, École des Ponts ParisTech, Champs-sur-Marne, France
5. Institut Pasteur d'Abidjan, Abidjan, Côte d'Ivoire
6. Université Jean Lorougnon Guédé, Daloa, Côte d'Ivoire
7. MNHN, UMR 7245 Molécules de Communication et Adaptation des Micro-organismes, Paris, France
8. Université de Paris, Paris, France

* Corresponding author, jean-francois.humbert@upmc.fr

Short Title: Citizen monitoring of cyanobacteria in Africa

Key words: Citizen monitoring, cyanobacteria, Lagoon Aghien, Ivory Coast

Abstract

Monitoring of cyanobacteria in freshwater ecosystems is a complex task, which is time consuming and expensive due to the chaotic population dynamics and highly heterogeneous distribution of cyanobacteria populations in water bodies. The financial cost constitutes a strong limitation for the implementation of long-term monitoring programs in developing countries, particularly in Africa. The work presented here was performed in the framework of an international project addressing the sustainable monitoring and management of surface water resources used for the production of drinking water in three African countries. We tested the potential of a citizen approach for monitoring cyanobacterial blooms, which are a growing threat to the drinking water supply. This pilot study was designed, implemented and evaluated in close interaction with the Pasteur Institute of the Ivory Coast and with the populations of three villages located on the shoreline of a freshwater lagoon located near Abidjan city. Based on the use of a smartphone application, the citizens of the three villages were invited to report water color changes, as these changes could reflect cyanobacteria proliferations. A two-year experimentation period has shown that it is possible to mobilize the local populations to monitor cyanobacterial blooms. The data collected by citizens were consistent with the data obtained by a classical monitoring of cyanobacteria performed over seven months, but it appeared that new approaches were needed to validate the citizen data. This participatory approach also provided great improvements to the understanding and awareness of local populations regarding water quality and cyanobacterial bloom issues. Finally, we discuss some of the difficulties and limitations of our participatory monitoring approach that should be considered by further implementations. Despite these difficulties, our work suggests that citizen monitoring is a promising approach that may complement the classical approach to sustainable monitoring of cyanobacteria in developing countries.

Introduction

Monitoring the proliferations of potentially toxic cyanobacteria in freshwater ecosystems is an essential pillar for the assessment and management of environmental and sanitary risks associated with these phenomena. Numerous studies have therefore been conducted on this issue over the last twenty years. It has been shown that (i) one of the main difficulties in cyanobacteria monitoring is due to the temporal and spatial variability in their vertical and horizontal distributions in water bodies [e.g. 1-3], meaning that the choice of sampling strategy and the tools used for sampling have great impacts on the quality of the data collected [3,4], and (ii) accurate monitoring of cyanobacteria and their associated risks is time consuming and expensive [5].

As a matter of consequence, many efforts have been made to develop and validate new innovative devices for the survey of cyanobacterial blooms. For example, probes equipped with chlorophyll-a/phycocyanin and other sensors that can be coupled with autonomous monitoring platforms are now available for punctual or continuous estimations of cyanobacteria biomasses in the water column [6,7]. In the same way, the use of remote sensing [8] and LIDAR [9] allows to estimate the horizontal and/or vertical distribution of the cyanobacterial biomass in a lake. Some of these tools remain under development but are increasingly used for cyanobacteria monitoring, particularly in developed countries. However, the cost of these devices is still a strong limitation to their use in developing countries [10].

Another strategy recently tested for the monitoring of water quality in freshwater ecosystems is the development of citizen science projects based on the involvement of the population living around the targeted freshwater ecosystems [11]. Some of these projects are specifically concerned with the monitoring of cyanobacterial blooms as they are easy to report by nonscientist actors due to changes occurring in the water color or the presence of colonies or aggregated filaments distinguishable by the naked eye (cf. Fig. 1B). In the USA, the

CyanoTRACKER project is based on the use of a smartphone application by volunteers to report algae blooms in their neighborhood pond and/or lake [12]. In the same way, citizen science programs based on the collection of water samples have been implemented for the monitoring of lake water quality and the survey of blooms in the province of Saskatchewan (Canada) [13] and in numerous lakes located in the state of Michigan (USA) [14].

Most of these projects take place in developed countries, fewer citizen programs being available in developing countries [15]. On the African continent, for example, a participatory local observatory has been implemented in Tanzania aiming at collecting and sharing data related to water, rainfall, fisheries, agriculture and food behavior between various stakeholders in water and land management [16]. In South Africa, Nare *et al.* [17] described the difficulties of community involvement and participation in water quality and management in a catchment area due to the inadequate institutional framework. Finally, a good relationship was found between the citizen monitoring of algal blooms and standard laboratory measurements in urban lakes and ponds of three cities located in Brazil and China [18]. These programs highlight the important benefits of this collaboration in terms of both knowledge production, opportunities for democratizing knowledge and expertise under the context of growing environmental and health risks [19].

In the framework of a collaborative project between three African countries (Ivory Coast, Senegal and Uganda) and France (WaSAf project, https://humbert19.wixsite.com/wasaf), we performed a first pilot study on the feasibility of setting-up a citizen monitoring of cyanobacteria in the African context. The three main goals of our study were to evaluate (i) the potentials and constraints for the mobilization of the local populations for the visual survey of cyanobacteria in aquatic ecosystems, (ii) the consistency between the data provided by the citizen monitoring of cyanobacteria and those obtained using a classical approach based on water sampling and cell counting and (iii) the contribution of

citizen monitoring to awareness in the neighboring populations regarding the cause and health consequences of cyanobacterial blooms. This citizen monitoring approach was based on the use of a smartphone application and implemented in three villages located around a freshwater lagoon (Lagoon Aghien) in Ivory Coast.

Material & methods

Study site

Lagoon Aghien is a freshwater ecosystem located in the northeastern part of the city of Abidjan (Fig 1A). This lagoon covers a surface area of 19.5 km^2 with a perimeter of 40.72 km and a volume of 25 km^3 [20]. Lagoon Aghien is part of a network of lagoons surrounding the city of Abidjan, with the particularity of being the only one containing freshwater. This lagoon is considered a potential water resource for the city of Abidjan in complement to the water supply currently provided by groundwater extraction. Approximately 12,000 people live in the immediate proximity of the lagoon, mostly distributed in eight villages located on its shoreline (Fig 1B). The lagoon is used by local populations for various domestic (bathing, washing, cooking, waste management, etc.) and economic (fishing, agriculture, etc.) practices that have an impact and are impacted by the water quality of the lagoon. Due to growing human populations and activities, not only in the immediate environment of the lagoon but also in its whole watershed, coupled with a dramatic lack of sanitation infrastructure and changes occurring in the land use occupation, Lagoon Aghien is now eutrophicated, and cyanobacterial blooms are frequently observed (Fig 1C). Despite the crucial importance of the lagoon for drinking water production, no institutional monitoring of water quality is currently performed.

The villages Débarcadère (3000 people), Akandjé (900 people) and Aghien-Télégraphe (1850 people) were chosen for the implementation of citizen monitoring because of (i) their geographical location enabling satisfactory coverage of the lagoon (Fig 1A), (ii) the local

authorities of the three villages agreed to participate in this experiment and (iii) these villages were representative of the social organization and the various uses of the lagoon for all the villages.

Fig 1. A. Location (red dot) of the lagoon Aghien in Ivory Coast. B. Map of the Lagoon Aghien with the locations of the three villages (Débarcadère, Akandjé, Aghien-Télégraphe) in which citizen monitoring of cyanobacteria was implemented (black circles: villages involved in the citizen monitoring; white squares: other villages located around the lagoon). C. Photo of a *Microcystis* sp. (cyanobacteria) bloom in a bay of the Lagoon Aghien.
Stars S1-S6 indicate the sampling points in the monitoring performed by the Pasteur Institute of Abidjan

Ethic statement

This study was not submitted for approval by an institutional review board (ethics committee) because there is currently no committee in Ivory Coast for environmental studies. But as described latter in the paper, we organized in each of the three villages concerned by this study, a meeting with the chiefdoms in order to present the project and to ask them for their agreement before to begin to work. In addition we asked also to the chiefdoms to designate the sentinels (referent people) in each village who have provided most of the data. Finally, our smartphone application entailed the possibility to collect anonymous reports.

Social investigation for participative water quality monitoring

Sociological investigations conducted in 2017 within the WaSAf program have been used to prepare for the implementation of the participatory monitoring in the three villages. A sociological survey based on a semi-structured questionnaire was performed in all the villages

located around the lagoon in order to better understand the different uses of the lagoon and the social organization of the local populations. For the purpose of this pilot study on the citizen monitoring of cyanobacteria, we only used the two questions dealing with the perception of the water quality by the local populations (See S1 Table).

Fig 2. Global overview of the different stages in our approach on the implementation, follow-up and data restitution of citizen monitoring of cyanobacteria in the three villages of the Lagoon Aghien.

According to the recommendations of Sturm et al. [21], various actions were performed with local populations with the goal to prepare, conduct and ensure the follow-up and finally report the results of the participatory monitoring in the three villages (Fig 2). Firstly, meetings and semi-structured interviews were conducted with local community leaders and chiefdoms, and with inhabitants of the three villages in order to discuss about their interest for the implementation of a citizen monitoring of cyanobacteria. Secondly, focus groups were conducted with the more specific objective of identifying the opportunities and constraints for implementing participatory monitoring. These focus groups were organized with homogenous social categories according to gender and age (women, men and youths), including between 7 and 20 participants depending on the groups and the villages. They have permitted to find a common language between scientists and inhabitants, to better define the questionnaire in the mobile application and to generate enthusiasm among the participants to collaborate. Thirdly, a meeting was organized in each village with the goal to present and test the smartphone application with volunteers. Flyers explaining the use of the application were also distributed to participants to encourage a large participation.

To ensure regular data on the water quality of the lagoon, one person, designated as a sentinel, was chosen in each village by the local chiefdoms. In Débarcadère village, the sentinel was a school teacher, while in Akandjé village, he was the representative of the young people. Finally, in Aghien-Télégraphe, the chief of the village decided that he could fulfill this task. Each sentinel was equipped with a mobile phone provided by the WaSAf program and was encouraged to take at least one picture per week of the lagoon, always in the same location (chosen together with the research team). The sentinel was also in charge of facilitating the participation of other members of the community willing to participate in the monitoring (helping them to install and use the application, explain the interest in the monitoring, etc.).

To maintain the interest of participants and encourage them to continue contributing, we sent SMS messages of thanks to the contributors having registered their phone number in the smartphone application twice during the experiment. In accordance with the project team commitments and to facilitate data appropriation in the villages, two restitution events (based on a poster presentation) were organized in each village at the end of each year of monitoring. We also prepared two times a prospectus (called Lagoon Health Bulletin) presenting together the data collected by citizens and those obtained from the water laboratory analyses performed by the Pasteur Institute of Abidjan. Finally, some educative actions explaining cyanobacterial blooms were performed in schools of Débarcadère and Aghien-Télégraphe villages.

At the end of the two years of the pilot study, we conducted qualitative interviews with the three sentinels and other volunteers who participated in monitoring to evaluate the effectiveness and difficulties related to participatory monitoring, as well as their appropriation of the monitoring experience. Our questions concerned (i) their participation in the monitoring (how they personally experimented the monitoring process and how this was concretely done, difficulties encountered, etc.), (ii) the impact of this participation on their own perception and

understanding of the lagoon's ecological state and (iii) suggestions for improving the monitoring and its sustainability.

The participatory monitoring was organized in two periods: (i) during the first period (from October 2017 to August 2018), the sentinels were asked to provide at least one report per week, and (ii) during the second period (from September 2018 to August 2019), the sentinels were asked to collect and send reports only when something interesting concerning the water quality in general and/or the presence of a cyanobacterial bloom was detected. However, some participants other than sentinels have continued to send reports, even when there was no cyanobacterial bloom. We chose to change the strategy in the middle of the pilot study in order to determine if the sentinels remained vigilant about cyanobacteria when they no longer had the obligation to send a weekly report.

Smartphone application

The generalized access to mobile phone services endowed with geographic information systems (GIS) creates new opportunities for data collection, access and sharing all over the world [22-24]. According to data from the World Bank [25], access to cellular networks has reached 90 % in numerous African countries, while access to drinking water is only approximately 86% and access to sanitation is close to 60%. Consequently, the use of a smartphone application seemed to be a good tool for involving local communities in participatory monitoring. We adapted a free smartphone application that is part of the Epicollect5 data-gathering platform (https://five.epicollect.net/) to the specific needs of the monitoring. This solution allowed the generation of project-dedicated questionnaires and the collection of georeferenced data that could be displayed through a web application in the form of maps, tables and charts. For each entry in the WaSAf project (S1 Fig), users completed a short questionnaire asking for the presence of (i) a green water color and a visible mass of algae suspended in the water, (ii) traces

of fuel contamination, (iii) dead fish, (iv) garbage accumulation and/or if there was nothing to report or if they wanted to add other observations. For each questionnaire, users were asked to update their geo-location and to add a picture if they wished. Users can choose to respond anonymously but they could also provide their phone number. This optional information could be used by the project team to provide feedback and to confirm the importance of the collected data (two SMS campaigns were sent). At the end of each questionnaire, users recorded their data and sent them to the website if a data connection was available. If there was no internet connection, the recorded data could be sent later. As soon as a questionnaire was sent, the data were available online. All the data collected in the framework of this project are available at the project website: https://five.epicollect.net/project/wasaf.

S1 Fig. Screenshots of the Epicollect-5 smartphone application with the WaSAf project.

Characteristics of the monitoring of cyanobacteria performed by the Pasteur Institute of Abidjan

In the framework of the WaSAf project, a monitoring of the water quality in the Lagoon Aghien was performed at six sampling stations (S1-S6) from December 2016 to April 2018 by the Pasteur Institute of Abidjan (Fig 1A). Consequently, it was only possible to compare from October 2017 to April 2018, the phytoplankton data obtained by this monitoring with those resulting from the citizen monitoring of cyanobacteria. Data collected on the phytoplankton community were based on an integrated water sampling performed in the first meter of the water column according to the technique used by Laplace-Treyture *et al*. [26]. The counting of cyanobacteria was performed on an inverted microscope (Nikon Eclipse TS100) using the Utermöhl method, according to the AFNOR 15204 standard. A minimum of 30 randomly

selected fields was counted to attain at least 500 counting units (cells, colonies or filaments/trichomes).

The results were then expressed as the number of cells per milliliter. In the case of the colonial cyanobacteria *Microcystis* sp., two categories of colonies were considered regarding their diameter (< or > 200 µm). The mean number of cells per category was estimated under an upright microscope by counting the number of cells in 30 colonies per category after they were gently crushed between slides and coverslips. For the filamentous cyanobacteria *Aphanizomenon*, *Cylindrospermopsis*, *Limnothrix*, *Lyngbya*, *Oscillatoria*, *Planktothrix*, *Pseudanabaena* and *Dolichospermum*, the counting considered filament lengths of 100 µm. The mean number of cells per 100 µm of filament was estimated under an upright microscope for 30 filaments for each genus.

The mean cell biovolumes were calculated for the cyanobacteria genera that were most abundant in Lagoon Aghien (*Microcystis*, *Cylindrospermopsis*, *Limnothrix*, *Oscillatoria*, *Dolichospermum*) from measurements performed on 30 cells per genus. For each of the other genera, we used standard cell values defined in the HELCOM PEG Biovolume reports (http://ices.dk/marine-data/vocabularies/Pages/default.aspx).

Results

Overall presentation of the evolution of the number and locations of collected reports during the study period

During the first period of this pilot study (from October 2017 to August 2018), 443 reports have been recorded in the WaSAf project hosted by the Epicollect5 platform (see https://five.epicollect.net/project/wasaf). Among these 443 reports, phone numbers were only provided in 37 of them. During the second period of the study (from September 2018 to August 2019), 171 questionnaires were collected and phone numbers were provided in only 7 of them.

During the whole study period, it appears that 92.8% of the collected data with the smartphone application were anonymous, which makes it impossible to estimate the number of participants who have contributed to the monitoring.

During the first period of the study, an average of 44 reports was collected each month in the web site of our application, while during the second period, an average of 13 reports were collected monthly (Fig 3). This difference in the number of collected reports during the two periods was expected since we changed the recommendation for the sentinels from sending a picture at least once a week (regardless of whether cyanobacteria were observed) to sending pictures only if cyanobacteria or other degradation signs were observed.

Fig 3. Number of completed questionnaires loaded on the WaSAf project Epicollect5 platform. During the first period, the sentinels were asked to complete at least one questionnaire per week with one picture of the lagoon. During the second period, sentinels were advised to send pictures only if cyanobacteria or other worrying phenomena were observed.
Dashed line: Monthly average of collected questionnaires during each period.

As shown in Fig 4A, the geographical origins of the data collected during this study were distributed all around the lagoon and not only near the three villages. Many data points were collected in the western part of the lagoon by the sentinel from the Débarcadère village (Fig 4B). This sentinel performed visual monitoring at four points in this part of the lagoon. Finally, it also appears on the map that some of the data were not located on the shoreline of the lagoon.

Fig 4. Distribution of the reports collected by citizens around the Lagoon Aghien (A). Detail on the citizen monitoring performed by the sentinel from Débarcadère village (B).

The size of the circles is proportional to the number of reports collected in each place. For the places where more than five reports were collected, the exact number of collected reports is provided into the circle.

Data indicating the potential presence of cyanobacterial blooms

The potential presence of cyanobacterial blooms in the lagoon was reported in the smartphone application by the response “Green water, visible algae” in the questionnaire. These responses were sometimes supported by the pictures associated with the questionnaire (see for example two pictures in S2 Fig) but frequently the poor quality of pictures did not permit us to confirm the presence of cyanobacteria. As shown in Fig 5, cyanobacteria blooms were reported mainly during four periods. The first peak of reports occurred in November 2017, followed by a small peak in February 2018, a third peak in May-June 2018 and finally a fourth large peak from May to September 2019.

Fig 5. Evolution of the number of reports made by citizens on the potential presence of cyanobacteria in the Lagoon Aghien (green water color and/or presence of visible colonies)

S2 Fig. Two examples of bloom pictures taken by the participants during the citizen monitoring of the Lagoon Aghien (all the pictures are available on the website (https://five.epicollect.net/project/wasaf)

The geographical locations of the potential algal blooms indicated by the presence of a green water color/visible algae during the three periods were not the same. For the first peak (November 2017), almost all reports were from the Débarcadère village, while for the two other

peaks (May-June 2018 and May-September 2019), almost all reports of algal blooms were from the Aghien-Télégraphe village.

Comparison of the data provided by the participatory monitoring with those from the Pasteur Institute

It was difficult to compare the data collected by the Pasteur Institute of Abidjan with that collected by the citizens. Indeed, the citizen monitoring was performed on the shores of the lagoon while the monitoring of cyanobacteria by the Pasteur Institute of Abidjan was performed in six sampling points located more than 150 m away from the shores (except for S3). Moreover, water samples were collected by Pasteur Institute once a month while the smartphone reports were sent by citizens as the days went by. For these reasons, we have only compared the monthly number of reports mentioning the presence of a green water color/visible algae with the monthly variations in the total cyanobacterial biovolumes estimated at each sampling stations.

Two increases in cyanobacterial biovolumes were observed during the monitoring performed by the Pasteur Institute from October 2017 to April 2018. The first increase was recorded in November 2017, and the second one was recorded in February 2018 (Fig 6). Interestingly, during these two increases of cyanobacterial biovolumes, there was also an increase in the number of citizen reports mentioning green water color/visible algae. During the seven months of the Pasteur Institute monitoring, cyanobacteria dominated the phytoplankton community, representing on average 40% (±18) of the total phytoplankton biovolumes estimated at each date and sampling station, when Dinophyta, Chlorophyta, Bacillariophyta and Euglenozoa represented respectively 19% (±14), 17% (±11), 15% (±17) and 9% (±6) of these biovolumes. Finally, the data collected by Pasteur also highlighted that large variations

occurred in cyanobacterial biovolumes depending on the sampling stations, with the largest biovolumes being recorded during this study period at Station 4 and the lowest at Station 6.

Fig 6. Cyanobacterial biovolumes estimated at the six sampling stations of the Lagoon Aghien by the Pasteur Institute and number of reports mentioning a green water color / visible algae made on the smartphone application by citizens.

CM = Citizen monitoring. S1 to S6 = Sampling stations 1 to 6 (see Fig 1)

Local understanding of the issues of water quality and cyanobacteria in the lagoon

From the social investigation performed before the implementation of the citizen monitoring, significant differences (Chi2 test, $p<0.0001$) were found in the opinions about the water quality of the lagoon in the three villages (Fig 7A). Almost 90% of the interviewed people from the Akandjé village and 56% from Aghien-Télégraphe told us that the lagoon exhibits "good and very good quality". On the other hand, the quality perception was more negative in the Débarcadère village (72% of interviewed people think that the lagoon is in a bad or very bad state).

Significant differences were also found between the three villages in the criteria used by local populations to qualify the recent evolution of the water quality in the lagoon (Chi2 test, $p<0.001$). These differences mainly concern the proliferation of reeds and the bad smells, which were more specifically associated with the degradation of the water quality in Aghien-Telegraphe and Débarcadère respectively (Fig. 7B). On the other hand, the two most cited criteria used to qualify the recent degradation of the water quality in the three villages were the fishing captures (a decrease over the last 20 years according to their perception) and the changes occurring in the water color (Fig 7B). The relative importance of these two criteria varied

according to the villages, with significant differences between Akandjé and the two other villages (Chi2 test, $p<0.05$).

Fig 7. Perception of the water quality in Lagoon Aghien by residents of the three villages (Aghien-Télégraphe, Akandjé, Débarcadère). Respondents were asked (A) how they evaluate the quality of the lagoon using a four-point scale (very good, good, bad, very bad) and (B) which criteria they use for the assessment of the water quality.

Finally, the focus groups and qualitative interviews have revealed that two main changes in the water color are clearly identified by the local populations: (i) brown water (muddy, reddish) observed mainly during the rainy seasons (from May to July and from September to November), and (ii) green water observed before and/or after the rainy seasons. The brown color was (mainly) associated with “dirty water” coming from the watershed during the rainy periods. During these periods, inhabitants take precautions, and the lagoon is less used for domestic activities to avoid water-related diseases such as diarrhea. The green color of the lagoon was not associated with any sanitary or environmental risks. In contrast, some participants noticed a coincidence between green water color and fish abundance: "*When the water turns green, it smells such as fish, it is fish poop, it is to tell the fishermen that there is a lot of fish and that they can go out fishing*” (Focus group women, Akandjé, Nov. 2017). Only some of the villagers noticed that bathing activities during these green water periods could generate skin and eye irritation. Consequently, no or very few precautions related to domestic uses of water are taken during cyanobacteria blooms. Finally, many participants of the focus groups made a link between the brown color (the runoff pollution) and the green color (eutrophication), with the first color often preceding the second.

The initial focus groups and the restitution events represented important opportunities for explaining the reasons why researchers and members of the Pasteur Institute were so interested in the monitoring of the “green spots”, as local people called them. On the one hand, local observations are relevant (the phytoplankton biomass production fosters the fish populations), but it has to be stated and explained that a massive development of cyanobacteria also represents a sanitary risk for human activities and can be considered a good indicator of the overall evolution of the water quality in the lagoon. On the second hand, such moments of exchange are necessary to motivate the local communities and raise local concern about cyanobacterial blooms, which were previously considered as a natural phenomenon.

Finally, the initial focus groups and the restitution events played a role of “hybrid arenas” for the communities and scientists to discuss about the causes and consequences of the degradation of the water quality in the lagoon with scientists. Local chiefdoms were very willing to discuss about the contribution to the pollution of the lagoon by populations living close to the lagoon compared to that originating from the rest of the watershed and the actions and programs that could be implemented to protect/restore the water quality in the lagoon.

Discussion

Although the participation of nonscientists in environmental monitoring is not a recent practice, the phenomenon has undergone spectacular development over the last twenty years, facilitated by new communication technologies and mobile computing applications. This first citizen project based on the use of a smartphone application for the monitoring of cyanobacterial blooms in Africa shows that there is an opportunity for such approaches for both (i) the improvement of the monitoring of cyanobacteria in addition to the monitoring performed by relevant institutions and (ii) the improvement of the awareness and social learnings for the local

communities concerning the health risks associated with cyanobacteria and more globally about the degradation of the water quality.

Concerning the improvement of institutional monitoring, as mentioned in the introduction, it is well known that accurate monitoring of cyanobacterial blooms is very costly. This financial cost is a major constraint for the implementation of long-term monitoring in developing countries because of the difficulty of securing long-term funding in these countries. The best illustration of this issue was that the monthly monitoring performed by the Pasteur Institute of Abidjan, at the same time as the participatory monitoring implementation, was stopped because of the lack of financial resources to continue it. Our pilot study has shown that it is possible to mobilize local populations living close to a freshwater ecosystem to monitor it. In this context, it is interesting to raise the issue of the place of this participatory monitoring in an overall monitoring system for the Lagoon Aghien. First, citizen monitoring could be an early warning system for cyanobacterial blooms, which could trigger water samples and analyses. This system could significantly (i) improve the efficiency of institutional monitoring based, for example, on a monthly sampling frequency in a limited number of sampling points and (ii) reduce the cost of institutional monitoring if these two approaches are well coordinated. Second, citizen monitoring coupled with education and capacitation programs could also permit improved basic knowledge of the dynamics and toxicity of cyanobacterial blooms and, more globally, the knowledge of water quality, when coupled with water sampling by untrained and equipped volunteers, as has been done in other participatory projects [13].

Concerning the local awareness and social learnings about cyanobacteria, the focus groups showed that local populations living around the Lagoon Aghien could spontaneously identify visible pollution, as well as changes in physical and biological parameters (such as the water color, macrophytes and algal blooms). At the same time, these focus groups also revealed the low awareness of these populations concerning the health risks associated with

cyanobacterial blooms. During the preparation and feedback meetings organized in the three villages, there were also many discussions on the causes of cyanobacterial blooms in freshwater ecosystems. The issue of eutrophication was well understood by most of the participants, with interesting debates on the relative contribution of nutrient discharge into the lagoon from local pollution *versus* “distant pollution” from the other parts of the Abidjan district located in the watershed. These discussions, for example, led the chiefdom representatives of one village to prohibit defecation in the lagoon by the inhabitants of this village and asked for sanitation investments from the regional authorities. Finally, it appears that the social benefits associated with the citizen monitoring approach are both direct (new learning and understanding of water ecosystem functioning and the eutrophication issue and its consequences to cyanobacterial blooms; new capacities to identify pollution sources and sanitary and ecological risks, etc.) and indirect (their appropriation and translation into conservation actions, etc.).

Several limits were also identified during this first pilot study on the citizen monitoring of cyanobacteria in the Ivory Coast. These limits can be qualified as (i) technical limits; (ii) participatory limits; (iii) data accuracy and validation limits and (iv) institutional limits.

The first technical limits are related to the accuracy of the produced data. Some data were affected by the approximate geolocations of several reports, which were not located on the shoreline of the lagoon. These geo-localization errors were probably due to the misuse or poor quality of the GPS of some smartphones, knowing that the rapid enhancement of smartphone quality will probably reduce this problem in the coming years. In the same way, the poor quality of some of the pictures associated with the questionnaires did not permit visual confirmation of the presence of "green algae" indicated in the questionnaire. Again, it is likely that the rapid enhancement of camera quality in smartphones [27] will permit us to solve this problem in the future. Finally, we observed during the meetings that most of the young men and women (<45 years old) had smartphones equipped with a camera, but this was not the case

for people >45 years old, who had only small cellphone without a camera. It is likely that the decrease in the prices of smartphones will result in the future, in a more widespread availability of these devices in the populations.

The participatory limit in our pilot study concerns the fact that despite a very important number of registered data, most of them were supposed to be sent by the local sentinels. It was seldom possible to know who sent the reports because few people have provided their phone number in the reports. However, when the phone number was provided, it appeared that the sentinels of the three villages sent most of the reports. It is likely that some social/gender categories do not seem to contribute to citizen monitoring such as for example, women, although they are in close contact with the lagoon as they have multiple uses for it. However, from the interviews with the sentinels, it appeared that some villagers who observed changes in the water color asked the sentinels to create a report on the application. Thus, the role of sentinels was very important in the villages, which made the selection of sentinels very important. In our experiment, the best contributor as a sentinel was clearly the schoolteacher from the Débarcadère village, who self-planned to conduct multipoint monitoring. As noted by Requier *et al*. [28], the issue of long-term participation and the improvement of volunteer participation is facilitated by key actors (such as sentinels) who can act as intermediaries between scientists and local populations.

We identified several limits in our process of validating data provided by the citizen monitoring. Some congruence was found between these data and that collected by the Pasteur Institute but this comparison was not fully satisfactory. It would be preferable to perform a monitoring of the water in the same areas where green water color/visible algae were reported by citizens. To achieve this goal, we recommend two different methods for the validation of data collected by citizens. The first one will be based on the use of a smartphone spectrometer by the sentinels in order to evaluate some water quality parameters [29] while the second will

consist of water collection and fixation with lugol's iodine solution, then a microscope examination by scientists. These two methods will be tested in France during the summer 2020 in the framework of a citizen monitoring of cyanobacteria with the same approach used in Ivory Coast.

The institutional limit in our citizen monitoring approach concerns the integration of the data collected by the citizens within the framework of the institutional monitoring of the lagoon, which is actually missing. The two ministries in charge of the management of water resources and drinking water production have expressed great interest in this participatory approach but failed to see how the collected data could be integrated by their institutions. This result was not because participatory data were considered useless or not accurate enough but because the institutions do not devote the financial and human resources needed for long-term monitoring of freshwater resources. A great deal of awareness-raising among these institutions still needs to be done to show how citizen empowerment might be integrated in the framework of the implementation of long-term monitoring of freshwater resources. In this goal, we are currently working with the different water institutions of Ivory Coast (Water and Forests Ministry and Hydrology Ministry) for the implementation of an environmental observatory of the Lagoon Aghien, in which citizen monitoring would take its place as an early warning system for cyanobacterial blooms. We hope to reach this objective before the end of the year knowing that citizens have shown their willingness to continue the monitoring of cyanobacteria as shown by the fact that they still send reports while citizen monitoring ended officially one year ago.

Finally, in addition to the design of a smartphone application and the collection of data on cyanobacterial blooms in an African freshwater ecosystem, our citizen monitoring project considered the social aspects of participatory science. These social factors are crucial because they are both a condition for effective participatory monitoring and a condition for maximizing the social utility of the procedure. From this perspective, participatory science is much more

than recruitment of volunteers for data collection. The empowerment of knowledgeable citizens, the adoption of critical thinking skills related to the environment and the strengthening of science-society relationship are acknowledged as one crucial point of participatory science projects, even if these points are sometimes marginal in the design of participatory science projects [30, 31].

In conclusion, this pilot study conducted in Ivory Coast has shown that it is feasible to mobilize local populations in an African country for the monitoring of cyanobacteria. In particular, the choice of having one referent sentinel per village seems to be a good strategy. We also show that this kind of approach, from its initial preparation with local populations to its realization, has improved their awareness on the global issue of water quality. One important issue arising from this study concerns the validation of the data provided by the citizens, which was not fully achieved. In order to solve this problem, we proposed to test two strategies for the validation of data, which will require collaboration between sentinels and scientists. Finally, the greatest challenge of the citizen monitoring of cyanobacteria but also more generally of all the participatory approaches, concerns their sustainability, knowing that this issue will be handled differently in developed and developing countries. In numerous developed countries where there is already an institutional monitoring of cyanobacteria, the challenge of the sustainability of the citizen monitoring is to show how it can contribute to improve the institutional monitoring of cyanobacteria in order to legitimize its place. For most of the developing countries where there is no existing institutional monitoring, the challenge is to coordinate the joint implementation of these two approaches (institutional and citizen) in order to maximize their efficiency and to minimize the global cost of the monitoring, which is a key point for its sustainability.

Acknowledgments

We are extremely grateful to the three sentinels (Mr. Blaise Lattho in Aghien-Télégraphe, Mr. Kouandjo Guy Rolland Adon in Akandjé, and Mr. Kameleman Siaka Issoufou in Débarcadère) and the inhabitants and chiefdoms of the three villages (Aghien-Télégraphe, Akandjé and Débarcadère) for their decisive contribution to this work. Finally, we would like also to thank the three reviewers of this paper for their constructive comments. This work was supported by the French Facility for Global Environment (Fonds Français pour l'Environnement Mondial, FFEM) (research program WaSAf).

References

1. Welker M, Döhren von H, Täuscher H, Steinberg CEW, Erhard M. Toxic *Microcystis* in shallow lakes Müggelsee (Germany) - dynamics, distribution, diversity. Arch Hydrobiol 2003; 157: 227-248.
2. Cuypers Y, Vinçon-Leité B, Groleau A, Tassin B, Humbert JF. Impact of internal waves on the spatial distribution of *Planktothrix rubescens* (cyanobacteria) in an alpine lake. ISME J 2011; 5: 580-589.
3. Pobel D, Robin J, Humbert JF. Influence of sampling strategies on the monitoring of cyanobacteria in shallow lakes: Lessons from a case study in France. Water Res 2011; 45: 1005-1014.
4. Ahn CY, Joung SH, Park CS, Kim HS, Yoon BD, Oh HM. Comparison of sampling and analytical methods for monitoring of cyanobacteria-dominated surface waters. Hydrobiologia 2008; 596: 413-421.
5. Steffensen DA. Economic cost of cyanobacterial blooms. In: Hudnell H.K. (eds) Cyanobacterial harmful algal blooms: State of the science and research needs. Adv Exp Med Biol 2008; vol 619. Springer, New York, NY
6. Le Vu B, Vinçon-Leite B, Lemaire B, Bensoussan N, Calzas M, Drezen C, et al. 2011. High-frequency monitoring of phytoplankton dynamics within the European water framework directive: application to metalimnetic cyanobacteria. Biogeochemistry 2011; 106: 229-242.
7. Garneau MV, Posch T, Hitz G, Pomerleau F, Pradalier C., Siegwart R, et al. Short-term displacement of *Planktothrix rubescens* (cyanobacteria) in a pre-alpine lake observed using an autonomous sampling platform. Limnol Oceanogr 2013; 58: 1892-1906.

8. Hunter PD, Tyler AN, Willby NJ, Gilvear D.J. The spatial dynamics of vertical migration by *Microcystis aeruginosa* in a eutrophic shallow lake: A case study using high spatial resolution time-series airborne remote sensing. Limnol Oceanogr 2008: 53; 2391-2406.
9. Moore TS, James H, Churnside JH, Sullivan JM, Twardowski MS, Nayak AR, et al. Vertical distributions of blooming cyanobacteria populations in a freshwater lake from LIDAR observations. Remote Sens Env 2019; 225: 347-367.
10. Dube T, Mutanga O, Seutioali K, Adelabu S, Shoko C. Water quality monitoring in sub-Saharan African lakes: a review of remote sensing applications. Afr J Aquat Sci 2015; 40: 1-7.
11. Poisson AC, McCullough IM, Cheruvelil KS, Elliott KC, Latimore JA, Soranno PA. Quantifying the contribution of citizen science to broad-scale ecological databases. Front Ecol Environ 2020; 18: 19-26.
12. Mishra DR, Kumar A, Ramaswamy L, Boddula VK, Das MC, Page PM, Weber SJ. CyanoTRACKER: A cloud-based integrated multi-platform architecture for global observation of cyanobacterial harmful algal blooms. Harmful Algae 2020; 96: 101828.
13. Bos JS, Nanayakkara L, Hulbert M, Finlay K. Citizen science for Saskatchewan lakes: a pilot project. Lake Reserv Manage 2019; 35; 77-89.
14. Sarnelle O, Morrison J, Kaul R, Horst G, Wandell H, Bednarz R. Citizen monitoring: Testing hypotheses about the interactive influences of eutrophication and mussel invasion on a cyanobacterial toxin in lakes. Water Res 2010; 44: 141-150.
15. Pocock MJO, Roy HE, August T, Kuria A, Barasa F, Bett J et al. Developing the global potential of citizen science: Assessing opportunities that benefit people, society and the environment in East Africa. J Appl Ecol 2019; 56: 274-281.

16. Duvail S, Paul JL, Hamerlynck O, Majule A, Nyingi WD, Mwakalinga A et al. 2017. Recherches participatives en Tanzanie : un observatoire local pour un dialogue autour de la gestion des territoires et de l'eau. Nat. Sci. Soci. 2017 ; 25 : 347-359.
17. Nare L, Odiyo JO, Francis J, Potgieter N. Framework for effective community participation in water quality management in Luvuvhu catchment of South Africa. Phys Chem Earth 2011; 36: 1063-1070.
18. Castilla EP, Cunha DGF, Lee FWF, Loiselle S, Ho KC, Hall C. Quantification of phytoplankton bloom dynamics by citizen scientists in urban and peri-urban environments. Environ Monit Assess 2015; 187: 690
19. Buytaert W, Zulkafli Z, Grainger S, Acosta L, Alemie TC, Bastiaensen J et al. Citizen science in hydrology and water resources: opportunities for knowledge generation, ecosystem service management, and sustainable development. Front Earth Sci 2014; 2: 51-71.
20. Koffi SE, Koffi TK, Perrin JL, Seguis L, Guilliod M, Goné DL et al. Hydrological and water quality assessment of the Aghien Lagoon hydrosystem (Abidjan, Côte d'Ivoire). Hydrolog Sci J 2019; 64: 1893-1908.
21. Sturm U, Schade S, Ceccaroni L, Gold M, Kyba CMK, Claramunt B et al. Defining principles for mobile apps and plaftorms development in citizen science. RIO 2018; e23394.
22. Silvertown J. A new dawn for citizen science. Trends Ecol Evol 2009; 24: 467-471.
23. Brovelli MA, Minghini M, Zamboni G. Public participation in GIS via mobile applications. ISPRS J Photogramm 2016; 114: 306-315.
24. Mazumdar S, Ceccaroni L, Piera J, Hölker F, Berre AJ, Arlinghaus R et al. Citizen science technologies and new opportunities for participation. In: Hecker, S., Haklay, M., Bowser,

A., Makuch, Z., Vogel, J. & Bonn, A. Citizen Science: Innovation in Open Science, Society and Policy. UCL Press, London. 2018; Chap 21: 303-320.

25. World Bank. Water Hackathon - Lesson learned. 2012; Available on https://www.un.org/waterforlifedecade/pdf/2012_world_bank_water_hackathon_lessons.pdf).

26. Laplace-Treyture C, Barbe J, Dutartre A, Druart JC, Rimet F, Anneville O. Protocole standardisé d'échantillonnage, de conservation, d'observation et de dénombrement du phytoplancton en plan d'eau pour la mise en œuvre de la DCE : version 3.3.1. Cemagref, 2009 ; 44 p.

27. Coccia M. Functionality development of product innovation: An empirical analysis of the technological trajectories of smartphone. J Econ Libr 2018; 5:241-258.

28. Requier F, Andersson GKS, Oddi FJ, Garibaldi LA. Citizen science in developing countries: how to improve volunteer participation. Front Ecol Environ 2020; 18: 101-108.

29. McGonigle AJS, Wilkes TC, Pering TD, Willmot JR, Cook JM, Mims II FM, Parisi A. Smartphone spectrometers. Sensors 2018; 18:223.

30. Dickinson JL, Sirk J, Bonter D, Bonney R, Crain RL, Martin J et al. The current state of citizen science as a tool for ecological research and public engagement. Front Ecol Environ 2012; 10: 291-297.

31. Bonney R, Shirk JL, Phillips TB, Wiggins A, Ballard HL, Miller-Rushing AJ et al. Next steps for citizen science. Science 2014; 343: 1436-1437.

 Click here to access/download;Figure;Table 1.docx

	Débarcadère	Aghien-Télégraphe	Akandjé
**Questionnaires**	46	44	29
**Meetings with local chiefdoms**	2	3	2
**Focus groups**	3 (women, men, youth)	3 (women, men, youth)	2 (women, men & youth)
**Training on the use of the smartphone application**	1	1	1
**Data restitution**	2	2	2
**Follow up interviews**	6	7	3

Fig 1

Click here to access/download;Figure;Fig 1.pptx

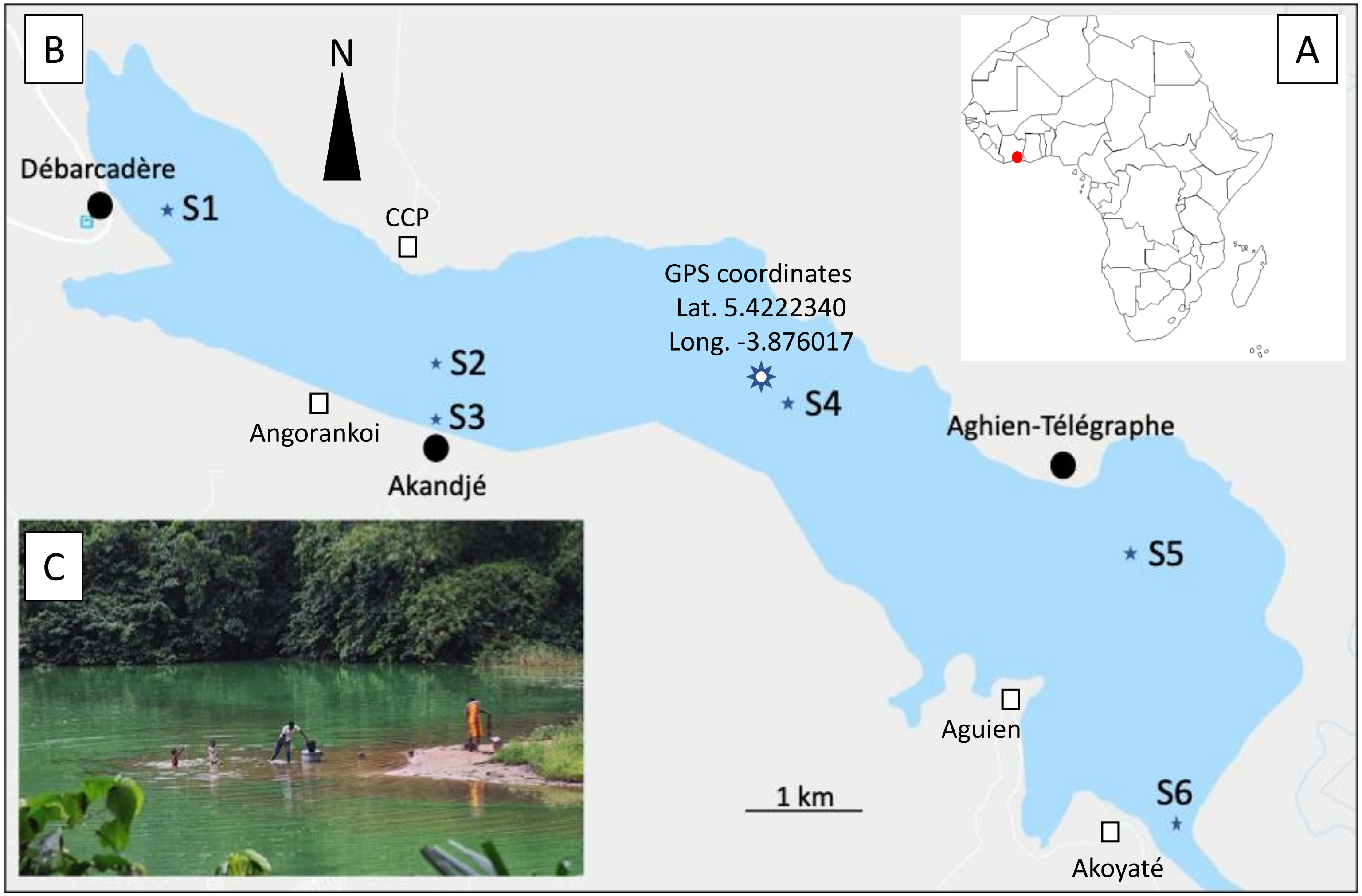

Fig 2

Click here to access/download;Figure;Fig 2.pptx

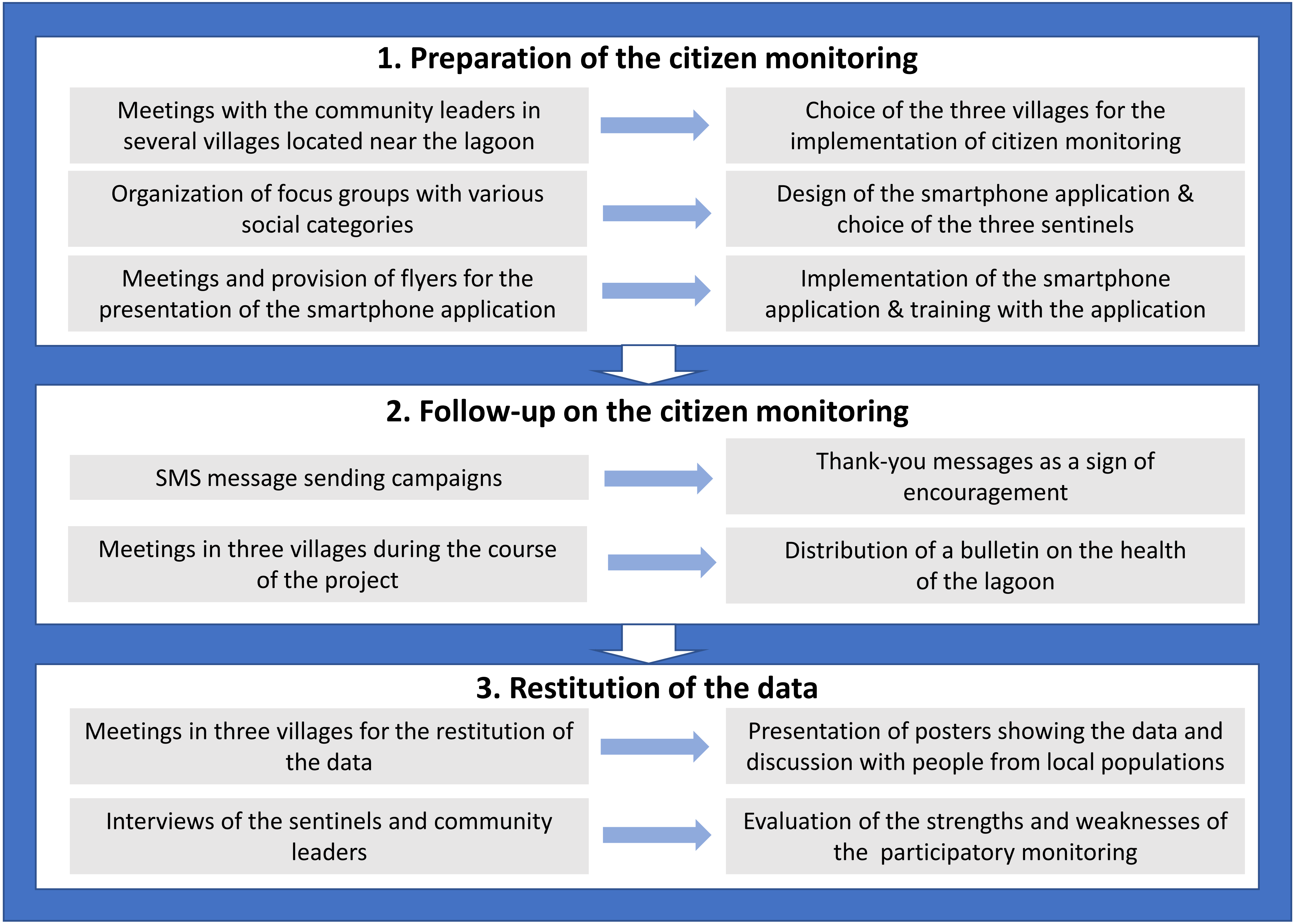

Fig 3

Click here to access/download;Figure;Fig 3.pptx

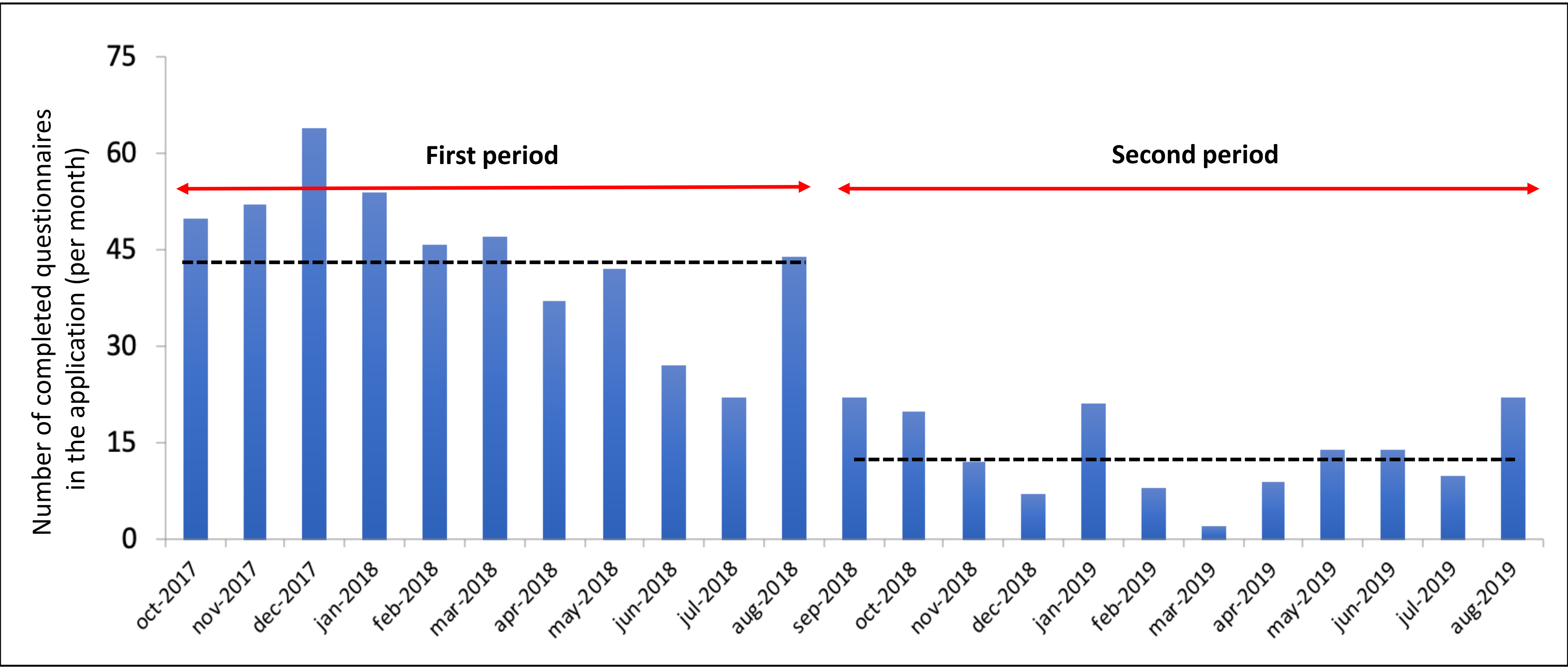

Fig 4

Click here to access/download;Figure;Fig 4.pptx

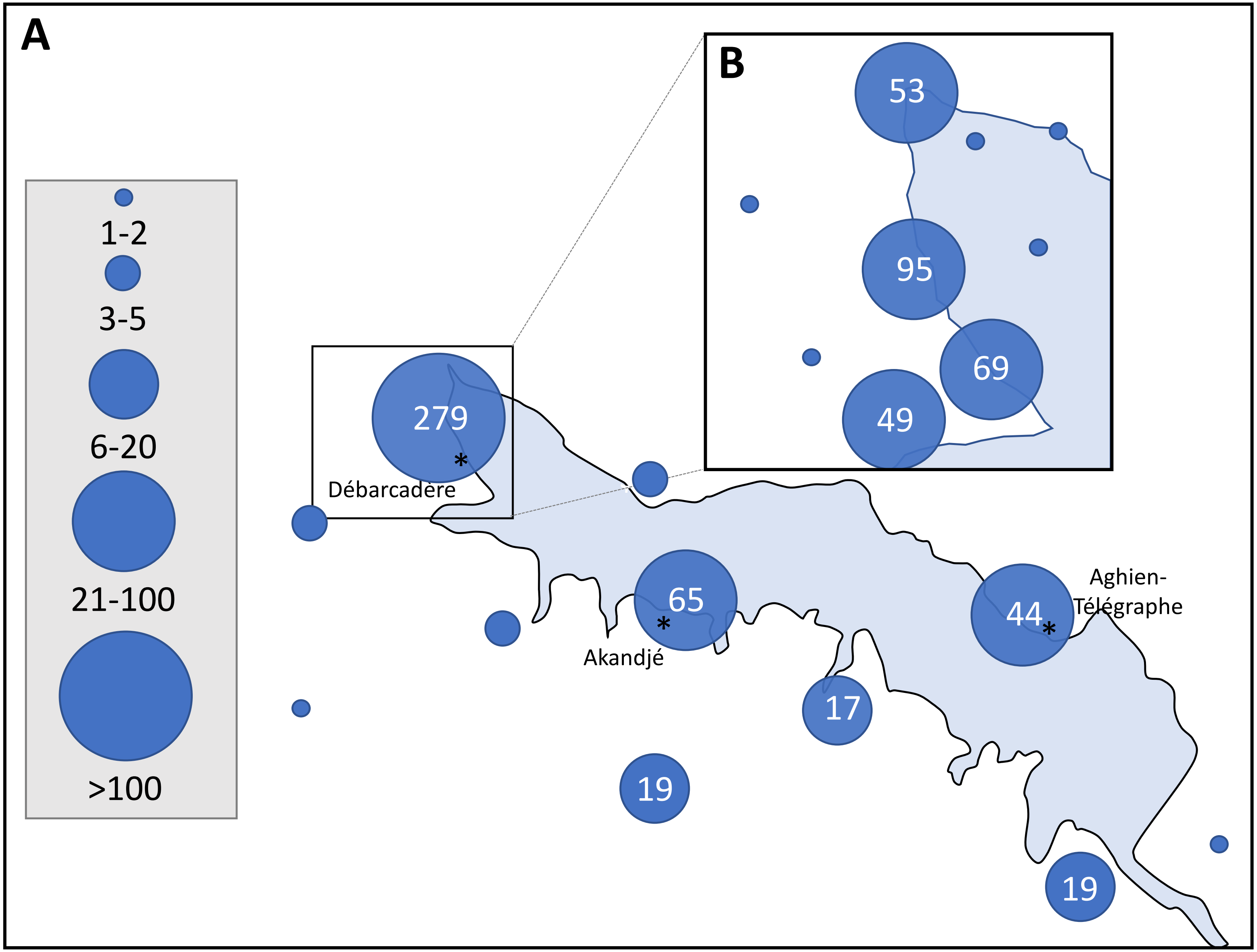

Fig 5 Click here to access/download;Figure;Fig 5.pptx

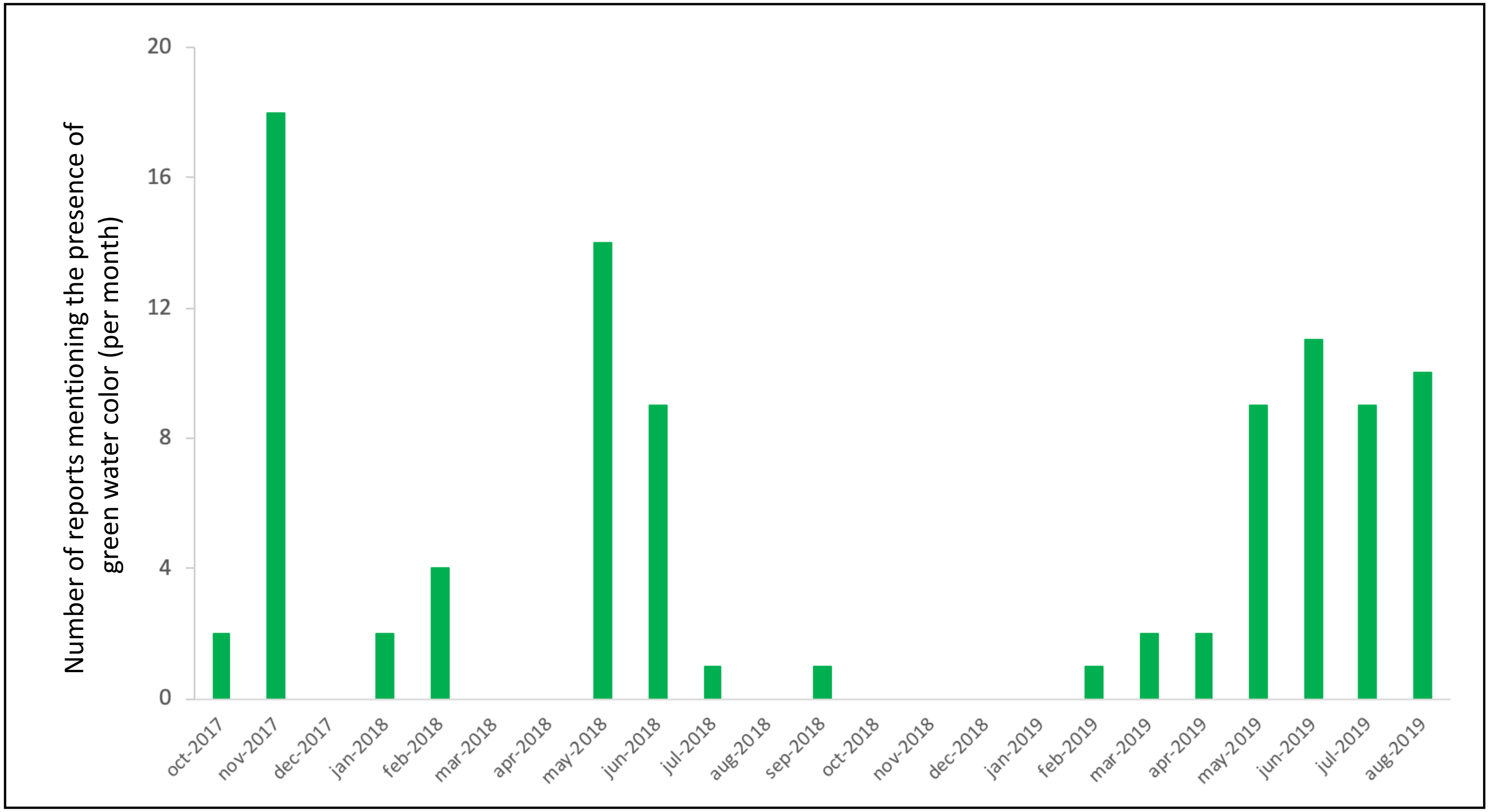

Click here to access/download;Figure;Fig 6.pptx

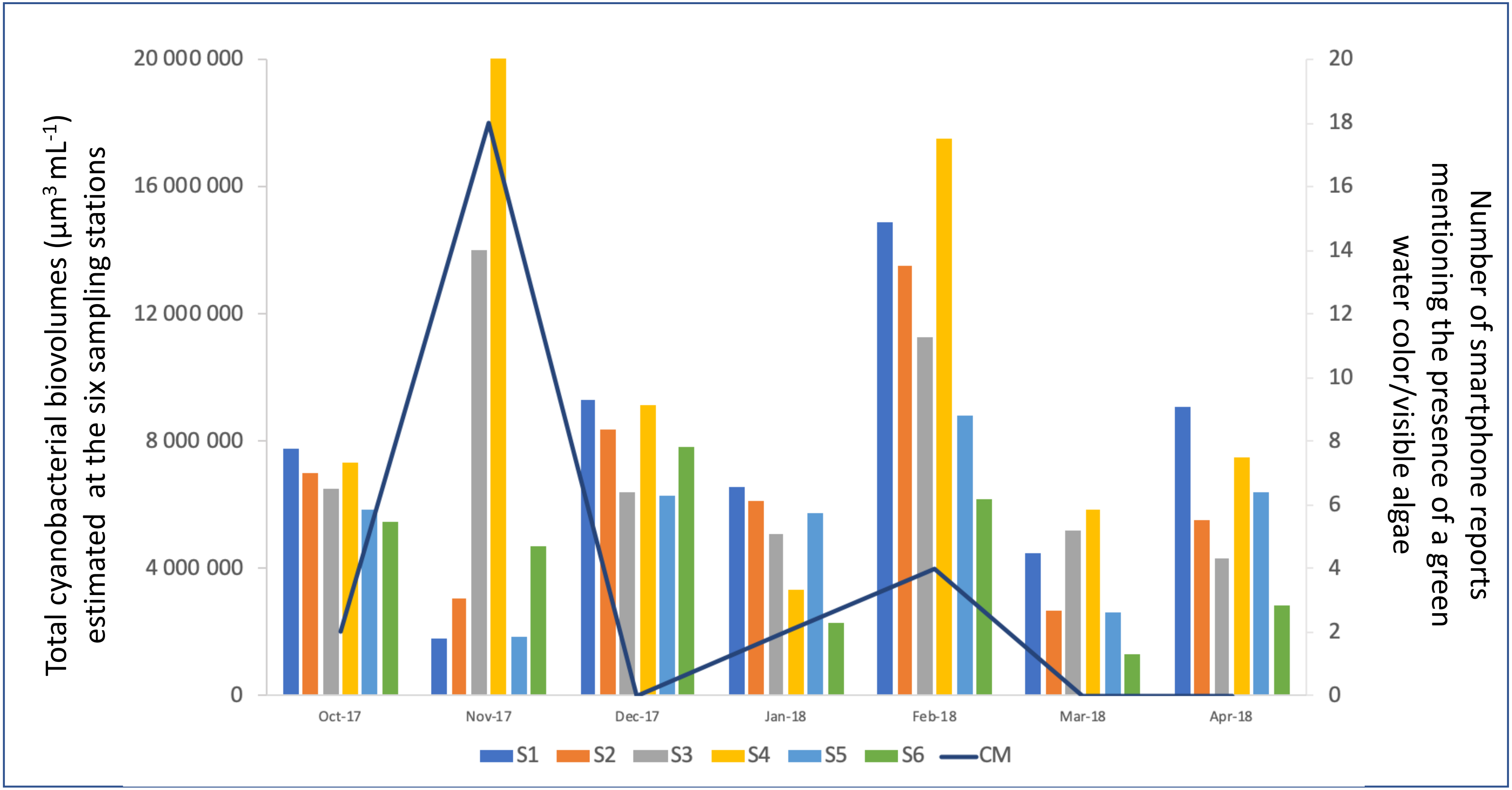

Fig 7

Click here to access/download;Figure;Fig 7.pptx

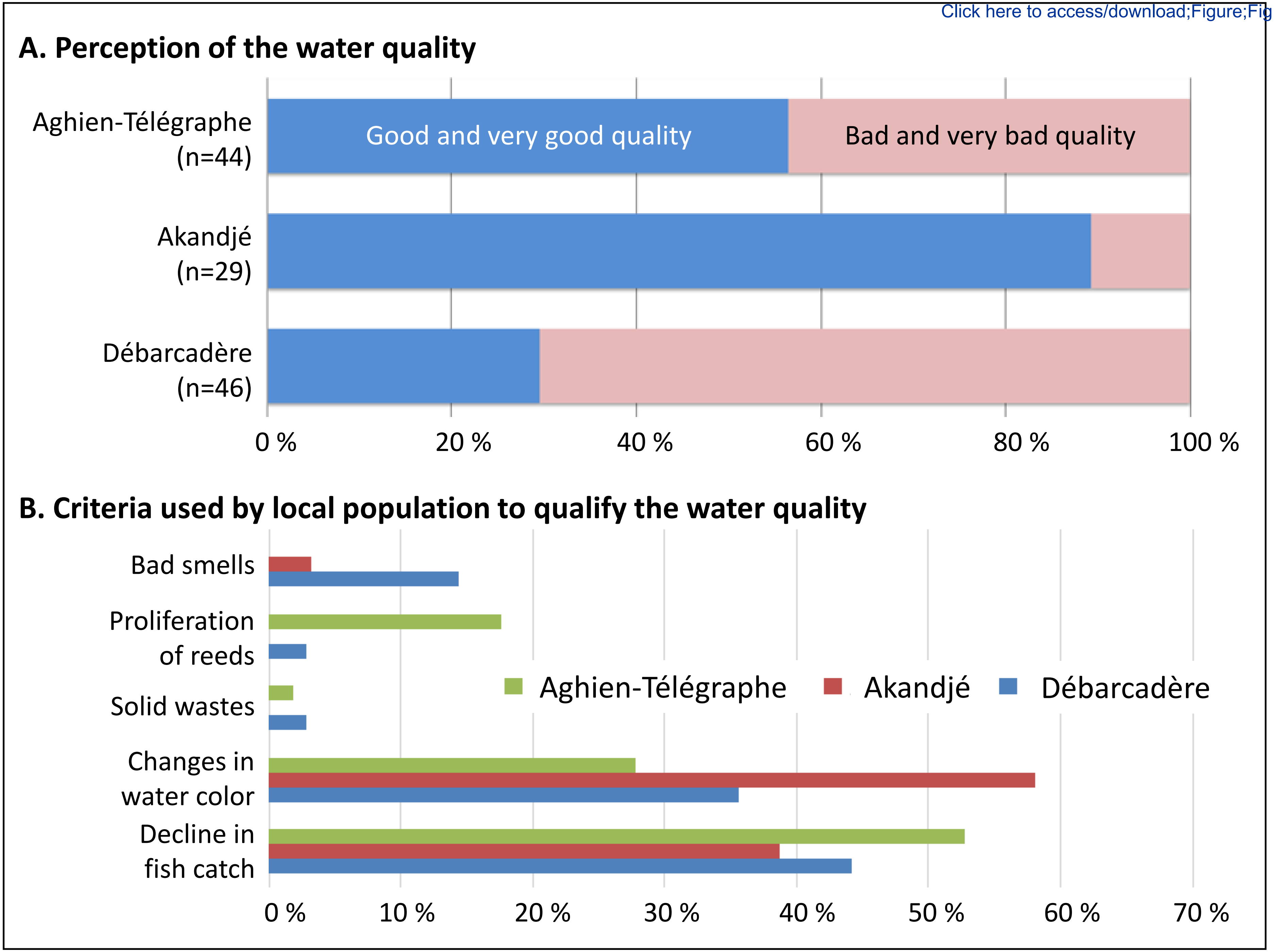

Supporting Table 1

Click here to access/download
Supporting Information
S1 Table.docx

Supporting Fig 1

Click here to access/download
Supporting Information
S1 Fig.pptx

Supporting Fig 2

Click here to access/download
Supporting Information
S2 Fig.pptx

Can participatory approaches strengthen the monitoring of cyanobacterial blooms in developing countries? Results from a pilot study conducted in the Lagoon Aghien (Ivory Coast)

Veronica Mitroi[1], Kouadio Chrislain Ahi[2], Pierre-Yves Bulot[1,3], Fulbert Tra[2], José-Frédéric Deroubaix[4], Mathias Koffi Ahoutou[5,6], Catherine Quiblier[7,8], Mariatou Koné[2], Julien Coulibaly Kalpy[5], Jean-François Humbert[1*]

1. INRAE-Sorbonne Université, iEES Paris, Paris, France
2. Université Félix Houphouët Boigny, Institut d'Ethnosociologie, Abidjan, Côte d'Ivoire
3. INRAE-LISIS, Cité Descartes, Marne-la-Vallée, France
4. LEESU, École des Ponts ParisTech, Champs-sur-Marne, France
5. Institut Pasteur d'Abidjan, Abidjan, Côte d'Ivoire
6. Université Jean Lorougnon Guédé, Daloa, Côte d'Ivoire
7. MNHN, UMR 7245 Molécules de Communication et Adaptation des Micro-organismes, Paris, France
8. Université de Paris, Paris, France

* Corresponding author, jean-francois.humbert@upmc.fr

Field Code Changed

Short Title: Citizen monitoring of cyanobacteria in Africa

Key words: Citizen monitoring, cyanobacteria, Lagoon Aghien, Ivory Coast

Abstract

Monitoring of cyanobacteria in freshwater ecosystems is ~~often complex to organize~~a complex task, which is time consuming and expensive due to the chaotic population dynamics and highly heterogeneous distribution of cyanobacteria populations in water bodies. ~~This~~ The financial cost constitutes a strong limitation for the implementation of long-term monitoring programs in developing countries, particularly in Africa~~, where it is challenging to mobilize sustainable financing for such programs~~. The work presented here was performed in the framework of an international project addressing the sustainable monitoring and management of surface water resources used for the production of drinking water in three African countries. W~~W~~e ~~evaluated~~ tested the potential ~~efficiency~~ of a citizen approach for monitoring cyanobacterial blooms, which are a growing threat to the drinking water supply. This ~~approach~~ pilot study was designed, implemented and evaluated in close interaction with the Pasteur Institute of the Ivory Coast and with the populations of three villages located on the shoreline of a freshwater lagoon located near Abidjan city. Based on the use of a smartphone application, the citizens of the three villages were invited to report water color changes, as these changes could reflect cyanobacteria proliferations. A~~fter a~~ two-year experimentation period ~~d,~~ has shown that it is possible to mobilize the local populations to ~~appeared to provide accurate data on the development~~monitor cyanobacterial blooms. The data collected by citizens were consistent ~~-~~with the data obtained by a classical monitoring of cyanobacteria performed over seven months, but ~~of cyanobacteria in the lagoon. These data were congruent with those obtained by classical monitoring (water sampling and cell counting) performed over seven months~~it appeared that new approaches were needed to validate the citizen data. ~~. In addition, the~~This participatory approach also provided great improvements to the understanding and awareness of local populations regarding water quality and cyanobacterial bloom issues. Finally, we discuss some of the difficulties and limitations of our participatory monitoring approach that should be considered by [illegible]er

implementations. Despite these difficulties, our work suggests that citizen monitoring is a promising approach that may complement the classical approach to sustainable monitoring of cyanobacteria in developing countrie[illegible]

Introduction

Monitoring the proliferations of potentially toxic cyanobacteria in freshwater ecosystems is an essential pillar for the assessment and management of environmental and sanitary risks associated with these phenomena. Numerous studies have therefore been conducted on this issue over the last twenty years. It has been shown that (i) one of the main difficulties in cyanobacteria monitoring is due to the temporal and spatial variability in their vertical and horizontal distributions in water bodies [e.g. 1-3], meaning that the choice of sampling strategy and the tools used for sampling have great impacts on the quality of the data collected [3,4], and (ii) accurate monitoring of cyanobacteria and their associated risks is time consuming and expensive [5].

As a matter of consequence, many efforts have been made to develop and validate new innovative devices for the survey of cyanobacterial blooms. For example, probes equipped with chlorophyll-a/phycocyanin and other sensors that can be coupled with autonomous monitoring platforms are now available for punctual or continuous estimations of cyanobacteria biomasses in the water column [6,7]. In the same way, the use of remote sensing [8] and LIDAR [9] allows ~~us~~ to estimate the horizontal and/or vertical distribution of the cyanobacterial biomass in a lake. Some of these tools remain under development but are increasingly used for cyanobacteria monitoring, particularly in developed countries. ~~On the other hand~~However, ~~in developing countries,~~ the cost of these devices is still a strong limitation to their use in developing countries [10].

Another strategy recently tested for the monitoring of water quality in freshwater ecosystems is the development of citizen science projects based on the involvement of the population living around the targeted freshwater ecosystems [11]. Some of these projects are specifically concerned with the monitoring of cyanobacterial blooms as ~~blooms~~ they are easy to report by nonscientist actors due to changes occurring in the water color or the presence of

colonies or aggregated filaments distinguishable by the naked eye (cf. Fig. 1B). In the USA, the CyanoTRACKER project is based on the use of a smartphone application by volunteers to report algae blooms in their neighborhood pond and/or lake [12]. In the same way, citizen science programs based on the collection of water samples have been implemented for the monitoring of lake water quality and the survey of blooms in the province of Saskatchewan (Canada) [13] and in numerous lakes located in the state of Michigan (USA) [14].

Most of these projects take place in developed countries, fewer citizen programs being available in developing countries [15]. On the African continent, for example, a participatory local observatory has been implemented in Tanzania aiming at collecting and sharing data related to water, rainfall, fisheries, agriculture and food behavior between various stakeholders in water and land management [16]. In South Africa, Nare *et al.* [17] described the difficulties of community involvement and participation in water quality and management in a catchment area due to the inadequate institutional framework. Finally, a good relationship was found between the citizen monitoring of algal blooms and standard laboratory measurements in urban lakes and ponds of three cities located in Brazil and China [18]. These programs highlight the important benefits of this collaboration in terms of both knowledge production, opportunities for democratizing knowledge and expertise under the context of growing environmental and health risks [19].

In the framework of a collaborative project between three African countries (Ivory Coast, Senegal and Uganda) and France (WaSAf project, https://humbert19.wixsite.com/wasaf), In the present paper, we describe performed a first pilot study on the feasibility of setting-up a citizen monitoring program for cyanobacterial bloomsof cyanobacteria in the African context-. based on the use of a smartphone application that was implemented in three villages located around the Lagoon Aghien in the Ivory Coast. This study was performed in the framework of a collaborative project between three African countries

~~(Ivory Coast, Senegal and Uganda) and France (WaSAf project, https://humbert19.wixsite.com/wasaf). The three main goals of this participatory approach were~~ The three main goals of our study were to evaluate (i) the potentials and constraints for the mobilization of the local populations ~~in an African country~~ for the [illegible]al survey of cyanobacteria in aquatic ecosystems, (ii) ~~the efficiency of~~the consistency between the data provided by the citizen monitoring of cyanobacteria ~~by comparing citizen data~~and ~~with data~~ those obtained using a classical ~~sampling~~ approach based on water sampling and cell counting ~~performed by the Pasteur Institute of Abidjan~~ and (iii) the contribution of citizen monitoring to awareness in the neighboring populations regarding the cause and health consequences of cyanobacterial blooms. This citizen monitoring approach was based on the use of a smartphone application and implemented in three villages located around a freshwater lagoon (Lagoon Aghien) in Ivory Coast.

Material & methods

Study site

Lagoon Aghien is a freshwater ecosystem located in the northeastern part of the city of Abidjan (Fig 1A). This lagoon covers a surface area of 19.5 km^2 with a perimeter of 40.72 km and a volume of 25 km^3 [20]. Lagoon Aghien ~~belongs to~~is part of a network of lagoons surrounding the city of Abidjan, with the particularity of being the only one containing freshwater. This lagoon is considered a potential water resource for the city of Abidjan in complement to the water supply currently provided by groundwater extraction. Approximately 12,000 people live in the immediate proximity of the lagoon, mostly distributed in eight villages located on its shoreline (Fig ~~1A~~1B). The lagoon is used by local populations for various domestic (bathing, washing, cooking, waste management, etc.) and economic (fishing, agriculture, etc.) practices that have an impact and are impacted by the water quality of the lagoon. Due to growing human

populations and activities, not only in the immediate environment of the lagoon but also in its whole watershed, coupled with a dramatic lack of sanitation infrastructure and changes occurring in the land use occupation, Lagoon Aghien is now eutrophicated, and cyanobacterial blooms are frequently observed (Fig ~~1B~~1C). Despite the crucial importance of the lagoon for drinking water production, no institutional monitoring of water quality is currently performed.

The villages Débarcadère (3000 people), Akandjé (900 people) and Aghien-Télégraphe (1850 people) were chosen for the implementation of citizen monitoring because of (i) their geographical location enabling satisfactory coverage of the lagoon (Fig 1A), ~~and~~ (ii) the local authorities of the three villages agreed to participate in this experiment and (iii) these villages were representative of the social organization and the various uses of the lagoon for all the villages.

Fig 1. A. Location (red dot) of the lagoon Aghien in Ivory Coast. B. Map of the Lagoon Aghien with the locations of the three villages (Débarcadère, Akandjé, Aghien-Télégraphe) in which citizen monitoring of cyanobacteria was implemented (black circles: villages involved in the citizen monitoring~~Débarcadère, Akandjé, Aghien-Télégraphe)~~; white squares: other villages located around the lagoon). ~~B~~C. Photo of a *Microcystis* sp. (cyanobacteria) bloom in a bay of the Lagoon Aghien.

Stars S1-S6 indicate the sampling points in the monitoring performed by the Pasteur Institute of Abidjan

Ethic statement

This study was not submitted for approval by an institutional review board (ethics committee) because there is currently no committee in Ivory Coast for environmental studies. But as described latter in the paper, we organized in each of the three villages concerned by this study,

a meeting with the chiefdoms in order to present the project and to ask them for their agreement before to begin to work. In addition we asked also to the chiefdoms to designate the sentinels (referent people) in each village who have provided most of the data. Finally, our smartphone application entailed the possibility to collect anonymous reports.

Social ~~engineering~~ investigation for participative water quality monitoring

Sociological investigations conducted in 2017 within the WaSAf program have been used to prepare for the implementation of the participatory monitoring in the three villages. A sociological survey based on a semi-structured questionnaire was performed in all the villages located around the lagoon in order to better understand the different uses of the lagoon and the social organization of the local populations. For the purpose of this pilot study on the citizen monitoring of cyanobacteria, we only used the two questions dealing with the perception of the water quality by the local populations (See S1 Table).

Fig 2. Global overview of the different stages in our approach on the implementation, follow-up and data restitution of citizen monitoring of cyanobacteria in the three villages of the Lagoon Aghien.

Formatted: Indent: First line: 0"

~~In agreement with~~ According to the recommendations of Sturm et al. [21], various actions were performed with local populations with the goal to prepare, conduct and ensure the follow-up and finally report the results of the participatory monitoring in the three villages (Fig 2).

~~**Fig 2.** Global overview of the different stages in our approach on the implementation of citizen monitoring of cyanobacteria in the three villages of the Lagoon Aghien~~

~~As shown by this figure, the participatory monitoring benefited from sociological investigations conducted within the framework of the WaSAf program in the riparian villages to better understand the local practices and uses related to the lagoon and the local social organization.~~ Firstly, m~~M~~eetings and semi-structured interviews were conducted with local community leaders~~ and~~ and chiefdoms, and with inhabitants of the three villages ~~made it possible to better understand their concerns regarding the ecological status of the lagoon and~~in order to discuss about their interest for the implementation of a citizen monitoring ~~the local interest in~~of cyanobacteria~~a monitoring~~. Secondly, f~~After choosing the three relevant villages, f~~ocus groups were ~~also~~ conducted with the more specific objective of identifying the opportunities and constraints for implementing participatory monitoring. ~~In each of the three villages, three different~~These focus groups were organized with homogenous social categories according to gender and age (women, men and youths), including between 7 and 20 participants depending on the groups and the villages. ~~. These focus groups~~They have permitted ~~us~~ to find a common language between scientists and inhabitants, to better define the questionnaire in the mobile application and to generate enthusiasm among the participants to collaborate.

~~Focus groups~~Thirdly, a meeting ~~were~~ was ~~also used~~organized in each village with the goal to present and test ~~mobile~~ the smartphone application ~~with~~ volunteers~~s on mobile phones with the volunteers and explain the use of the application (S1A Fig)~~. Flyers explaining ~~this~~ the use of the application were also distributed to participants to encourage a large participation.

To ensure regular data on the water quality of the lagoon, one person, designated as a sentinel, was chosen in each village by the ~~traditional~~ local chiefdoms. In ~~the first~~Débarcadère village, the sentinel was a school teacher, while in Akandjé village~~the second village~~, he was the representative of the young people. Finally, ~~and~~ in ~~the third village~~Aghien-Télégraphe, the

Formatted: Indent: First line: 0"

Formatted: English (United Kingdom)

chief of the village decided that he could fulfill this taskwas chosen. Each sentinel was equipped with a mobile phone provided by the WaSAf program and was encouraged to take at least one picture per week of the lagoon, always in the same location (chosen together with the research team). The sentinel was also in charge of facilitating the participation of other members of the community willing to participate in the monitoring (helping them to install and use the application, explain the interest in the monitoring, etc.).

Formatted: Indent: First line: 0.49"

S1 Fig. Presentation of the smartphone application (A) and restitution workshop (B) in Débarcadère village (Ivory Coast, shore of Lagoon Aghien).

To maintain the interest of participants and encourage them to continue contributing, we sent SMS messages of thanks to the contributors having registered their phone number in the smartphone application twice during the experiment. In accordance with the project team commitments and to facilitate data appropriation in the villages, two restitution events (based on a poster presentation – S1B Fig) were organized in each village at the end of each year of monitoring. We also prepared two times a ; prospectus (called Lagoon Health Bulletin) presenting together the data collected by citizens and those obtained from the water laboratory analyses performed by the Pasteur Institute of Abidjan. Finally, some educative actions explaining cyanobacterial blooms were performed were also distributed in schools and villagesof Débarcadère and Aghien-Télégraphe villages. These events permitted us to present the comparison between data provided by citizen participation and those obtained from the water laboratory analyses performed by the Pasteur Institute of Abidjan.

AFinally, at the end of the first two years of the pilot study, we also conducted qualitative interviews with the three sentinels and other volunteers who participated in

monitoring to evaluate the effectiveness and difficulties related to participatory monitoring, as well as their appropriation of the monitoring experience. Our questions concerned (i) their participation in the monitoring (how they personally experimented the monitoring process and how this was concretely done, difficulties encountered, etc.), (ii) the impact of this participation on their own perception and understanding of the lagoon's ecological state and (iii) suggestions for improving the monitoring and its sustainability.

The participatory monitoring was organized in two periods: (i) during the first period (from October 2017 to August 2018), the sentinels were asked to provide at least one report per week, and (ii) during the second period (from September 2018 to August 2019), the sentinels were asked to collect and send reports only when something interesting concerning the water quality in general and/or the presence of a cyanobacterial bloom was detected. However, some participants other than sentinels have continued to send reports, even when there was no cyanobacterial bloom. We chose to change the strategy [illegible]e middle of the pilot study in order to determine if the sentinels remained vigilant about cyanobacteria when they no longer had the obligation to send a weekly report.

Smartphone application

The generalized access to mobile phone services endowed with geographic information systems (GIS) creates new opportunities for data collection, access and sharing all over the world [22-24]. According to data from the World Bank [25], access to cellular networks has reached 90 % in numerous African countries, while access to drinking water is only approximately 86% and access to sanitation is close to 60%. Consequently, the use of a smartphone application seemed to be a good tool for involving local communities in participatory monitoring. We a[illegible]d a~~n~~ ~~open source~~free smartphone application that is part of the Epicollect5 data-gathering platform (https://five.epicollect.net/) to the s[illegible]eds of the monitoring. This solution allowed the

Formatted: Not Highlight
Formatted: Not Highlight
Formatted: Not Highlight
Formatted: Not Highlight
Formatted: Not Highlight
Formatted: Not Highlight
Formatted: Not Highlight
Formatted: Not Highlight
Formatted: Not Highlight
Formatted: Not Highlight
Field Code Changed

generation of project-dedicated questionnaires [illegible] the collection of georeferenced data that could be displayed through a web application in the form of maps, tables and charts. For each entry in the WaSAf project (~~S2~~ S1 Fig), users completed a short questionnaire asking for the presence of (i) a green water color and a visible mass of algae suspended in the water, (ii) traces of fuel contamination, (iii) dead fish, (iv) garbage accumulation and/or if there was nothing to report or if they wanted to add other observations. For each questionnaire, users were asked to update their geo-location and to add a picture if they wished. Users can choose to respond anonymously but they could also provide their phone number. This optional information could be used by the project team to ~~reach the users to request more data or~~ provide feedback and to confirm the importance of the collected data (two SMS campaigns were sent). At the end of each questionnaire, users recorded their data and sent them to the website if a data connection was available. If there was no internet connection, the recorded data could be sent later. As soon as a questionnaire was sent, the data were available online. All the data collected in the framework of this project are available at the project website: https://five.epicollect.net/project/wasaf.

Field Code Changed

~~S2~~ S1 Fig. Screenshots of the Epicollect-5 smartphone application with the WaSAf project.

Characteristics of the monitoring of cyanobacteria performed by the Pasteur Institute of Abidjan

~~The~~ In the framework of the WaSAf project, a monitoring of the water quality in the Lagoon Aghien was performed at six sampling stations (S1-S6) from December 2016 to April 2018 by the Pasteur Institute of Abidjan (Fig 1A). [illegible]equently, it was only possible to compare from October 2017 to April 2018, the phytoplankton data obtained by this [illegible]itoring with those resulting from the citizen monitoring of cyanobacteria~~phytoplankton community~~

~~was monitored for seven months by the Pasteur Institute of Abidjan from October 2017 to April 2018 at six sampling stations (S1–S6) (Fig 1A)~~. Data collected on the phytoplankton community [illegible] based on an i~~I~~ntegrated water sampling ~~was~~ performed in the first meter of the water column ~~using an integrated sampler~~ according to the technique used by Laplace-Treyture *et al*. [26]. The counting of cyanobacteria was performed on an inverted microscope (Nikon Eclipse TS100) using the Utermöhl method, according to the AFNOR 15204 standard. A minimum of 30 randomly selected fields was counted to attain at least 500 counting units (cells, colonies or filaments/trichomes).

The results were then expressed as the number of cells per milliliter. In the case of the colonial cyanobacteria *Microcystis* sp., two categories of colonies were considered regarding their diameter (< or > 200 µm). The mean number of cells per category was estimated under an upright microscope by counting the number of cells in 30 colonies per category after they were gently crushed between slides and coverslips. For the filamentous cyanobacteria *Aphanizomenon*, *Cylindrospermopsis*, *Limnothrix*, *Lyngby~~i~~a*, *Oscillatoria*, *Planktothrix*, ~~and~~ *Pseudanabaena* and *Dolichospermum*,~~,~~ the counting considered filament lengths of 100 µm. The mean number of cells per 100 µm of filament was estimated under an upright microscope for 30 filaments for each genus.

The mean cell biovolumes were calculated for the cyanobacteria genera that were most abundant in Lagoon Aghien (*Microcystis*, *Cylindrospermopsis*, *Limnothrix*, *Oscillatoria*, *Dolichospermum*) from measurements performed on 30 cells per genus. For each of the other genera, we used standard cell values defined in the HELCOM PEG Biovolume reports (http://ices.dk/marine-data/vocabularies/Pages/default.aspx).

Formatted: Font: Italic

Results

Overall presentation of the evolution of the number and locations of collected reports during the study period

~~Since the beginning of participatory monitoring, more than 660 questionnaires (January 2020)~~ During the first period of this pilot study (from October 2017 to August 2018), 443 reports have been ~~have been~~ recorded in the WaSAf project hosted by the Epicollect5 platform (see https://five.epicollect.net/project/wasaf). Among these 443 reports, phone numbers were only provided in 37 of them. During the second period of the study (from September 2018 to August 2019), 171 questionnaires were collected and phone numbers were provided in only 7 of them. During the whole study period, it appears that 92.8% of the collected data with the smartphone application were anonymous, which makes it impossible to estimate the number of participants who have contributed to the monitoring.

During the first period of the study, an average of 44 ~~completed questionnaires~~reports was collected each month in the web site of our application, while during the second period, an average of 13 ~~completed questionnaires~~reports were collected monthly (Fig 3). This difference in the number of collected ~~questionnaires~~ reports during the two periods was expected since we changed the recommendation for the sentinels from sending a picture at least once a week (regardless of whether cyanobacteria were observed) to sending pictures only if cyanobacteria or other degradation signs were observed.

Fig 3. N~~Evolution of the n~~umber of completed questionnaires loaded on the WaSAf project Epicollect5 platform. During the first period, the sentinels were asked to complete at least one questionnaire per week with one picture of the lagoon. During the second period, sentinels were advised to send pictures only if cyanobacteria or other worrying phenomena were observed. Dashed line: Monthly average of collected questionnaires during each period.

Formatted: Font: Not Bold

Field Code Changed

Formatted: Font: Not Bold

Formatted: Indent: First line: 0.49"

As shown in Fig 4A, the geographical origins of the data collected during this study were ~~well~~ distributed all around the l~~L~~agoon~~ Aghien. Data were~~ and not only ~~collected~~ near the three villages. Many data points were collected in the western part of the lagoon by the sentinel from the Débarcadère village (Fig 4B). This sentinel performed visual monitoring at four points in this part of the lagoon. Finally, it also appears on the map that some of the data were not located on the shoreline of the lagoon~~, although this phenomenon was probably due to the inaccuracy or misuse of the GPS of the smartphone~~.

Fig 4. D~~Geographical d~~istribution of the ~~data~~ reports collected by citizens around the Lagoon Aghien (A). Detail on the citizen monitoring performed by the sentinel from Débarcadère village (B).

The size of the circles is proportional to the number of reports collected in each place. For the places where more than five reports were collected, the exact number of collected reports is provided into the circle.

Data indicating the potential presence of cyanobacterial blooms

The potential presence of cyanobacterial blooms in the lagoon was reported in the smartphone application by the response “Green water, visible algae” in the questionnaire. These responses were sometimes supported by the pictures associated with the questionnaire ~~presence of a green water color and visible algae in the water~~(see for example two pictures in S2 Fig) but frequently the poor quality of pictures did not permit us to confirm the presence of cyanobacteria. As shown in Fig 5, cyanobacteria blooms were reported mainly during four periods. The first peak of reports occurred in November 2017, followed by a small peak in February 2018, a third peak in May-June 2018 and finally a fourth large peak from May to September 2019.

Fig 5. Evolution of the number of reports made by citizens on the potential presence of cyanobacteria in the Lagoon Aghien (green water color and/or presence of visible colonies)

S2 Fig. Two examples of bloom pictures taken by the participants during the citizen monitoring of the Lagoon Aghien (all the pictures are available on the website (https://five.epicollect.net/project/wasaf)

Formatted: Font: Bold

The geographical locations of the potential algal blooms indicated by the presence of a green water color/visible algae during the three periods were not the same. For the first ~~algal bloom~~ peak ~~-~~(November 2017), almost all reports were from the Débarcadère village, while for the two other peaks (May-June 2018 and May-September 2019), almost all reports of algal blooms were from the Aghien-Télégraphe village.

Comparison of the data provided by the participatory monitoring with those from the Pasteur Institute

Formatted: Font: Italic

It was difficult to compare the data collected by the Pasteur Institute of Abidjan with that collected by the citizens. Indeed, the citizen monitoring was performed on the shores of the lagoon while the monitoring of cyanobacteria by the Pasteur Institute of Abidjan was performed in six sampling points located more than 150 m away from the shores (except for S3). Moreover, water samples were collected by Pasteur Institute once a month while the smartphone reports were sent by citizens as the days went by. For these reasons, we have only compared the monthly number of reports mentioning the presence of a green water color/visible algae with the monthly variations in the total cyanobacterial biovolumes estimated at each sampling stations.

Formatted: Indent: First line: 0"

Two increases in cyanobacterial biovolumes were observed during the monitoring performed by the Pasteur Institute from October 2017 to April 2018. The first increase was recorded in November 2017, and the second one was recorded in February 2018 (Fig 6). Interestingly, during these two increases of cyanobacterial biovolumes, there was also an increase in the number of citizen reports mentioning green water color/visible algae. During the seven months of the Pasteur Institute monitoring, cyanobacteria dominated the phytoplankton community, representing on average 40% (±18) of the total phytoplankton biovolumes estimated at each date and sampling station, when Dinophyta, Chlorophyta, Bacillariophyta and Euglenozoa represented respectively 19% (±14), 17% (±11), 15% (±17) and 9% (±6) of these biovolumes. Finally, t~~T~~he~~se~~ data collected by Pasteur also highlighted that large variations occurred in ~~these~~ cyanobacterial biovolumes depending on the sampling stations, with the largest biovolumes being recorded during this study period at Station 4 and the lowest at Station 6. ~~Finally, as shown in Fig 6, good congruence was found when comparing the data provided by the Institute Pasteur and those provided by citizen monitoring for the same period, suggesting this approach has the potential to contribute to the monitoring of cyanobacteria in Africa.~~

Fig 6. C~~Comparison of the data of c~~yanobacterial biovolumes estimated at the six sampling stations of the Lagoon Aghien ~~collected~~ by the Pasteur Institute and ~~the~~ numb[illegible]f reports ~~on the potential presence of cyanobacteria (~~mentioning a green water color /~~and presence of~~ visible algae ~~in the water)~~ made on the smartphone application by citizens.

CM = Citizen monitoring, S1 to S6 = Sampling stations 1 to 6 ~~for the biovolume estimations of cyanobacteria~~(see Fig 1)

Local understanding of the issues of water quality and cyanobacteria in the lagoon

Formatted: Indent: First line: 0.49"

From the social investigation performed before the implementation of the citizen monitoring, Lagoon Aghien is a very important resource for riparian communities, as it is used for multiple domestic activities (cloth washing, bathing, dishware washing and sometimes cooking) and economic activities (fishing, irrigation, transports, etc.). Consequently, the lagoon has considerable value for thesignificant differences (Chi2 test, p<0.0001) were found in the inhabitants who have mainly a good opinions about the the water quality of the lagoon in the three villages of the lagoon (Fig 7A). Almost 90% of the interviewed people from the Akandjé village and 56% from Aghien-Télégraphe told us that the lagoon exhibits “good and very good quality”. In On the other handthe Débarcadère village, the quality perception was more negative in the Débarcadère village (72% of interviewed people think that the lagoon is in a bad or very bad state) because the village is located between two tributaries that collect urban runoff from Abidjan neighborhoods.

Significant differences were also found between the three villages in the criteria used by local populations to qualify the r[illegible] evolution of the water quality in the lagoon (Chi2 test, p<0.001). These differences mainly concern the proliferation of reeds and the bad smells, which were more specifically associated with the degradation of the water quality in Aghien-Telegraphe and Débarcadèr[illegible]pectively (Fig. 7B). On the other hand, the two most cited criteria used to qualify the recent degradation of the water quality in the three villages When speaking about the evolution of the lagoon quality over time, local people spontaneously refer to the evolution ofwere the fishing captures (a decrease over the last 20 years according to their perception) . However, when asked to explicitly explain the way they perceive the water quality of the lagoon at a given moment and the eventual changes in this quality, the first criterion that is mentioned is and the changes occurring in the water color (Fig 7B). The relative importance of these two criteria varied according to the villages, with significant differences between Akandjé and the two other villages (Chi2 test, p<0.05). The importance of water color for the

Formatted: Indent: First line: 0.49"

~~assessment of water quality by inhabitants was seen by us as a positive signal for the implementation of participative monitoring of cyanobacteria blooms.~~

Fig 7. Perception of the water quality in Lagoon Aghien by residents of the three villages (Aghien-Télégraphe, Akandjé, Débarcadère). Respondents were asked (A) how they evaluate the quality of the lagoon using a four-point scale (very good, good, bad, very bad) and (B) which criteria they use for the assessment of the water quality.

~~The~~ Finally, the focus groups and qualitative interviews have revealed that two main changes in the water color are clearly identified by the local populations~~focus groups and qualitative interviews also provided interesting material to understand the precise implications that are associated with various color changes. Indeed, the participants in the focus groups indicated two main water changes~~: (i) brown water (muddy, reddish) observed mainly during the rainy seasons (from May to July and from September to November), and (ii) green water observed before and/or after the rainy seasons. The brown color was (mainly) associated with "dirty water" coming from the watershed during the rainy periods. During these periods, inhabitants take precautions, and the lagoon is less used for domestic activities to avoid water-related diseases such as diarrhea. The green color of the lagoon was not associated with any sanitary or environmental risks. In contrast, some participants noticed a coincidence between green water color and fish abundance: "*When the water turns green, it smells such as fish, it is fish poop, it is to tell the fishermen that there is a lot of fish and that they can go out fishing*" (Focus group women, Akandjé, Nov. 2017). Only some of the villagers noticed that bathing activities during these green water periods could generate skin and eye irritation. Consequently, no or very few precautions related to domestic uses of water are taken during cyanobacteria blooms. Finally, many participants of the focus groups made a link between the brown color

(the runoff pollution) and the green color (eutrophication), with the first color often preceding the second.

The initial focus groups and the restitution events represented important opportunities for explaining the reasons why researchers and members of the Pasteur Institute were so interested in the monitoring of the “green spots”, as local people called them. On the one hand, local observations are relevant (the phytoplankton biomass production fosters the fish populations), but it has to be stated and explained that a massive development of cyanobacteria also represents a sanitary risk for human activities and can be considered a good indicator of the overall evo[illegible]on of the water quality in the lagoon. On the second hand, such moments of exchange are necessary to motivate the local communities and raise local concern about cyanobacterial blooms, which were previously considered as a natural phenomenon.

Finally, the initial focus groups and the restitution events ~~were “arenas”~~played a role of “hybrid arenas” for the communities and scientists to discuss about the causes and consequences of the degradation of the water quality in the lagoon with scientists. Local chiefdoms were very willing to discuss ~~(i)~~about the contribution to the pollution of the lagoon by populations living close to the lagoon compared to that originating from the rest of the watershed and ~~(ii)~~ the actions and programs that could be implemented to protect/restore the water quality in the lagoon.

Discussion

Although the participation of nonscientists in environmental monitoring is not a recent practice, the phenomenon has undergone spectacular development over the last twenty years, facilitated by new communication technologies and mobile computing applications. This first c[illegible]zen project based on the use of a smartphone application for the monitoring of cyanobacterial blooms in Africa shows that there is an opportunity for such approaches for both (i) the

improvement of the monitoring of cyanobacteria in addition to the monitoring performed by relevant institutions and (ii) the improvement of the awareness and social learnings for the local communities concerning the health risks associated with cyanobacteria and more globally about the degradation of the water quality.

Concerning the improvement of institutional monitoring, as mentioned in the introduction, it is well known that accurate monitoring of cyanobacterial blooms is very costly. This financial cost is a major constraint for the implementation of long-term monitoring in developing countries because of the difficulty of securing long-term funding in these countries. The best illustration of this issue was that the monthly monitoring performed by the Pasteur Institute of Abidjan, at the same time as the participatory monitoring implementation, was stopped ~~after seven months~~ because of the lack of financial resources to continue it. ~~As this~~Our ~~first~~ pilot study has shown that it is possible to mobilize local populations living close to a freshwater ecosystem to monitor it. ~~showed that findings provided by the participatory monitoring were congruent with those provided by the Pasteur Institute of Abidjan~~In this context, it is interesting to raise the issue of the place of this participatory monitoring in an overall monitoring system for the Lagoon Aghien. First, citizen monitoring could be an early warning system for c[illegible]bacterial blooms, which could trigger water samples and analyses. This system could significantly (i) improve the efficiency of institutional monitoring based, for example, on a monthly sampling frequency in a limited number of sampling points and (ii) reduce the cost of institutional monitoring if these two approaches are well coordinated. Second, citizen monitoring coupled with education and capacitation programs could also permit improved basic knowledge of the dynamics~~-~~ and toxicity of cyanobacterial blooms and, more globally, the knowledge of water quality, ~~if it is~~when coupled ~~, for example,~~ with water sampling by untrained and equipped volunteers, as has been done in other participatory projects [13].

Concerning the local awareness and social learnings about cyanobacteria, the focus groups showed that local populations living around the Lagoon Aghien could spontaneously identify visible pollution, as well as changes in physical and biological parameters (such as the water color, macrophytes and algal blooms). At the same time, these focus groups also revealed the low awareness of these populations concerning the health risks associated with cyanobacterial blooms. During the preparation and feedback meetings organized in the three villages, there were also many discussions on the causes of cyanobacterial blooms in freshwater ecosystems. The issue of eutrophication was well understood by most of the participants, with interesting debates on the relative contribution of nutrient discharge into the lagoon from local pollution *versus* "distant pollution" from the other parts of the Abidjan district located in the watershed. These discussions, for example, led the chiefdom representatives of one village to prohibit defecation in the lagoon by the inhabitants of this village and asked for sanitation investments from the regional authorities. Finally, it appears that the social benefits associated with the citizen monitoring approach are both direct (new learning and understanding of water ecosystem functioning and the eutrophication issue and its consequences to cyanobacterial blooms; new capacities to identify pollution sources and sanitary and ecological risks, etc.) and indirect (their appropriation and translation into conservation actions, etc.).

Formatted: Space After: 0 pt

Several limits were also identified during this first ~~approach~~ pilot study on the citizen monitoring of cyanobacteria ~~by citizens~~ in the Ivory Coast. These limits can be qualified as (i) ~~data accuracy – or~~ technical limits; (ii) participatory limits; (iii) data accuracy and validation limits and (iv~~ii~~) institutional limits.

The first technical limits are ~~is~~ related to the accuracy of the produced data.~~, which is~~Some data were affected by the approximate geolocations of several reports, which were not located on the shoreline of the lagoon. These geo-localization errors were~~, which was~~ probably due to the misuse or poor quality of the GPS of some smartphones, knowing ~~. However, it is~~

~~likely~~ that the rapid enhancement of smartphone quality will probably reduce this problem in the ~~future~~ coming years. In the same way, the poor quality of some of the pictures associated with the questionnaires did not permit visual confirmation of the presence of "green algae" indicated in the questionnaire. Again, it is likely that the rapid enhancement of camera quality in smartphones [27] will permit us to solve this problem in the future. Finally, we observed during the meetings that most of the young men and women (<45 years old) had smartphones equipped with a camera, but this was not the case for people >45 years old, who had only small cellphone without a camera. It is likely that the decrease in the prices of smartphones will result in the future, in a more widespread availability of these devices in the populations.

The ~~second~~ participatory limit in ~~the participatory monitoring during this experiment~~our pilot study concerns the fact that despite a very important number of registered data, most ~~data~~ of them were supposed to be sent by the local sentinels. It was ~~not always~~seldom possible to know who sent the reports because ~~the name and the phone number were optionally requested~~few people have provided their phone number in the reports. ~~;~~ H~~h~~owever, when the phone number was provided, it appeared that the sentinels of the three villages sent most of the reports. It is likely that some social/gender categories do not seem to contribute to citizen monitoring such as for example, women, although they are in close contact with the lagoon as they have multiple uses for it. However, from the interviews with the sentinels, it appeared that some villagers who observed changes in the water color asked the sentinels to create a report on the application. Thus, the role of sentinels was very important in the villages, which made the selection of sentinels very important. In our experiment, the best contributor as a sentinel was clearly the schoolteacher from the Débarcadère village, who self-planned to conduct multipoint monitoring. As noted by Requier *et al*. [28], the issue of long-term participation and the improvement of volunteer participation is ~~crucial in developing countries due to the poverty~~

~~of the local population and logistical constraints~~facilitated by key actors (such as sentinels) who can act as intermediaries between scientists and local populations.

We identified several limits in our process of validating data provided by the citizen monitoring. Some congruence was found between these data and that collected by the Pasteur Institute [illegible]his comparison was not fully satisfact[illegible]t would be preferable to perform a monitoring of the water in the same areas where green water color/visible algae were reported by citizens. To achieve this goal, we recommend two different methods for the validation of data collected by citizens. The first one will be based on the use of a smartphone spectrometer by the sentinels in order to evaluate some water quality parameters [29] while the second will consist of water collection and fixation with lugol’s iodine solution, then a microscope examination by scientists. These two methods will be tested in France during the summer 2020 in the framework of a citizen monitoring of cyanobacteria with the same approach used in Ivory Coast.

T~~Finally, t~~he institutional limit in our citizen monitoring approach concerns the integration of the data collected by the citizens within the framework of the institutional monitoring of the lagoon, which is actually missing. The two ministries in charge of the management of water resources and drinking water production have expressed great interest in this participatory approach but failed to see how the collected data could be integrated by their institutions. This result was not because participatory data were considered useless or not accurate enough but because the institutions do not devote the financial and human resources needed for long-term monitoring of freshwater resources. A great deal of awareness-raising among these institutions still needs to be done to show how citizen empowerment might be integrated in the framework of the implementation of long-term monitoring of freshwater

resources. In this goal, we are currently working with the different water institutions of Ivory Coast (Water and Forests Ministry and Hydrology Ministry) for the implementation of an environmental observatory ~~in~~ of the Lagoon Aghien, in which citizen monitoring would ~~have a~~ take its place as an early warning system for cyanobacterial blooms. We hope to reach this objective before the end of the year knowing that citizens have shown their willingness to continue the monitoring of cyanobacteria as shown by the fact that they still send reports while citizen monitoring ended officially one year ago.

~~In conclusion~~Finally, in addition to the design of a smartphone application and the collection of data on cyanobacterial blooms in an African freshwater ecosystem, our citizen monitoring project considered the social aspects of participatory science. These social factors are crucial because they are both a condition for effective participatory monitoring and a condition for maximizing the social utility of the procedure. From this perspective, participatory science is much more than recruitment of volunteers for data collection. The empowerment of knowledgeable citizens, the adoption of critical thinking skills related to the environment and the strengthening of science-society relationship are acknowledged as one crucial point of participatory science projects, even if these points are sometimes marginal in the design of participatory science projects [~~29~~30, ~~30~~31].

In conclusion, this pilot study conducted in Ivory Coast has shown that it is feasible to mobilize local populations in an African country for the monitoring of cyanobacteria. In particular, the choice of having one referent sentinel per village seems to be a good strategy. We also show that this kind of approach, from its initial preparation with local populations to its realization, has improved their awareness on the global issue of water quality. One important issue arising from this study concerns the validation of the data provided by the citizens, which

was not fully achieved. In order to solve this problem, we proposed to test two strategies for the validation of data, which will require collaboration between sentinels and scientists. Finally, the greatest challenge of the citizen monitoring of cyanobacteria but also more generally of all the participatory approaches, concerns their sustainability, knowing that this issue will be handled differently in developed and developing countries. In numerous developed countries where there is already an institutional monitoring of cyanobacteria, the challenge of the sustainability of the citizen monitoring is to show how it can contribute to improve the institutional monitoring of cyanobacteria in order to legitimize its place. For most of the developing countries where there is no existing institutional monitoring, the challenge is to coordinate the joint implementation of these two approaches (institutional and citizen) in order to maximize their efficiency and to minimize the global cost of the monitoring, which is a key point for its sustainability.

Acknowledgments

We are extremely grateful to the three sentinels (Mr. Blaise Lattho in Aghien-Télégraphe, Mr. Kouandjo Guy Rolland Adon in Akandjé, and Mr. Kameleman Siaka Issoufou in Débarcadère) and the inhabitants and chiefdoms of the three villages (Aghien-Télégraphe, Akandjé and Débarcadère) for their decisive contribution to this work. Finally, we would like also to thank the three reviewers of this paper for their constructive comments. This work was supported by the French Facility for Global Environment (Fonds Français pour l'Environnement Mondial, FFEM) (research program WaSAf).

References

1. Welker M, Döhren von H, Täuscher H, Steinberg CEW, Erhard M. Toxic *Microcystis* in shallow lakes Müggelsee (Germany) - dynamics, distribution, diversity. Arch Hydrobiol 2003; 157: 227-248.
2. Cuypers Y, Vinçon-Leité B, Groleau A, Tassin B, Humbert JF. Impact of internal waves on the spatial distribution of *Planktothrix rubescens* (cyanobacteria) in an alpine lake. ISME J 2011; 5: 580-589.
3. Pobel D, Robin J, Humbert JF. Influence of sampling strategies on the monitoring of cyanobacteria in shallow lakes: Lessons from a case study in France. Water Res 2011; 45: 1005-1014.
4. Ahn CY, Joung SH, Park CS, Kim HS, Yoon BD, Oh HM. Comparison of sampling and analytical methods for monitoring of cyanobacteria-dominated surface waters. Hydrobiologia 2008; 596: 413-421.
5. Steffensen DA. Economic cost of cyanobacterial blooms. In: Hudnell H.K. (eds) Cyanobacterial harmful algal blooms: State of the science and research needs. Adv Exp Med Biol 2008; vol 619. Springer, New York, NY
6. Le Vu B, Vinçon-Leite B, Lemaire B, Bensoussan N, Calzas M, Drezen C, et al. 2011. High-frequency monitoring of phytoplankton dynamics within the European water framework directive: application to metalimnetic cyanobacteria. Biogeochemistry 2011; 106: 229-242.
7. Garneau MV, Posch T, Hitz G, Pomerleau F, Pradalier C., Siegwart R, et al. Short-term displacement of *Planktothrix rubescens* (cyanobacteria) in a pre-alpine lake observed using an autonomous sampling platform. Limnol Oceanogr 2013; 58: 1892-1906.

8. Hunter PD, Tyler AN, Willby NJ, Gilvear D.J. The spatial dynamics of vertical migration by *Microcystis aeruginosa* in a eutrophic shallow lake: A case study using high spatial resolution time-series airborne remote sensing. Limnol Oceanogr 2008: 53; 2391-2406.
9. Moore TS, James H, Churnside JH, Sullivan JM, Twardowski MS, Nayak AR, et al. Vertical distributions of blooming cyanobacteria populations in a freshwater lake from LIDAR observations. Remote Sens Env 2019; 225: 347-367.
10. Dube T, Mutanga O, Seutioali K, Adelabu S, Shoko C. Water quality monitoring in sub-Saharan African lakes: a review of remote sensing applications. Afr J Aquat Sci 2015; 40: 1-7.
11. Poisson AC, McCullough IM, Cheruvelil KS, Elliott KC, Latimore JA, Soranno PA. Quantifying the contribution of citizen science to broad-scale ecological databases. Front Ecol Environ 2020; 18: 19-26.
12. ~~Scott MD, Ramaswamy L, Lawson~~Mishra DR, -Kumar A, Ramaswamy L, Boddula VK, Das MC, Page PM, Weber SJ.~~V.~~ CyanoTRACKER: A ~~citizen science project~~cloud-based integrated multi-platform architecture for global observation of cyanobacterial ~~-for reporting~~ harmful algal blooms. ~~Conference: 2nd IEEE International Conference on Collaboration and Internet Computing (IEEE CIC) 2016; 391-397~~Harmful Algae 2020; 96: 101828.
13. Bos JS, Nanayakkara L, Hulbert M, Finlay K. Citizen science for Saskatchewan lakes: a pilot project. Lake Reserv Manage 2019; 35; 77-89.
14. Sarnelle O, Morrison J, Kaul R, Horst G, Wandell H, Bednarz R. Citizen monitoring: Testing hypotheses about the interactive influences of eutrophication and mussel invasion on a cyanobacterial toxin in lakes. Water Res 2010; 44: 141-150.

15. Pocock MJO, Roy HE, August T, Kuria A, Barasa F, Bett J et al. Developing the global potential of citizen science: Assessing opportunities that benefit people, society and the environment in East Africa. J Appl Ecol 2019; 56: 274-281.
16. Duvail S, Paul JL, Hamerlynck O, Majule A, Nyingi WD, Mwakalinga A et al. 2017. Recherches participatives en Tanzanie : un observatoire local pour un dialogue autour de la gestion des territoires et de l'eau. Nat. Sci. Soci. 2017 ; 25 : 347-359.
17. Nare L, Odiyo JO, Francis J, Potgieter N. Framework for effective community participation in water quality management in Luvuvhu catchment of South Africa. Phys Chem Earth 2011; 36: 1063-1070.
18. Castilla EP, Cunha DGF, Lee FWF, Loiselle S, Ho KC, Hall C. Quantification of phytoplankton bloom dynamics by citizen scientists in urban and peri-urban environments. Environ Monit Assess 2015; 187: 690
19. Buytaert W, Zulkafli Z, Grainger S, Acosta L, Alemie TC, Bastiaensen J et al. Citizen science in hydrology and water resources: opportunities for knowledge generation, ecosystem service management, and sustainable development. Front Earth Sci 2014; 2: 51-71.
20. Koffi SE, Koffi TK, Perrin JL, Seguis L, Guilliod M, Goné DL et al. Hydrological and water quality assessment of the Aghien Lagoon hydrosystem (Abidjan, Côte d'Ivoire). Hydrolog Sci J 2019; 64: 1893-1908.
21. Sturm U, Schade S, Ceccaroni L, Gold M, Kyba CMK, Claramunt B et al. Defining principles for mobile apps and plaftorms development in citizen science. RIO 2018; e23394.
22. Silvertown J. A new dawn for citizen science. Trends Ecol Evol 2009; 24: 467-471.
23. Brovelli MA, Minghini M, Zamboni G. Public participation in GIS via mobile applications. ISPRS J Photogramm 2016; 114: 306-315.

24. Mazumdar S, Ceccaroni L, Piera J, Hölker F, Berre AJ, Arlinghaus R et al. Citizen science technologies and new opportunities for participation. In: Hecker, S., Haklay, M., Bowser, A., Makuch, Z., Vogel, J. & Bonn, A. Citizen Science: Innovation in Open Science, Society and Policy. UCL Press, London. 2018; Chap 21: 303-320.

25. World Bank. Water Hackathon - Lesson learned. 2012; Available on https://www.un.org/waterforlifedecade/pdf/2012_world_bank_water_hackathon_lessons.pdf).

26. Laplace-Treyture C, Barbe J, Dutartre A, Druart JC, Rimet F, Anneville O. Protocole standardisé d'échantillonnage, de conservation, d'observation et de dénombrement du phytoplancton en plan d'eau pour la mise en œuvre de la DCE : version 3.3.1. Cemagref, 2009 ; 44 p.

27. Coccia M. Functionality development of product innovation: An empirical analysis of the technological trajectories of smartphone. J Econ Libr 2018; 5:241-258.

28. Requier F, Andersson GKS, Oddi FJ, Garibaldi LA. Citizen science in developing countries: how to improve volunteer participation. Front Ecol Environ 2020; 18: 101-108.

28.29. McGonigle AJS, Wilkes TC, Pering TD, Willmot JR, Cook JM, Mims II FM, Parisi A. Smartphone spectrometers. Sensors 2018; 18:223.

29.30. Dickinson JL, Sirk J, Bonter D, Bonney R, Crain RL, Martin J et al. The current state of citizen science as a tool for ecological research and public engagement. Front Ecol Environ 2012; 10: 291-297.

30.31. Bonney R, Shirk JL, Phillips TB, Wiggins A, Ballard HL, Miller-Rushing A.J. et al. Next steps for citizen science. Science 2014; 343: 1436-1437.

Responses to the reviewers

Reviewer #1: The manuscript submitted to be published as a research article in PlosONE by Humbert et al. presents results on participatory citizen monitoring of algal blooms in the Lagoon Aghien in the Ivory Coast, Africa. The work is done as part of a larger WaSAf project in the area. The objectives of the study were to evaluate the potentials and constraints for the mobilization of the local population around the Lagoon Aghien for the visual monitoring of cyanobacteria/algal occurrence, to compare the results of the cyanobacteria occurrence obtained by the citizen monitoring against the results of phytoplankton sampling and analysis done by the Pasteur Institute of Abidjan (a 7 month overlapping period), and to evaluate the commitment and experiences of local people on the citizen monitoring, and how the process and participation influenced their awareness on cyanobacterial blooms and risks.

The subject of the paper is highly topical as potentially toxic cyanobacterial blooms are a big risk for the use of water in particular in the less developed countries. Therefore, the means to monitor cost-efficiently their occurrence and risks as well as to increase awareness among citizens, water users and water operators is crucial.

The paper is clearly written and easy to follow. The importance of social factors in implementation of this kind of citizen monitoring is well emphasized. Unfortunately, at present the paper is purely descriptive. The authors should use statistical tools to analyze the comparability/congruence between the visual observations and the cyanobacteria biomass results from the selected stations. I appreciate the manuscript is more on describing the process itself, i.e. setting of a participatory monitoring network in a less developed area and to increase awareness in an undeveloped region, but as algal observations and their comparison form an integral part of the paper they should be properly analyzed and presented (see also my later comment to Fig. 6). This comparison could be reasonable to do by analyzing the visual observations from Débarcadère against the station S1 data, from Akandjé against the stations S2 and S3 data, and from Aghien-Télégraphe against the stations S4 and S5 data. This should more reliable show the level of congruence.
As we better explain now in the MS (lines 489-497 on the marked-up version of the manuscript), it was difficult to compare the data provided by the citizen monitoring and those provided by the monitoring performed by the Pasteur Institute because there was the sampling stations were not the same (citizen monitoring was performed on the shores of the lagoon while Pasteur monitoring was performed along a transec located for from the shores (several hundred meters, except for station 3). Moreover, the data acquisition frequency was very different between these two monitoring making also very difficult the data comparison. For these reasons, we

There are similar “quantitative” weaknesses related to the participatory (“socio”) part of the work. For instance, the manuscript lacks detailed statistics; how many people were involved altogether for the monitoring, and what was the number of people who attended the awareness rising gatherings.
We added a Supplemental table (S1 Table) presenting more in details all the sociological approaches performed during this study. This table provides for example the number of questionnaires collected in each village. We also performed some statistical analyses and made changes in the presentation of these data lines 521-581.

In the study, algal bloom situation (cyanobacteria mass occurrence) was monitored as based

on the water color (changes). For the reader it would be important to know, what was the contribution of cyanobacteria of the total phytoplankton biomass during the 7 mo period when the biomass data was available for comparison. This information should be given.
This information is now given lines 502-507.
Further, in lakes algal blooms and water coloration can also result from mass occurrences of other algal groups like chlorophytes, diatoms, euglenoids and occasionally even cryptophytes. How this can be taken into consideration in the risk assessment and interpretation of smartphone photos. This issue needs to be discussed more in the paper. Now it is only briefly referred on page 23.
We added a new paragraph on this issue of the water color and more generally on the issue of the validation of the data provided by the citizen monitoring lines 735-757.

The essential Figure 6 and its legend should be improved and redrawn. The phytoplankton biomass results from different stations (S1-S6) are overlapping (or are they stacked?). For instance, the bloom situation on Nov-17 was observed in the Débarcadère area, where the nearest sampling station for cyanobacteria biomass monitoring was S1. However, in Fig. 6 the biomass from the station S4 hides completely the results of station S1. Also, it should be clearly said in the legend what the citizen observation (CM) bars represent: a monthly average of all observations from the three villages, or something else. Also, it might be more informative to present all data as bars, and including also standard deviation and n of observations? The primary y-axis of Fig. 6 also lacks the unit for biovolumes; please add, and also consider to present them as mg/l which would make the axis more readable.
The figure has been redrawn and the unit for biovolumes has been added. We changed also the legend of the CM axis and the general legend of the figure.

I suggest the authors would also discuss more about the realms of possibility in applying this method to other water bodies and areas in Africa or elsewhere in undeveloped countries. How much external preliminary work, support, expertise and resources are expected to be needed, that was in this study available via the WaSAf project. In this context it would also be important to know about the plans for the continuation of the participatory monitoring in the Lagoon Aghien after the end of the project; how would it succeed in without supporting scientists, smartphones and connections, support etc.
We added a new paragraph at the end of the discussion on the possibility to apply this method elsewhere and also on the need to better validate the data provided by this approach.

Other comments:

Could the results on quantitative phytoplankton analysis for cyanobacteria be related to the WHO cyanobacteria risk levels?
It was not the goal of this paper and in addition, it is not possible to compare the biovolume values that we obtained with the biomass threshold defined in WHO.

Abstract (P8): “These data were congruent with those obtained by classical monitoring (water sampling and cell counting) performed over seven months.” This is strongly stated and requires statistical testing; see my earlier comments.
We have changed this sentence and only speak now about a global congruence between our data and those provided by a classical approach

Abstract (P8): the second goal was “to evaluate efficiency”. To my understanding this was not done. Instead it was evaluated how similar or representative the results from citizen observations are when compared to the quantitative biomass results.
We have changed this sentence (evaluate efficiency) in the abstract and also at the end of the introduction. We agree that our data only reveals the potential of citizen monitoring for the survey of cyanobacteria.

P16: Lyngbia -> Lyngbya
OK, corrected

P16: I suppose Dolichospermum (Anabaena) filaments were also counted as 100 um counting units. Please add to text.
Yes, corrected

P16: The HELCOM PEG biovolumes where used to covert cell numbers into biovolumes. HELCOM biovolumes are meant for the Baltic Sea (brackish water) phytoplankton. Could this have some influence of the biovolume estimates?
We don’t know but as written line 395-397, cell biovolumes of the most dominant genera were estimated after microscopic measurements on 30 cells per genus.

P21 (1st row): …that there opportunity -> that there is an opportunity
OK, corrected

P22: I suppose the method would be suitable for the risk assessment and awareness rising, but not for "traditional" monitoring, because it does not give information on the phytoplankton biomass or species composition.
Yes, we agree with you. As written in our paper, citizen approach for the monitoring of cyanobacteria is interesting when coupling with a traditional approach based on water sampling and cell counting.

P22: “Second, citizen monitoring could also permit improved basic knowledge of the dynamics and toxicity of cyanobacterial blooms…” I don’t see how the tested participatory method would help in monitoring cyanobacterial toxicity, as it is only based on visual observations and smartphone photographs.
We agree that citizen monitoring do not allow alone to provide data on the toxicity but in this sentence we speak about the coupling between visual monitoring and water sampling by volunteers. We changed the sentence to try to makes it more easy to understand.

Reviewer #2: The study reports a pilot study on citizenship science in Africa. It shows the results of a two-year experimentation period collecting data from a limited number of citizens on the development of cyanobacteria in a lagoon in Africa (which provides multiple ecosystem services to citizens) using a smartphone application. The lagoon is used by local populations for various domestic (bathing, washing, cooking, waste management, etc.) and economic (fishing, agriculture, etc.) practices that have an impact and are impacted by the water quality of the lagoon. The highlights and difficulties of this approach are discussed. Noteworthy, most citizen programs happen in developed countries and only few are implemented in developing countries, so I see a great value in this approach, both from the environmental and social point of view. This pilot study showed the low awareness of the

populations surrounding the Lagoon concerning the health risks associated with cyanobacterial blooms. The social benefits associated with the citizen monitoring approach were both direct (new learning and understanding of water ecosystem functioning) and indirect (appropriation and translation into conservation actions).

General Comments

Authors should, throughout the manuscript, show that this is a pilot study, a first phase to improve and scale up this project (in its current version, though it is mentioned in the title, the fact that it is a pilot study is not straightforwardly mentioned throughout the manuscript).
We agree with this remark and mention that this work was a first pilot study several times in the manuscript.

The study proposes three goals, here my comments regarding the goals:

Goal 1. Is well developed in the discussion but should be more detailed in the results sections. For example showing all the responses to the questionnaires.
We provided more details in the result section on the responses to questionnaires (lines 521-631)

Goal 2. Regarding this goal, that tries to relate the data compiled by the citizens with environmental information, the number of occasions where both types of data were available are too low -4 cases- to state that there is agreement. Authors should early in the draft explain why field data area only available for a 7 month period (they do explain it in the discussion but should be explained before). To be able to address this goal, more field data are needed. Authors can use satellite images to explore the possible relation between cyanobacteria abundance (using Normalized difference chlorophyll ratio, for example, or developing local algorithms) and number of questionnaires reported by citizens.
It was not possible to use satellite data in the context of this study. We fully agree that the validation of the data provided by the citizen monitoring is far from satisfactory, even if they seems to be promising. As we describe now in this manuscript, this first study has led us to think about other approaches allowing to better validate the citizen data (see lines 489-497 and lines 735-757). These two approaches will be tested in France during the next summer.

Goal 3. This goal could be considered as a part of Goal 1 for the three villages assessed (I could not find evidence on how other neighboring populations gained awareness from the experience of the studied villages).
As we explained, we discussed several time with local populations and the causes and consequences of cyanobacterial blooms and on the health risks associated with these events. As described lines 685-687, these discussions have led for example the chiefdom representatives of one village to prohibit defecation in the lagoon by the inhabitants of this village and asked for sanitation investments from the regional authorities.

In the introduction I would present first Goal 2 and then Goal 1 as in the results section authors first address Goal 2 and then Goal 1 (or change the order of the results section, describing first the Goal 1).
As we changed the end of our introduction, this comment is no more pertinent.

I understand that this study constitutes a pilot phase of the approach to citizenship monitoring.

The fact that this is a pilot study should be presented in all parts of the manuscript: the abstract, introduction, materials and methods, results and discussion. First I would include in the introduction information about the value and need of pilot phases for installing effective programs of citizen monitoring.
In agreement with our comment, we changed the last paragraph of our introduction and we remember several times in the MS that we present a pilot study.

In the mats and methods section authors need to state that as it is a pilot study only a very limited number of citizens were involved (but that these represented local leaders who had a great respect in their society and a strong disseminating power).
We do not agree with you because it was not possible to know how many different people have contributed to the monitoring because most of the reports were anonymous (see lines 406-419). We assume that sentinels have played an important role but we are not able to quantify their contribution, except for the sentinel in Débarcadère village.

I would like to read in the discussion how authors plan to scale up this approach so as to involve a number of citizenships that can represent the population of the villages, and to bridge the limitations to asses Goal 2 (e.g. use satellite images to check relation between environment and society, satellite imagery can be retrieved from the past also).
We agree with your suggestion and we had two paragraphs in the discussion on the validation of data provided by citizen (see lines 735-757 and 790-811)

Also I would expect in the discussion how would the scaling up of this project would be and which implications it involves (how many people to involve, expectations: for example when more people get involved the number of responses would markedly increase and the patterns of blooms recorded by the citizens would be more representative of what happens in the water, and to press decision makers to take actions. To bridge the current lack of cross talk between government and citizens, a usual problem in developing countries).
We speak now in the discussion about our works with institutions on the implementation of an observatory of the lagoon, in which we expect that the citizen monitoring will be integrated.

Several parts presented in the Results sections should be presented in the Discussion. I marked them with sticky notes throughout the manuscript.
We have taken into account most of your suggestions

See below comments on Figures, also see other comments in the text.

Comments about Figures

Figure 1. add a map of Africa with a location of the studied country, add coordinates in the graph. Also add the other 5 villages that are found near the Lagoon.
Ok, we have changed this figure 1

Figure 2. The results section should provide numbers for these activities. For example. How many reports were answered, how many people participated in the focus groups.
You will find all these data in Table 1. Concerning the number of people participating in the focus groups, it was already provided line 223.

Figure art should be improved, in particular in Fig 3, 5, 6. remove horizontal lines.
Ok we have changed these three figures

Fig. 4. This data correspond to the whole study period? Please clarify in the legend. Put circles with sizes proportional to the number of responses. Add sampling points in this figure, so as to be able to see number of response closest to each sampling point.
As recommended, we have changed this figure and try to clarify the legend (lines 292-293). On the other hand, we do not add the sampling points because they were already presented in Figure 1. Moreover, as already explained in our answer to the reviewer 1, it was not really possible to compare at a spatial scale the data resulting from citizen monitoring and those resulting from the classical monitoring because the citizen monitoring only concerned the shores of the lagoon, while the classical monitoring was performed at least 100 m far from the shoreline.

Fig 5. For the second period (>sept/2018-2019) I would expect that values in Fig 5 should coincide with those in Fig. 3 (as people were advised to send pictures only if cyanobacteria or other worrying phenomena were observed). Why are they different? Please clarify.
Indeed we asked to the sentinels to send picture only when cyanobacterial blooms were observed, but some people other than sentinels have continue to send pictures for example when the water color was brown. That explains that the values for the second period are not the same in Fig 3 and Fig 5.

Fig 6. February instead of Fev. In the legend instead of CM = Citizen monitoring, authors should explain that such monitoring correspond only to potential positive observations, as explained in the secondary axis. I could not find in the text or in the legends how a potential case for cyanobacteria was selected (was it based on the information of discoloration of water, or with the photographs, or with both of them?) This information should be included in the article. Add unit of biovolume in Y axis. Why there is only info up to April 2018? If possible, extend to both periods or at least to the end of the first period, so as to increase the number of situations where there is field data and reports from citizens. Bars of CM should have within them the proportion of locations where the record was performed, village or closest location to the sampling point of cyanobacteria counts (S1 to S6) .
We have change the figure 6 by trying to take into account all the remarks made by the three reviewers on this figure. As explained now in the M&M, it was not possible to extend the period of the institutional monitoring because this monitoring was performed in the framework of a more global monitoring of the lagoon.

Figure 7. The information regarding how this questionnaire was undertaken is absent in the materials and methods section. Please include. Also in the figure state how many people answered this questionnaire.
All these data are now provided in the Table 1 and the number of people answered the questionnaire has been added in Fig. 7A

Supplementary 2. Translate to English the questions asked in the app. Even if the app is French, translate it to English so that the scientific audience can read its content.
OK, a translation in English is now provided (See S1 Fig)

Other comments

All other comments are inserted in the manuscript, due to the lack of line numbers in the manuscript I found it easier to write on the draft directly.

Copy of the comments in the manuscript :
Include in the results section how many meetings were performed, how many people asisted to the meetings before and after the citizenship monitoring program. How many people installed the application.
We included a table (Table 1) providing these data. On the other hand, we don't know how people have installed the application. During the meeting presenting this application, at least five people have installed the application but we know that our sentinels helped people to install it later.

How many people live in the three villages? Out of the 12000 inhabitants surrounding the lagoon i the 8 villages?
We added this data in M&M (Study site), lines 169-174.

In each group or in total?
In each group (corrected, see line 223)

All participants had access to smartphones and electricity?
Yes for the electricity, No for the smartphone but many people have one. We don't know what is the proportion but we provided now some indications in the discussion (lines 715-719).

So records were only performed by the sentinel or also by other members of the village? how many smartphones were used?
It is not possible to have these data because as we described in M&M, people were able to make anonymous reports in the smartphone application. As explained now in lines 308-320, 92.8% of the data collected by the smartphone application were anonymous. But it is likely that a large part of the reports were sent by the sentinels as suggested by the GPS data.

In the results section please say how many applications were installed.
It is not possible to know how many applications were installed but during the meeting presenting this application, the application was installed in 5 to 10 smartphones in each village and we know from the interviews performed after these meetings that other people have installed the application latter.

how many phones were registered?
As previously mentioned, only few smartphone numbers were provided, most of the reports being anonymous.

In the results sections mention how many people attended these meetings.
These data are now available in table 1

Can authors estimate the number of cellphones in the three villages involved?
What we know is that most of the young men and women (<45 years old) have smartphones equipped with a camera, while most of the people >45 years old have only

small cellphone equipped with radio, not with a camera. We added few sentences on that in the M&M (lines 715-719).

Mention that the program run out fo funds. At first when I did not know this information, until I read it in the Discussion, i thought why such a short time frame was selected.
We better explain now why we only get data on phytoplankton community during seven months (lines 363-368).

How many cellphones where involved. The question is if the three sentinels produced all questionnaries or if many citizens were involved in completing the questionaries. Only in the discussion i understood that most questionaries were done by three persons.
Again, due to the anonymous option for the smartphone application, it is not possible to know the relative contribution of the sentinels and of other people in the citizen monitoring. But regarding to the GPS location, we estimated that a large part of the contributions were done by the sentinels, in particular for the Débarcadère village.

This sentence (This difference in the number of collected questionnaires during the two periods was expected since we changed there commendation for the sentinels from sending a picture at least once a week…) should be in discussion, not here in results.
We think that it is better not to change the place of this sentence for the overall understanding of this paragraph.

remove well.
Ok, removed…

Add full stop.
Ok, added

This part of the sentence should be located in Discussion not in Results.
Ok, this part of the sentence has been removed and we added one sentence on this issue in the discussion

Looking into the data of the application the differences in color can be due to cyanobacteria or due to organic matter. How could you differentiate one from the other?
The color of the water during cyanobacterial blooms is different from the color of water with high amount of organic matter, much more green during the blooms. Moreover, during cyanobacterial blooms it is very likely to see in water some pellets corresponding for example to Microcystis colonies. If you look at supplemental Figure 2, it was clearly visible. But we agree with you that sometimes, you can have a green color in the water due to other phytoplankton microalgae than cyanobacteria.

this village is closest to S1, but in the graph the bloom is largest in S4 which is more close to Aghien T. village. I would expect a discussion about this in the discussion section.
It was difficult to really compare the data providing by the citizen monitoring and those resulting from the classical approach performed by the Pasteur Institute. The two main reasons are that (i) it is well known that you can observed accumulation of cyanobacteria on the shore of the lake but quite nothing 50 m away of this shore and (ii) the spatial distribution of the cyanobacterial blooms can change very quickly (few hours) due to wind and current effects. For a definitive validation of the data provided by the citizen monitoring, it should have been necessary to sample water at the places

where the pictures were taken. Knowing that, we have choose to limit the comparison between these data in term of co-occurrence between the monthly reports of cyanobacteria by citizens and the total biovolumes of cyanobacteria estimated at the end of each month by the Pasteur Institute. You can see all the changes that have been made in the result and discussion parts concerning this issue (lines....).

Remove "Evolution of the", start with Number
Ok, we changed the legend of this figure.

This “suggesting this approach has the potential to contribute to the monitoring of cyanobacteria inAfrica” should go in discussion not in results.
Ok we removed the end of the sentence

the first two sentences should go in discussion.
Ok, they have been removed

There is no information in materials and methods regarding this questionary.
As explain now in the M&M, we used data from a large questionnaire performed in the framework of researches in social sciences on the populations living around the lagoon. We only used two questions, which are now provided in supplemental data (see S1 Table)

S1 from Fig. 6 seems of better quality than S4. Mention that despite marked differences in teh water quality the perception per village shows an opposite trend.
In fact, when looking on the whole data set collected from December 2016 to April 2018 (a publication is in preparation on these data set which concerns many physico-chemical and biological parameters), we show that in agreement with the perception of people living in Débarcadère village, the water quality is worse in this area than in the rest of the lake.

Add a reference to Fig. 7b for fish
There is no reference for fish. The decrease in fish catch is experienced by the local fishermen.

rephrase this sentence. as seen in fig. 7b. both color of water and decreased fishe catch are seen as very important issues in the lagoon
Yes, we agree with you. The sentence has been changed.

This sentence (The focus groups and qualitative interviews also provided…) should be relocated into the discussion.
We do not agree with you because the interpretation of the changes occurring in the water color is also a result.

This paragraph (The initial focus groups and the restitution events represented…) should be relocated in to the discussion.
Again we do not agree with you because we provided new data in this paragraph that results directly from the focus group and qualitative interviews.

this information should be presented in materials and methods, when explaining the available data.

Yes, we explained now in the M&M how these data were obtained

The use of satellite images can help substantially in the monitoring of the Lagoon. Once implemented the cost is very low and can be used to assess the role of citizenship monitoring and the detection of blooms.
We agree that the use of satellite images can be useful for the monitoring of cyanobacteria but in the local context, it seems difficult to imagine to use this kind of approach due to the strong limitation of financial resources.

did not show, number was too low. change the words, use words such as seem, suggest, not show. here authors need to aknowledge that the number of data they have are too low. please rewrite this sentece. i can see the value of citizen monitoring which has no cost. put it in a separate sentence and elaborate longer.
We have changed this paragraph in our discussion

coupled with education and capacitation programs. Please include.
Ok, it is now included

remove extra space
Ok, done

This information should be mentioned before in the manuscript, for example in Results and show examples of photographs, at least in supplementary material. I checked on the web page and by looking at the photos alone the color of the water. **Yes we agree that the quality of numerous pictures was to low to really see the color of the water event if sometimes the green color and the cyanobacteria were clearly visible for some of them (see S2 Fig). We added a sentence in the result section (lines 459-463)**

This information should be mentioned before, in the results section. How many smartphones participated in gathering the information. If 90% of people have access to cellphones, as stated somewhere in the draft, then if only 3 chiefs produce the data, then the participation of the citizens is low.
We do not know how many people and how many smartphone have contributed to the monitoring because most of the report were anonymous (see our previous answer on this question).

How many people in the villages had access to cellphones? How many women use cellphones?
We already provided an answer to this question (see above)

This information should be presented in the REsults section as part of the results of the focus group meetings
We do not understand why this information should be presented in the result section. We discussed in this paragraph the fact that depending on the choice of this sentinel (which was made by the chiefdom), the investment made by each sentinel was very various, showing the importance of this choice.

Here you can suggest to identify good teachers in each village to take the leading role (sentinels) but also as the role of disseminating the importance and results to students and to their parents (either directly or throught their kids).

It is more complicated than that. It was not possible for us to choose the sentinels in the villages because this is clearly a responsibility of the chiefdom. That explains why we have so different people as sentinel in the three villages.

Reviewer #3: Author's designed, implemented and evaluated a project of participatory monitoring of cyanobacteria blooms through the use of a smartphone application, working with the populations of three villages located on the shoreline of a freshwater lagoon located near Abidjan city (Lagoon Aghien in the Ivory Coast). The aim was to analyze the efficiency of such citizen science approach, comparing with classical monitoring, the potential constraints of the approach, and the contribution to citizen awareness about cyanobacteria blooms and their health consequences. Author's claimed that the participatory approach provided accurate data compared with classical monitoring, and in addition, showed great improvements to the understanding and awareness of local populations regarding water quality issues.

In my opinion, the work is interesting, although the limited data of the classical monitoring make it less relevant than it could have been. It's a shame that the classical monitoring was unilaterally finished by French institutions. In my personal opinion, there is limited data to account for a solid comparison. In this sense, in my opinion the work is quite limited about the efficacy of the participatory detection of cyanobacteria blooms.
The classical monitoring was not stopped unilaterally by French institutions (see our response below). We fully agree that it was not possible to perform a solid comparison between citizen monitoring and institutional monitoring but our paper show that it is really possible to involve people in Africa in the monitoring of freshwater ecosystems, to obtain potentially promising findings on their ability to detect cyanobacterial blooms and finally that this kind of approach is a powerful tool to increase the awareness of local populations to the issue of water quality.

Globally, my recommendation is to consider some major and minor revisions, some of them are important to clarify or improve the manuscript.

Majors

1. The comparison of the participatory approach with the classical one shows some caveats that don't totally reflect the efficacy of the participatory approach about the detection of cyanobacteria blooms. For example, it appears to be a threshold in the detection of water color changes associated with cyanobacteria blooms. Some smaller blooms were not detected by local people. This issue have to be more addressed on the text.
We agree with your comment and we made changes both in the "result" and "discussion" sections

2. "The importance of water color for the assessment of water quality by inhabitants was seen by us as a positive signal for the implementation of participative monitoring of cyanobacteria blooms." Results section.
I don't agree with that. A limitation is that before smartphone application you don't know if they use water color as indicator of water quality. That's a limitation to inform about the awareness associated with monitoring. Please remove or modify accordingly if you have more information previous to the beginning of participatory monitoring.

We do not understand this remark because the responses of people to our questionnaire has shown that they clearly associated changes in water quality with the water color (see lines 588-589). In particular brown and green water were clearly identified as particular periods.

3. I think that Figure 6 and the comparative analysis of monitoring programs should be improved. It is desirable to have some quantitative measurement of agreement between the classical and the participatory approaches. I suggest some kind of analysis that allow subjective/objective comparisons (e.g. ROC curves), or some kind of association/classification analysis.
We have made changes in Fig 6. As we explained now in the MS, due to spatial and temporal differences in the strategy of the two monitoring approaches, it is difficult to compare the data they provided. For this reason, we can just check as we did in Fig 6, if there was some consistency between these data.

Meanwhile, the plot is not very straightforward to understand it, in particular regarding biovolume counts in each station. Related with this, S5 and S6 have the same color? Color codes could also be improved to facilitate identification of stations. Lack of units in y-axis.
We have changed the figure

Minors

Material and Methods sectioned,

4. "As shown by this figure, the participatory monitoring benefited from sociological investigations conducted within the framework of the WaSAf program…".
This is not clear in Figure 2. Where is mentioned in Fig. 2 the previous sociological research? Meetings are not only part of sociological research, they can be part of any research. Please modify the text or the Figure accordingly.
We agree that it was not enough clear and we have changed this paragraph in M&M

5. Figure 2. It is not clear from the figure or the text why the steps are divided in three blocks without a correspondence with the three main titles of the boxes. Why is it? I think that it is confusing, I suggest to edit the figure or to be clearer with that at the legend. For example, Phase 1, preparation, have 2 items and a previous one: 1) first meeting to choice villages, 2) organization of focus groups, 3) meetings and provision of flyers of smartphone application; but the phase was divided in two boxes, the first two items in a box, and the third item on a second box. Why? The same thing with the rest of the phases/boxes.
Ok, we changed this picture in order to make it more clear and logical

6. "Meetings and interviews with local community leaders and chiefdom and with inhabitants of the three villages.."
Please check syntaxes. A comma is lacking somewhere
OK, corrected

7. "prospectus (Lagoon Health Bulletin) were also distributed in schools and villages."
Prospectus were done by scientist or also by local actors, such as sentinels? Please clarify.
OK, it is now clarified

8. " we also conducted qualitative interviews with the three sentinels and other volunteers".

What kind of interview did author's performed? Informal? Semi-structured? Please clarify the kind of interview and any further details to understand the kind of responses given by interviewers.
OK, we provide now more details on these methodologies

9. “The participatory monitoring was organized in two periods…”
Why did authors choose this approach? It was top-down (scientist to local people) or it was discussed or decided with local actors? Can you clarify the approach?
Yes, we provided now more detailed on this strategy lines 321-329

Results section

10. What are the violet points in Figure 4? Please clarify or remove them.
Also in agreement with the first reviewer, we changed this figure and its legend in order to make them more easy to understand

11. Figure 5. To have a better picture of the participatory monitoring of blooms, authors can include within the graph several photos sent by sentinels corresponding to different moments of the monitoring dynamics.
Unfortunately, it is not possible to download the pictures with a sufficient definition for a publication in the paper. Each image has a definition of 140 dpi while 300-600 dpi has required for PLoS ONE. All the pictures are still available on the web site and we added a Supplemental Figure (S2 Fig) presenting two images of blooms taken by the sentinels.

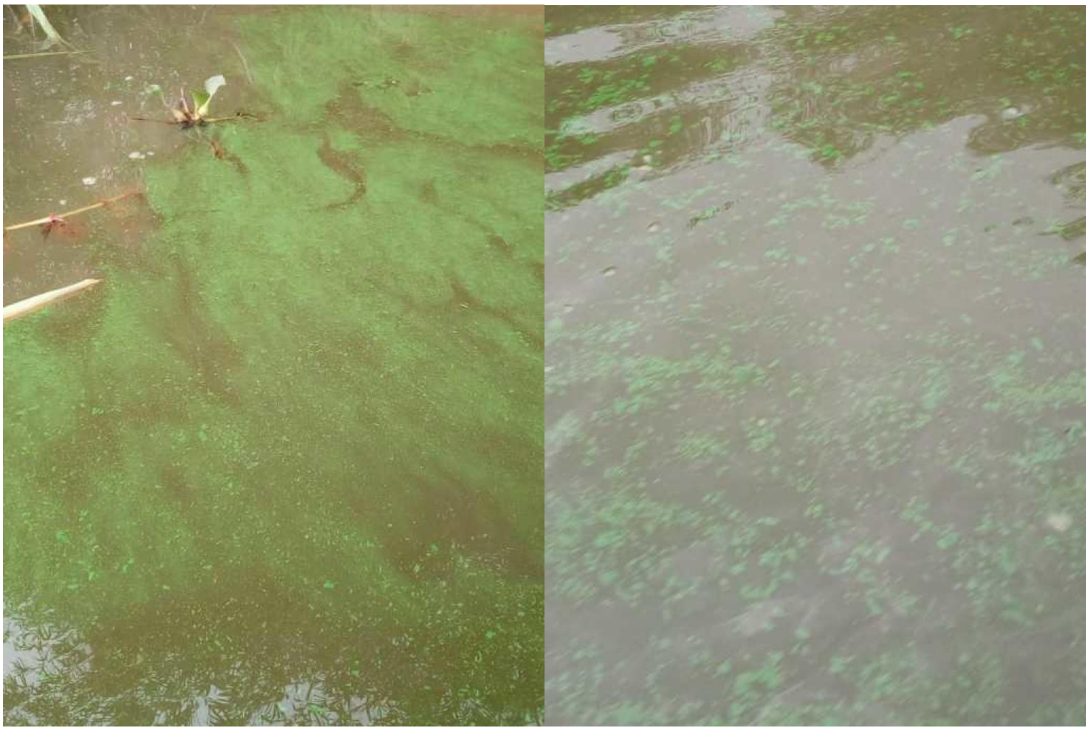

Discussion section

12. “This first citizen project based on the use of a smartphone application for the monitoring

of cyanobacterial blooms in Africa shows that there opportunity for such approaches.”
Did authors mean “there is opportunity”? Please correct.
Yes, we have corrected this sentence

13. Limitations of the program. Authors have limited data to take conclusions about participatory monitoring. Some blooms were not recorded by participants, is not just technical problems regarding geolocalization. This is an important issue and has implications for future monitoring programs.
We agree that this pilot study only provided first indications on the potential of a citizen monitoring for the survey of cyanobacteria. That’s the reason why we added a new paragraph at the end of the discussion and also that we moderate our purposes on the efficiency of this monitoring in result and discussion sections.

14. What was the reaction of local people when French institutions unilaterally finished the monitoring? May be it would be interesting to mention at the discussion section.
We did not write that French institutions unilaterally stopped the monitoring. The Pasteur Institute of Abidjan had got funds for a 18 month monitoring of the lagoon which ended in April 2018. It was not possible to find new funds from Ivory Coast institutions in order to continue this institutional monitoring.

15. Have sentinels stopped to send reports so far? Which is the long-term sustainability of the program? If author's can expand this observation it would be great.
No, two of them continue to send pictures, even if the program is at the end. But we are working on the implementation of an observatory of the lagoon Aghien with the goal to to perform a sustainable monitoring on the lagoon, including classical and participatory approaches. Hope that it will be possible to do in the next months!

16. I fully agree with the content of last paragraph but I think this is not a conclusion technically speaking. I suggest that in order to maintain the content in the text, please modify the paragraph and/or add a conclusion relative with the goals of the work.
OK, we added a new conclusion at the end of the paper.
