# Supplementary material for: Can participatory approaches strengthen the monitoring of cyanobacterial blooms in developing countries? Results from a pilot study conducted in the Lagoon Aghien (Ivory Coast)
Source: PLoS One. 2020 Sep 24;15(9):e0238832. doi: 10.1371/journal.pone.0238832 (PMC7514105; doi:10.1371/journal.pone.0238832)
Supplement: Attachment — Submitted filename: Responses to the reviewers.docx [file pone.0238832.s005.docx]

**Responses to the reviewers**

Reviewer #1: The manuscript submitted to be published as a research article in PlosONE by Humbert et al. presents results on participatory citizen monitoring of algal blooms in the Lagoon Aghien in the Ivory Coast, Africa. The work is done as part of a larger WaSAf project in the area. The objectives of the study were to evaluate the potentials and constraints for the mobilization of the local population around the Lagoon Aghien for the visual monitoring of cyanobacteria/algal occurrence, to compare the results of the cyanobacteria occurrence obtained by the citizen monitoring against the results of phytoplankton sampling and analysis done by the Pasteur Institute of Abidjan (a 7 month overlapping period), and to evaluate the commitment and experiences of local people on the citizen monitoring, and how the process and participation influenced their awareness on cyanobacterial blooms and risks.

The subject of the paper is highly topical as potentially toxic cyanobacterial blooms are a big risk for the use of water in particular in the less developed countries. Therefore, the means to monitor cost-efficiently their occurrence and risks as well as to increase awareness among citizens, water users and water operators is crucial.

The paper is clearly written and easy to follow. The importance of social factors in implementation of this kind of citizen monitoring is well emphasized. Unfortunately, at present the paper is purely descriptive. The authors should use statistical tools to analyze the comparability/congruence between the visual observations and the cyanobacteria biomass results from the selected stations. I appreciate the manuscript is more on describing the process itself, i.e. setting of a participatory monitoring network in a less developed area and to increase awareness in an undeveloped region, but as algal observations and their comparison form an integral part of the paper they should be properly analyzed and presented (see also my later comment to Fig. 6). This comparison could be reasonable to do by analyzing the visual observations from Débarcadère against the station S1 data, from Akandjé against the stations S2 and S3 data, and from Aghien-Télégraphe against the stations S4 and S5 data. This should more reliable show the level of congruence.

**As we better explain now in the MS (lines 489-497 on the marked-up version of the manuscript), it was difficult to compare the data provided by the citizen monitoring and those provided by the monitoring performed by the Pasteur Institute because there was the sampling stations were not the same (citizen monitoring was performed on the shores of the lagoon while Pasteur monitoring was performed along a transec located for from the shores (several hundred meters, except for station 3). Moreover, the data acquisition frequency was very different between these two monitoring making also very difficult the data comparison. For these reasons, we**

There are similar “quantitative” weaknesses related to the participatory (“socio”) part of the work. For instance, the manuscript lacks detailed statistics; how many people were involved altogether for the monitoring, and what was the number of people who attended the awareness rising gatherings.

**We added a Supplemental table (S1 Table) presenting more in details all the sociological approaches performed during this study. This table provides for example the number of questionnaires collected in each village. We also performed some statistical analyses and made changes in the presentation of these data lines 521-581.**

In the study, algal bloom situation (cyanobacteria mass occurrence) was monitored as based on the water color (changes). For the reader it would be important to know, what was the contribution of cyanobacteria of the total phytoplankton biomass during the 7 mo period when the biomass data was available for comparison. This information should be given.

**This information is now given lines 502-507.**

Further, in lakes algal blooms and water coloration can also result from mass occurrences of other algal groups like chlorophytes, diatoms, euglenoids and occasionally even cryptophytes. How this can be taken into consideration in the risk assessment and interpretation of smartphone photos. This issue needs to be discussed more in the paper. Now it is only briefly referred on page 23.

**We added a new paragraph on this issue of the water color and more generally on the issue of the validation of the data provided by the citizen monitoring lines 735-757.**

The essential Figure 6 and its legend should be improved and redrawn. The phytoplankton biomass results from different stations (S1-S6) are overlapping (or are they stacked?). For instance, the bloom situation on Nov-17 was observed in the Débarcadère area, where the nearest sampling station for cyanobacteria biomass monitoring was S1. However, in Fig. 6 the biomass from the station S4 hides completely the results of station S1. Also, it should be clearly said in the legend what the citizen observation (CM) bars represent: a monthly average of all observations from the three villages, or something else. Also, it might be more informative to present all data as bars, and including also standard deviation and n of observations? The primary y-axis of Fig. 6 also lacks the unit for biovolumes; please add, and also consider to present them as mg/l which would make the axis more readable.

**The figure has been redrawn and the unit for biovolumes has been added. We changed also the legend of the CM axis and the general legend of the figure.**

I suggest the authors would also discuss more about the realms of possibility in applying this method to other water bodies and areas in Africa or elsewhere in undeveloped countries. How much external preliminary work, support, expertise and resources are expected to be needed, that was in this study available via the WaSAf project. In this context it would also be important to know about the plans for the continuation of the participatory monitoring in the Lagoon Aghien after the end of the project; how would it succeed in without supporting scientists, smartphones and connections, support etc.

**We added a new paragraph at the end of the discussion on the possibility to apply this method elsewhere and also on the need to better validate the data provided by this approach.**

Other comments:

Could the results on quantitative phytoplankton analysis for cyanobacteria be related to the WHO cyanobacteria risk levels?

**It was not the goal of this paper and in addition, it is not possible to compare the biovolume values that we obtained with the biomass threshold defined in WHO.**

Abstract (P8): “These data were congruent with those obtained by classical monitoring (water sampling and cell counting) performed over seven months.” This is strongly stated and requires statistical testing; see my earlier comments.

**We have changed this sentence and only speak now about a global congruence between our data and those provided by a classical approach**

Abstract (P8): the second goal was “to evaluate efficiency”. To my understanding this was not done. Instead it was evaluated how similar or representative the results from citizen observations are when compared to the quantitative biomass results.

**We have changed this sentence (evaluate efficiency) in the abstract and also at the end of the introduction**. **We agree that our data only reveals the potential of citizen monitoring for the survey of cyanobacteria.**

P16: Lyngbia -> Lyngbya

**OK, corrected**

P16: I suppose Dolichospermum (Anabaena) filaments were also counted as 100 um counting units. Please add to text.

**Yes, corrected**

P16: The HELCOM PEG biovolumes where used to covert cell numbers into biovolumes. HELCOM biovolumes are meant for the Baltic Sea (brackish water) phytoplankton. Could this have some influence of the biovolume estimates?

**We don’t know but as written line 395-397, cell biovolumes of the most dominant genera were estimated after microscopic measurements on 30 cells per genus.**

P21 (1st row): …that there opportunity -> that there is an opportunity

**OK, corrected**

P22: I suppose the method would be suitable for the risk assessment and awareness rising, but not for "traditional" monitoring, because it does not give information on the phytoplankton biomass or species composition.

**Yes, we agree with you. As written in our paper, citizen approach for the monitoring of cyanobacteria is interesting when coupling with a traditional approach based on water sampling and cell counting.**

P22: “Second, citizen monitoring could also permit improved basic knowledge of the dynamics and toxicity of cyanobacterial blooms…” I don’t see how the tested participatory method would help in monitoring cyanobacterial toxicity, as it is only based on visual observations and smartphone photographs.

**We agree that citizen monitoring do not allow alone to provide data on the toxicity but in this sentence we speak about the coupling between visual monitoring and water sampling by volunteers. We changed the sentence to try to makes it more easy to understand.**

Reviewer #2: The study reports a pilot study on citizenship science in Africa. It shows the results of a two-year experimentation period collecting data from a limited number of citizens on the development of cyanobacteria in a lagoon in Africa (which provides multiple ecosystem services to citizens) using a smartphone application. The lagoon is used by local populations for various domestic (bathing, washing, cooking, waste management, etc.) and economic (fishing, agriculture, etc.) practices that have an impact and are impacted by the water quality of the lagoon. The highlights and difficulties of this approach are discussed. Noteworthy, most citizen programs happen in developed countries and only few are implemented in developing countries, so I see a great value in this approach, both from the environmental and social point of view. This pilot study showed the low awareness of the populations surrounding the Lagoon concerning the health risks associated with cyanobacterial blooms. The social benefits associated with the citizen monitoring approach were both direct (new learning and understanding of water ecosystem functioning) and indirect (appropriation and translation into conservation actions).

General Comments

Authors should, throughout the manuscript, show that this is a pilot study, a first phase to improve and scale up this project (in its current version, though it is mentioned in the title, the fact that it is a pilot study is not straightforwardly mentioned throughout the manuscript).

**We agree with this remark and mention that this work was a first pilot study several times in the manuscript.**

The study proposes three goals, here my comments regarding the goals:

Goal 1. Is well developed in the discussion but should be more detailed in the results sections. For example showing all the responses to the questionnaires.

**We provided more details in the result section on the responses to questionnaires (lines 521-631)**

Goal 2. Regarding this goal, that tries to relate the data compiled by the citizens with environmental information, the number of occasions where both types of data were available are too low -4 cases- to state that there is agreement. Authors should early in the draft explain why field data area only available for a 7 month period (they do explain it in the discussion but should be explained before). To be able to address this goal, more field data are needed. Authors can use satellite images to explore the possible relation between cyanobacteria abundance (using Normalized difference chlorophyll ratio, for example, or developing local algorithms) and number of questionnaires reported by citizens.

**It was not possible to use satellite data in the context of this study. We fully agree that the validation of the data provided by the citizen monitoring is far from satisfactory, even if they seems to be promising. As we describe now in this manuscript, this first study has led us to think about other approaches allowing to better validate the citizen data (see lines 489-497 and lines 735-757). These two approaches will be tested in France during the next summer.**

Goal 3. This goal could be considered as a part of Goal 1 for the three villages assessed (I could not find evidence on how other neighboring populations gained awareness from the experience of the studied villages).

**As we explained, we discussed several time with local populations and the causes and consequences of cyanobacterial blooms and on the health risks associated with these events. As described lines 685-687, these discussions have led for example the chiefdom representatives of one village to prohibit defecation in the lagoon by the inhabitants of this village and asked for sanitation investments from the regional authorities.**

In the introduction I would present first Goal 2 and then Goal 1 as in the results section authors first address Goal 2 and then Goal 1 (or change the order of the results section, describing first the Goal 1).

**As we changed the end of our introduction, this comment is no more pertinent.**

I understand that this study constitutes a pilot phase of the approach to citizenship monitoring. The fact that this is a pilot study should be presented in all parts of the manuscript: the abstract, introduction, materials and methods, results and discussion. First I would include in the introduction information about the value and need of pilot phases for installing effective programs of citizen monitoring.

**In agreement with our comment, we changed the last paragraph of our introduction and we remember several times in the MS that we present a pilot study.**

In the mats and methods section authors need to state that as it is a pilot study only a very limited number of citizens were involved (but that these represented local leaders who had a great respect in their society and a strong disseminating power).

**We do not agree with you because it was not possible to know how many different people have contributed to the monitoring because most of the reports were anonymous (see lines 406-419). We assume that sentinels have played an important role but we are not able to quantify their contribution, except for the sentinel in Débarcadère village.**

I would like to read in the discussion how authors plan to scale up this approach so as to involve a number of citizenships that can represent the population of the villages, and to bridge the limitations to asses Goal 2 (e.g. use satellite images to check relation between environment and society, satellite imagery can be retrieved from the past also).

**We agree with your suggestion and we had two paragraphs in the discussion on the validation of data provided by citizen (see lines 735-757 and 790-811)**

Also I would expect in the discussion how would the scaling up of this project would be and which implications it involves (how many people to involve, expectations: for example when more people get involved the number of responses would markedly increase and the patterns of blooms recorded by the citizens would be more representative of what happens in the water, and to press decision makers to take actions. To bridge the current lack of cross talk between government and citizens, a usual problem in developing countries).

**We speak now in the discussion about our works with institutions on the implementation of an observatory of the lagoon, in which we expect that the citizen monitoring will be integrated.**

Several parts presented in the Results sections should be presented in the Discussion. I marked them with sticky notes throughout the manuscript.

**We have taken into account most of your suggestions**

See below comments on Figures, also see other comments in the text.

Comments about Figures

Figure 1. add a map of Africa with a location of the studied country, add coordinates in the graph. Also add the other 5 villages that are found near the Lagoon.

**Ok, we have changed this figure 1**

Figure 2. The results section should provide numbers for these activities. For example. How many reports were answered, how many people participated in the focus groups.

**You will find all these data in Table 1. Concerning the number of people participating in the focus groups, it was already provided line 223.**

Figure art should be improved, in particular in Fig 3, 5, 6. remove horizontal lines.

**Ok we have changed these three figures**

Fig. 4. This data correspond to the whole study period? Please clarify in the legend. Put circles with sizes proportional to the number of responses. Add sampling points in this figure, so as to be able to see number of response closest to each sampling point.

**As recommended, we have changed this figure and try to clarify the legend (lines 292-293). On the other hand, we do not add the sampling points because they were already presented in Figure 1. Moreover, as already explained in our answer to the reviewer 1, it was not really possible to compare at a spatial scale the data resulting from citizen monitoring and those resulting from the classical monitoring because the citizen monitoring only concerned the shores of the lagoon, while the classical monitoring was performed at least 100 m far from the shoreline.**

Fig 5. For the second period (>sept/2018-2019) I would expect that values in Fig 5 should coincide with those in Fig. 3 (as people were advised to send pictures only if cyanobacteria or other worrying phenomena were observed). Why are they different? Please clarify.
**Indeed we asked to the sentinels to send picture only when cyanobacterial blooms were observed, but some people other than sentinels have continue to send pictures for example when the water color was brown. That explains that the values for the second period are not the same in Fig 3 and Fig 5.**

Fig 6. February instead of Fev. In the legend instead of CM = Citizen monitoring, authors should explain that such monitoring correspond only to potential positive observations, as explained in the secondary axis. I could not find in the text or in the legends how a potential case for cyanobacteria was selected (was it based on the information of discoloration of water, or with the photographs, or with both of them?) This information should be included in the article. Add unit of biovolume in Y axis. Why there is only info up to April 2018? If possible, extend to both periods or at least to the end of the first period, so as to increase the number of situations where there is field data and reports from citizens. Bars of CM should have within them the proportion of locations where the record was performed, village or closest location to the sampling point of cyanobacteria counts (S1 to S6) .

**We have change the figure 6 by trying to take into account all the remarks made by the three reviewers on this figure. As explained now in the M&M, it was not possible to extend the period of the institutional monitoring because this monitoring was performed in the framework of a more global monitoring of the lagoon.**

Figure 7. The information regarding how this questionnaire was undertaken is absent in the materials and methods section. Please include. Also in the figure state how many people answered this questionnaire.

**All these data are now provided in the Table 1 and the number of people answered the questionnaire has been added in Fig. 7A**

Supplementary 2. Translate to English the questions asked in the app. Even if the app is French, translate it to English so that the scientific audience can read its content.

**OK, a translation in English is now provided (See S1 Fig)**

Other comments

All other comments are inserted in the manuscript, due to the lack of line numbers in the manuscript I found it easier to write on the draft directly.

Copy of the comments in the manuscript :

Include in the results section how many meetings were performed, how many people asisted to the meetings before and after the citizenship monitoring program. How many people installed the application.

**We included a table (Table 1) providing these data. On the other hand, we don’t know how people have installed the application. During the meeting presenting this application, at least five people have installed the application but we know that our sentinels helped people to install it later.**

How many people live in the three villages? Out of the 12000 inhabitants surrounding the lagoon i the 8 villages?

**We added this data in M&M (Study site), lines 169-174.**

In each group or in total?

**In each group (corrected, see line 223)**

All participants had access to smartphones and electricity?

**Yes for the electricity, No for the smartphone but many people have one. We don’t know what is the proportion but we provided now some indications in the discussion (lines 715-719).**

So records were only performed by the sentinel or also by other members of the village? how many smartphones were used?

**It is not possible to have these data because as we described in M&M, people were able to make anonymous reports in the smartphone application. As explained now in lines 308-320, 92.8% of the data collected by the smartphone application were anonymous. But it is likely that a large part of the reports were sent by the sentinels as suggested by the GPS data.**

In the results section please say how many applications were installed.

**It is not possible to know how many applications were installed but during the meeting presenting this application, the application was installed in 5 to 10 smartphones in each village and we know from the interviews performed after these meetings that other people have installed the application latter.**

how many phones were registered?

**As previously mentioned, only few smartphone numbers were provided, most of the reports being anonymous.**

In the results sections mention how many people attended these meetings.

**These data are now available in table 1**

Can authors estimate the number of cellphones in the three villages involved?

**What we know is that most of the young men and women (<45 years old) have smartphones equipped with a camera, while most of the people >45 years old have only small cellphone equipped with radio, not with a camera. We added few sentences on that in the M&M (lines 715-719).**

Mention that the program run out fo funds. At first when I did not know this information, until I read it in the Discussion, i thought why such a short time frame was selected.

**We better explain now why we only get data on phytoplankton community during seven months (lines 363-368).**

How many cellphones where involved. The question is if the three sentinels produced all questionnaries or if many citizens were involved in completing the questionaries. Only in the discussion i understood that most questionaries were done by three persons.

**Again, due to the anonymous option for the smartphone application, it is not possible to know the relative contribution of the sentinels and of other people in the citizen monitoring. But regarding to the GPS location, we estimated that a large part of the contributions were done by the sentinels, in particular for the Débarcadère village.**

This sentence (This difference in the number of collected questionnaires during the two periods was expected since we changed there commendation for the sentinels from sending a picture at least once a week…) should be in discussion, not here in results.

**We think that it is better not to change the place of this sentence for the overall understanding of this paragraph.**

remove well.

**Ok, removed…**

Add full stop.

**Ok, added**

This part of the sentence should be located in Discussion not in Results.

**Ok, this part of the sentence has been removed and we added one sentence on this issue in the discussion**

Looking into the data of the application the differences in color can be due to cyanobacteria or due to organic matter. How could you differentiate one from the other?

**The color of the water during cyanobacterial blooms is different from the color of water with high amount of organic matter, much more green during the blooms. Moreover, during cyanobacterial blooms it is very likely to see in water some pellets corresponding for example to Microcystis colonies. If you look at supplemental Figure 2, it was clearly visible. But we agree with you that sometimes, you can have a green color in the water due to other phytoplankton microalgae than cyanobacteria.**

this village is closest to S1, but in the graph the bloom is largest in S4 which is more close to Aghien T. village. I would expect a discussion about this in the discussion section.

**It was difficult to really compare the data providing by the citizen monitoring and those resulting from the classical approach performed by the Pasteur Institute. The two main reasons are that (i) it is well known that you can observed accumulation of cyanobacteria on the shore of the lake but quite nothing 50 m away of this shore and (ii) the spatial distribution of the cyanobacterial blooms can change very quickly (few hours) due to wind and current effects. For a definitive validation of the data provided by the citizen monitoring, it should have been necessary to sample water at the places where the pictures were taken. Knowing that, we have choose to limit the comparison between these data in term of co-occurrence between the monthly reports of cyanobacteria by citizens and the total biovolumes of cyanobacteria estimated at the end of each month by the Pasteur Institute. You can see all the changes that have been made in the result and discussion parts concerning this issue (lines....).**

Remove "Evolution of the", start with Number

**Ok, we changed the legend of this figure.**

This “suggesting this approach has the potential to contribute to the monitoring of cyanobacteria inAfrica” should go in discussion not in results.

**Ok we removed the end of the sentence**

the first two sentences should go in discussion.

**Ok, they have been removed**

There is no information in materials and methods regarding this questionary.

**As explain now in the M&M, we used data from a large questionnaire performed in the framework of researches in social sciences on the populations living around the lagoon. We only used two questions, which are now provided in supplemental data (see S1 Table)**

S1 from Fig. 6 seems of better quality than S4. Mention that despite marked differences in teh water quality the perception per village shows an opposite trend.

**In fact, when looking on the whole data set collected from December 2016 to April 2018 (a publication is in preparation on these data set which concerns many physico-chemical and biological parameters), we show that in agreement with the perception of people living in Débarcadère village, the water quality is worse in this area than in the rest of the lake.**

Add a reference to Fig. 7b for fish

**There is no reference for fish. The decrease in fish catch is experienced by the local fishermen.**

rephrase this sentence. as seen in fig. 7b. both color of water and decreased fishe catch are seen as very important issues in the lagoon

**Yes, we agree with you. The sentence has been changed.**

This sentence (The focus groups and qualitative interviews also provided…) should be relocated into the discussion.

**We do not agree with you because the interpretation of the changes occurring in the water color is also a result.**

This paragraph (The initial focus groups and the restitution events represented…) should be relocated in to the discussion.

**Again we do not agree with you because we provided new data in this paragraph that results directly from the focus group and qualitative interviews.**

this information should be presented in materials and methods, when explaining the available data.

**Yes, we explained now in the M&M how these data were obtained**

The use of satellite images can help substantially in the monitoring of the Lagoon. Once implemented the cost is very low and can be used to assess the role of citizenship monitoring and the detection of blooms.

**We agree that the use of satellite images can be useful for the monitoring of cyanobacteria but in the local context, it seems difficult to imagine to use this kind of approach due to the strong limitation of financial resources.**

did not show, number was too low. change the words, use words such as seem, suggest, not show. here authors need to aknowledge that the number of data they have are too low.

please rewrite this sentece. i can see the value of citizen monitoring which has no cost. put it in a separate sentence and elaborate longer.

**We have changed this paragraph in our discussion**

coupled with education and capacitation programs. Please include.

**Ok, it is now included**

remove extra space

**Ok, done**

This information should be mentioned before in the manuscript, for example in Results and show examples of photographs, at least in supplementary material. I checked on the web page and by looking at the photos alone the color of the water. **Yes we agree that the quality of numerous pictures was to low to really see the color of the water event if sometimes the green color and the cyanobacteria were clearly visible for some of them (see S2 Fig). We added a sentence in the result section (lines 459-463)**

This information should be mentioned before, in the results section. How many smartphones participated in gathering the information. If 90% of people have access to cellphones, as stated somewhere in the draft, then if only 3 chiefs produce the data, then the participation of the citizens is low.

**We do not know how many people and how many smartphone have contributed to the monitoring because most of the report were anonymous (see our previous answer on this question).**

How many people in the villages had access to cellphones? How many women use cellphones?

**We already provided an answer to this question (see above)**

This information should be presented in the REsults section as part of the results of the focus group meetings

**We do not understand why this information should be presented in the result section. We discussed in this paragraph the fact that depending on the choice of this sentinel (which was made by the chiefdom), the investment made by each sentinel was very various, showing the importance of this choice.**

Here you can suggest to identify good teachers in each village to take the leading role (sentinels) but also as the role of disseminating the importance and results to students and to their parents (either directly or throught their kids).

**It is more complicated than that. It was not possible for us to choose the sentinels in the villages because this is clearly a responsibility of the chiefdom. That explains why we have so different people as sentinel in the three villages.**

Reviewer #3: Author’s designed, implemented and evaluated a project of participatory monitoring of cyanobacteria blooms through the use of a smartphone application, working with the populations of three villages located on the shoreline of a freshwater lagoon located near Abidjan city (Lagoon Aghien in the Ivory Coast). The aim was to analyze the efficiency of such citizen science approach, comparing with classical monitoring, the potential constraints of the approach, and the contribution to citizen awareness about cyanobacteria blooms and their health consequences. Author’s claimed that the participatory approach provided accurate data compared with classical monitoring, and in addition, showed great improvements to the understanding and awareness of local populations regarding water quality issues.

In my opinion, the work is interesting, although the limited data of the classical monitoring make it less relevant than it could have been. It’s a shame that the classical monitoring was unilaterally finished by French institutions. In my personal opinion, there is limited data to account for a solid comparison. In this sense, in my opinion the work is quite limited about the efficacy of the participatory detection of cyanobacteria blooms.

**The classical monitoring was not stopped unilaterally by French institutions (see our response below). We fully agree that it was not possible to perform a solid comparison between citizen monitoring and institutional monitoring but our paper show that it is really possible to involve people in Africa in the monitoring of freshwater ecosystems, to obtain potentially promising findings on their ability to detect cyanobacterial blooms and finally that this kind of approach is a powerful tool to increase the awareness of local populations to the issue of water quality.**

Globally, my recommendation is to consider some major and minor revisions, some of them are important to clarify or improve the manuscript.

Majors

1. The comparison of the participatory approach with the classical one shows some caveats that don’t totally reflect the efficacy of the participatory approach about the detection of cyanobacteria blooms. For example, it appears to be a threshold in the detection of water color changes associated with cyanobacteria blooms. Some smaller blooms were not detected by local people. This issue have to be more addressed on the text.

**We agree with your comment and we made changes both in the “result” and “discussion” sections**

2. “The importance of water color for the assessment of water quality by inhabitants was seen by us as a positive signal for the implementation of participative monitoring of cyanobacteria blooms.” Results section.
I don’t agree with that. A limitation is that before smartphone application you don't know if they use water color as indicator of water quality. That's a limitation to inform about the awareness associated with monitoring. Please remove or modify accordingly if you have more information previous to the beginning of participatory monitoring.

**We do not understand this remark because the responses of people to our questionnaire has shown that they clearly associated changes in water quality with the water color (see lines 588-589). In particular brown and green water were clearly identified as particular periods.**

3. I think that Figure 6 and the comparative analysis of monitoring programs should be improved. It is desirable to have some quantitative measurement of agreement between the classical and the participatory approaches. I suggest some kind of analysis that allow subjective/objective comparisons (e.g. ROC curves), or some kind of association/classification analysis.

**We have made changes in Fig 6. As we explained now in the MS, due to spatial and temporal differences in the strategy of the two monitoring approaches, it is difficult to compare the data they provided. For this reason, we can just check as we did in Fig 6, if there was some consistency between these data.**

Meanwhile, the plot is not very straightforward to understand it, in particular regarding biovolume counts in each station. Related with this, S5 and S6 have the same color? Color codes could also be improved to facilitate identification of stations. Lack of units in y-axis.

**We have changed the figure**

Minors

Material and Methods sectioned,

4. “As shown by this figure, the participatory monitoring benefited from sociological
investigations conducted within the framework of the WaSAf program…”.
This is not clear in Figure 2. Where is mentioned in Fig. 2 the previous sociological research? Meetings are not only part of sociological research, they can be part of any research. Please modify the text or the Figure accordingly.

**We agree that it was not enough clear and we have changed this paragraph in M&M**

5. Figure 2. It is not clear from the figure or the text why the steps are divided in three blocks without a correspondence with the three main titles of the boxes. Why is it? I think that it is confusing, I suggest to edit the figure or to be clearer with that at the legend. For example, Phase 1, preparation, have 2 items and a previous one: 1) first meeting to choice villages, 2) organization of focus groups, 3) meetings and provision of flyers of smartphone application; but the phase was divided in two boxes, the first two items in a box, and the third item on a second box. Why? The same thing with the rest of the phases/boxes.

**Ok, we changed this picture in order to make it more clear and logical**

6. “Meetings and interviews with local community leaders and chiefdom and with
inhabitants of the three villages..”
Please check syntaxes. A comma is lacking somewhere

**OK, corrected**

7. “prospectus (Lagoon Health Bulletin) were also distributed in schools and villages.”
Prospectus were done by scientist or also by local actors, such as sentinels? Please clarify.

**OK, it is now clarified**

8. ” we also conducted qualitative interviews with the three sentinels and other volunteers”.
What kind of interview did author's performed? Informal? Semi-structured? Please clarify the kind of interview and any further details to understand the kind of responses given by interviewers.

**OK, we provide now more details on these methodologies**

9. “The participatory monitoring was organized in two periods…”
Why did authors choose this approach? It was top-down (scientist to local people) or it was discussed or decided with local actors? Can you clarify the approach?
**Yes, we provided now more detailed on this strategy lines 321-329**

Results section

10. What are the violet points in Figure 4? Please clarify or remove them.

**Also in agreement with the first reviewer, we changed this figure and its legend in order to make them more easy to understand**

11. Figure 5. To have a better picture of the participatory monitoring of blooms, authors can include within the graph several photos sent by sentinels corresponding to different moments of the monitoring dynamics.

**Unfortunately, it is not possible to download the pictures with a sufficient definition for a publication in the paper. Each image has a definition of 140 dpi while 300-600 dpi has required for PLoS ONE. All the pictures are still available on the web site and we added a Supplemental Figure (S2 Fig) presenting two images of blooms taken by the sentinels.**

Discussion section

12. “This first citizen project based on the use of a smartphone application for the monitoring of cyanobacterial blooms in Africa shows that there opportunity for such approaches.”
Did authors mean “there is opportunity”? Please correct.

**Yes, we have corrected this sentence**

13. Limitations of the program. Authors have limited data to take conclusions about participatory monitoring. Some blooms were not recorded by participants, is not just technical problems regarding geolocalization. This is an important issue and has implications for future monitoring programs.

**We agree that this pilot study only provided first indications on the potential of a citizen monitoring for the survey of cyanobacteria. That’s the reason why we added a new paragraph at the end of the discussion and also that we moderate our purposes on the efficiency of this monitoring in result and discussion sections.**

14. What was the reaction of local people when French institutions unilaterally finished the monitoring? May be it would be interesting to mention at the discussion section.

**We did not write that French institutions unilaterally stopped the monitoring. The Pasteur Institute of Abidjan had got funds for a 18 month monitoring of the lagoon which ended in April 2018. It was not possible to find new funds from Ivory Coast institutions in order to continue this institutional monitoring.**

15. Have sentinels stopped to send reports so far? Which is the long-term sustainability of the program? If author's can expand this observation it would be great.

**No, two of them continue to send pictures, even if the program is at the end. But we are working on the implementation of an observatory of the lagoon Aghien with the goal to to perform a sustainable monitoring on the lagoon, including classical and participatory approaches. Hope that it will be possible to do in the next months!**

16. I fully agree with the content of last paragraph but I think this is not a conclusion technically speaking. I suggest that in order to maintain the content in the text, please modify the paragraph and/or add a conclusion relative with the goals of the work.

**OK, we added a new conclusion at the end of the paper.**
